# Differentially Expressed Genes and Molecular Susceptibility to Human Age-Related Diseases

**DOI:** 10.3390/ijms24043996

**Published:** 2023-02-16

**Authors:** Svetlana Shikhevich, Irina Chadaeva, Bato Khandaev, Rimma Kozhemyakina, Karina Zolotareva, Anna Kazachek, Dmitry Oshchepkov, Anton Bogomolov, Natalya V. Klimova, Vladimir A. Ivanisenko, Pavel Demenkov, Zakhar Mustafin, Arcady Markel, Ludmila Savinkova, Nikolay A. Kolchanov, Vladimir Kozlov, Mikhail Ponomarenko

**Affiliations:** 1 Institute of Cytology and Genetics, Siberian Branch of Russian Academy of Sciences (SB RAS), Novosibirsk 630090, Russia; 2 The Natural Sciences Department, Novosibirsk State University, Novosibirsk 630090, Russia; 3 Research Institute of Fundamental and Clinical Immunology (RIFCI) SB RAS, Novosibirsk 630099, Russia

**Keywords:** human, age-related disease, molecular marker, *Rattus norvegicus*, RNA-Seq, qPCR, differentially expressed gene, meta-analysis, correlation, principal component, bootstrap

## Abstract

Mainstream transcriptome profiling of susceptibility versus resistance to age-related diseases (ARDs) is focused on differentially expressed genes (DEGs) specific to gender, age, and pathogeneses. This approach fits in well with predictive, preventive, personalized, participatory medicine and helps understand how, why, when, and what ARDs one can develop depending on their genetic background. Within this mainstream paradigm, we wanted to find out whether the known ARD-linked DEGs available in PubMed can reveal a molecular marker that will serve the purpose in anyone’s any tissue at any time. We sequenced the periaqueductal gray (PAG) transcriptome of tame versus aggressive rats, identified rat-behavior-related DEGs, and compared them with their known homologous animal ARD-linked DEGs. This analysis yielded statistically significant correlations between behavior-related and ARD-susceptibility-related fold changes (log_2_ values) in the expression of these DEG homologs. We found principal components, PC1 and PC2, corresponding to the half-sum and the half-difference of these log_2_ values, respectively. With the DEGs linked to ARD susceptibility and ARD resistance in humans used as controls, we verified these principal components. This yielded only one statistically significant common molecular marker for ARDs: an excess of Fcγ receptor IIb suppressing immune cell hyperactivation.

## 1. Introduction

Seven years ago, the World Health Organization (WHO) defined the Healthy Ageing Framework [1] and declared the decade between 2020 and 2030 as the Decade of Healthy Ageing [2]. Because a standard definition of age-related diseases (ARDs) has yet to be agreed upon, epidemiologists differentiate between, on the one hand, all non-infectious diseases with a reliance on incidence rates rising exponentially with age, no matter the lifespan, and, on the other hand, the diseases that start in early life and have stable or lowered incidence rates in the elderly [3]. Given this lack of strict classification, many age-related diseases can be named as ARDs: sarcopenic obesity [4], homeostasis dysregulation [5], subfertility [6], lipodystrophy [7], sarcopenia [8], macular degeneration [9], chronic inflammation [10], osteoarthritis [11], endothelial dysfunction [12], tissue senescence [13], cancer [14], atherosclerosis [15], cardiovascular diseases [16], chronic kidney disease [17], stroke [18], frontotemporal dementia [19], Alzheimer’s [20], and Parkinson’s [21], to mention some. Moreover, many other pathologies can contribute to ARDs: amyotrophic lateral sclerosis, to motoneuronal aging [22]; mitochondrial dysfunction, to aging as such [23]; vascular atherosclerosis, to cellular senescence [24]; hypertension, to vascular aging [25]; thalassemia, to myelodysplastic syndrome [26]; cancer, to immune system aging and vice versa [27]; and circadian rhythm disorder, to aging as such [28]. Furthermore, some diets enriched for fat [29], calcium [30], or citrates [31] can provoke ARDs, as do long-term hunger, malnutrition, anorexia, and appetite loss [32]. By contrast, moderate physical exercise [33], short-term fasting [34], and low-dose aspirin [35] can prevent such diseases.

This could be why mainstream transcriptome-profiling studies of ARD susceptibility versus ARD resistance in human volunteers [33,36,37,38,39,40,41,42,43] and animals [30,32,35,44,45,46,47,48,49,50,51,52,53,54,55,56,57,58,59,60,61,62,63,64] are focused on the differentially expressed genes (DEGs) that are specific to gender, age, tissues, and pathogeneses. All previous research contributes to progress in predictive, preventive, personalized, participatory (4P) medicine [65] and helps us understand where, how, why, when, and what disorders can affect people depending on their genetic background, medical history, and lifestyle. Because none of us is able to escape an ARD, we expected that a meta-analysis of all available ARD-linked DEGs would eventually reveal the most common, universal theranostic molecular marker that will be permanently available in any tissue of anyone’s organism.

Because hypertension contributes to vascular aging [66] and vice versa, we have previously studied, within this mainstream paradigm, the inbred ISIAH (Inherited Stress-Induced Arterial Hypertension) rat strain [67] and sequenced transcriptomes in the brain stem [47], hypothalamus [48], renal medulla [49], renal cortex [50], and adrenal glands [51], with WAG rats used as controls. Additionally, we have recently obtained and compared transcriptomes of the midbrain tegmentum [68] in gray rats of a tame and an aggressive outbred strain [69,70,71,72]; this allowed us to identify, in in silico settings, potential molecular markers for neoteny, which has a power to reverse ARDs [73]. Furthermore, we have recently profiled transcriptomes in the hippocampus [74] of tame versus aggressive rat strains, in which deficient β-protocadherins and β-hemoglobin were found to be the statistically significantly most common theranostic molecular markers for ARD-related hypertension. That is why we sequenced, in the same way, one more transcriptome, that of the periaqueductal gray matter (PAG), in the tame versus aggressive rats, identified the corresponding behavior-related PAG-associated DEGs in them, and compared these DEGs with their homologous PubMed-based [75] ARD-linked DEGs in animals [30,32,35,44,45,46,47,48,49,50,51,52,53,54,55,56,57,58,59,60,61,62,63,64,68,74] and humans [33,36,37,38,39,40,41,42,43] to find out whether there are invariant molecular markers for such diseases among them.

## 2. Results

### 2.1. RNA-Seq and Mapping to the Reference Rat Genome

We focused on the PAG because the activity of this brain structure contributes to elevated pain tolerance [76], sociability, intelligence (IQ), and humor processing with increasing age [77]. In fact, our best hope was that if we had had all possible ARD molecular markers at hand, we would have eventually understand which of them might be the best helpers to relieve the suffering of ARD patients. We profiled the PAG transcriptome of three tame adult male gray rats (*Rattus norvegicus*) against three aggressive conspecifics on an Illumina NextSeq 550 system (see Section 4.2). The rats came from two outbred strains, one tame and one aggressive, maintained at the Institute of Cytology and Genetics of the Siberian Branch of the Russian Academy of Science [72,78] for more than 90 generations using the glove test [79]. The rats were not consanguineous (see Section 4.1). First, we sequenced 210,128,758 reads each 75 nt in length and deposited them in the NCBI SRA database [80] (ID PRJNA668014) (see Table 1). Next, we chose from among them 177,608,837 raw reads (84.5%) via mapping to the reference rat genome RGSC Rnor_6.0, UCSC Rn 6 July 2014 (Table 1). Then we identified 14,039 genes expressed in the PAG of the studied rats. Finally, we selected 39 DEGs using Fisher’s Z-test with the Benjamini correction for multiple comparisons and discarded hypothetical, tentative, predicted, uncharacterized, or non-protein-coding genes to minimize the false-positive error rates (Table 1 and Table 2).

### 2.2. Quantitative PCR (qPCR)-Based Selective Verification of the Novel PAG-Related DEGs of the Tame and Aggressive Rats

From the same two strains of rats, we took 16 additional unrelated animals: eight tame and eight aggressive, each scoring 3.5 and –3.5, respectively, on a scale spanning from –4 (most aggressive) to +4 (least aggressive) in the glove test [79] run 1 month before the PAG specimens were sampled (Table 3).

Next, from among the 39 DEGs shown in Table 2, we selected *Ascl3* and *Defb17* (for details, see Table 3). Table 3 contains our qPCR data on these genes in the PAG of the tame and aggressive rats (see Section 4.4). These PCR data appear as the arithmetic mean ± standard error of the mean of *Ascl3* and *Defb17* expression levels normalized to those of three reference genes, *B2m* (β-2-microglobulin) [81], *Hprt1* (hypoxanthine phosphoribosyltransferase 1) [82], and *Rpl30* (ribosomal protein L30 [83]), in triplicate, according to the guidelines [84]. The rightmost column of Table 3 shows arithmetic mean estimates of the expression levels of *Ascl3* and *Defb17* in the PAG of the tame and aggressive rats.

**Figure 1:**
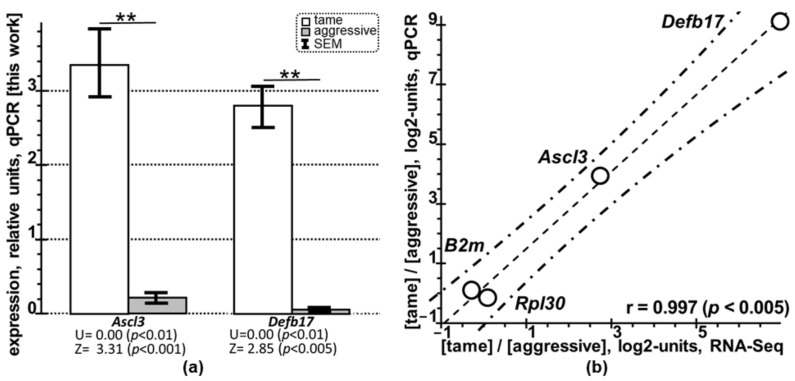
qPCR-based selective verification of the DEGs identified with RNA-Seq in the PAG of tame versus aggressive rats. Legend: (**a**) Both DEGs, *Ascl3* and *Defb17*, are significantly overexpressed in the PAG of the tame adult male rats (white bars) compared to their aggressive conspecifics (gray bars). Bar height: mean; error bars: standard error of the mean [SEM]; the double asterisk (i.e., double character “**”) denotes statistical significance at *p* < 0.01 according to nonparametric Mann–Whitney *U* test and parametric Fisher’s Z-test. (**b**) Pearson’s linear correlation between the relative expression levels of *Ascl3*, *Defb17,* and the reference genes [*B2m* (β-2-microglobulin) and *Rpl30* (ribosomal protein L30) appearing as circles on the plot] in the tame versus aggressive rats is statistically significant, whether measured experimentally using RNA-Seq (*x*-axis) or qPCR (*y*-axis) and expressed on the log_2_ scale (see “Materials and Methods”). Dashed and dot-dash lines denote linear regression and its 95% confidence interval boundaries calculated using STATISTICA (Statsoft^TM^, Tulsa, OK, USA); r and *p* are Pearson’s linear correlation coefficient and its statistical significance, respectively.

As can be seen from Figure 1a, both *Ascl3* and *Defb17* are overexpressed in the PAG of the tame rats (white bars) compared to the aggressive rats (gray bars) according to our qPCR data, this overexpression being significant (*p* < 0.01, double asterisk) according to both the nonparametric Mann–Whitney *U* test and the parametric Fisher’s Z-test.

This finding independently supports our RNA-Seq data (Table 2). Finally, Figure 1b shows Pearson’s linear correlation (r = 0.997, *p* < 0.005) between the log_2_ values (“log_2_” hereinafter means “the log_2_-transformed ratio of the expression level of a given gene between tame and aggressive rats under the given experimental conditions”) for four genes, *Ascl3*, *Defb17*, *B2m*, and *Rpl30* (all appearing as open circles), within the RNA-Seq (*x*-axis) and qPCR (*y*-axis) datasets, both obtained here independently. This is one more piece of independent evidence in support of the relevance of our RNA-Seq data (Table 2).

### 2.3. Comparison of Known Animal ARD-Linked DEGs with Their Homologs among 39 Novel PAG-Related DEGs of the Tame and Aggressive Rats

We retrieved all transcriptomes of animals with ARD susceptibility and ARD resistance from the PubMed database [75] and collected 43 animal-based human ARD models (Table 4).

**Figure 2:**
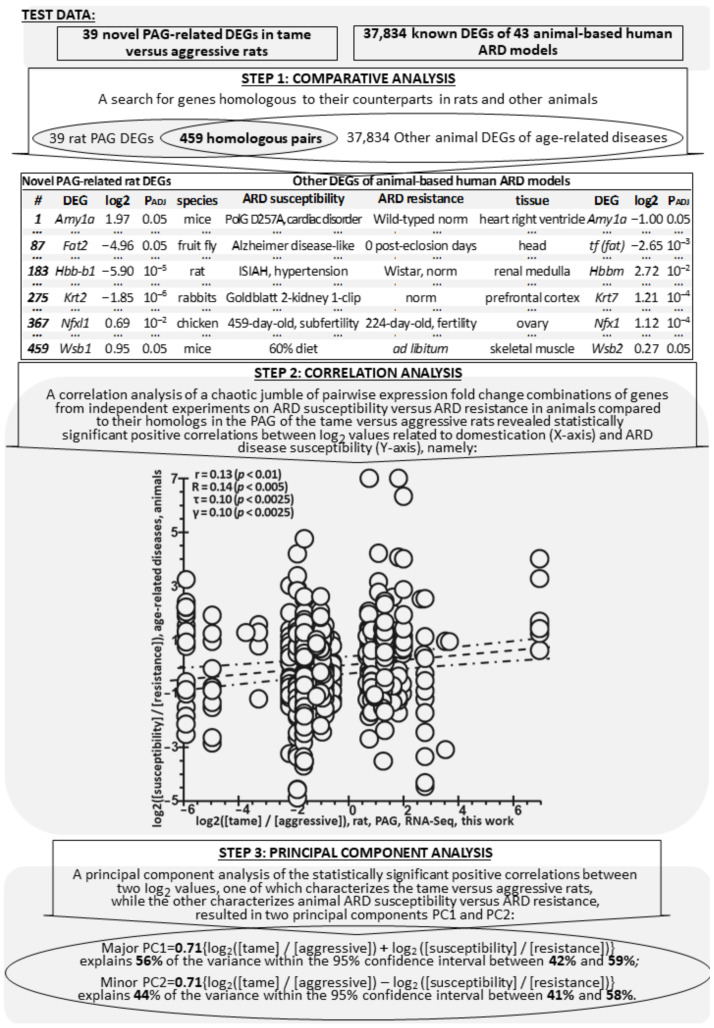
Comparison of the novel DEGs in the PAG of the tame and aggressive rats with the known DEGs related to animal ARD susceptibility and resistance. Legend: See the legend to Figure 1 and the footnote to Table 4; the *x*- and *y*-axes correspond to columns iii and x of Table S1; circles correspond to rows of Table S1; R, τ, and γ are the coefficients of Spearman’s rank correlation, Kendall’s rank correlation, and the Goodman–Kruskal generalized correlation, respectively, calculated using STATISTICA (Statsoft^TM^, Tulsa, OK, USA); PC1 and PC2: principal components calculated in the bootstrap-based refinement mode of the PAST4.04 software [85].

The total number of DEGs found in 22 original works [30,32,35,44,45,46,47,48,49,50,51,52,53,54,55,56,57,58,59,60,61,62,63,64] was 37,834 in 17 tissues of five animal species. Figure 2 shows our procedure of comparison of 37,834 animal ARD-linked DEGs (Table 4) with the 39 PAG-related DEGs of the tame and aggressive rats (Table 2). In this figure, a Venn diagram and a table explain the procedure that led us to the following conclusion: there is a correlation between the expression of homologous genes in the tame rats and the ARD-susceptible animals. At the first step, we compiled 459 pairs of homologous DEGs, where one of 39 DEGs was taken from Table 2 and its homolog was chosen from among 37,834 DEGs in Table 4, both now being present in Table S1 (see “Supplementary Materials”). At the second step, we for the first time found the following statistically significant correlations between behavior-related and disease-susceptibility-related log_2_ values of the homologous animal DEGs: Pearson’s linear correlation (r = 0.13, *p* < 0.01), Spearman’s rank correlation (R = 0.14, *p* < 0.005), Kendall’s rank correlation (τ = 0.10, *p* < 0.0025), and the Goodman–Kruskal generalized correlation (γ = 0.10, *p* < 0.0025) (Figure 2). These correlations suggest a certain similarity in the expression patterns of the novel PAG-related DEGs between the tame and aggressive rats and a similarity in the expression patterns of their homologous DEGs between ARD-susceptible and ARD-resistant animals. The biological sense of these similarities was elucidated at the final step. We processed entries in Table S1 with principal component analysis in the bootstrap mode with the freely available toolbox PAST4.04 [85]. In the result, we found two principal components, major PC1 and minor PC2, corresponding to the half-sum and the half-difference of the behavior-related and ARD-susceptibility-related log_2_ values for the homologous animal DEGs, respectively. Here, the half-sum of the behavior- and ARD-associated log_2_ values, which appears as the major PC1, implies that the sense of the significant positive correlations found between these log_2_ values at step 2 is that their signs in the novel PAG-related rat DEGs and in the known ARD-linked animal DEGs are matching. Their formulas and 95% confidence intervals are given in the bottom part of Figure 2.

### 2.4. Animal ARD-Linked DEGs Are Relevant to Humans

**Figure 3:**
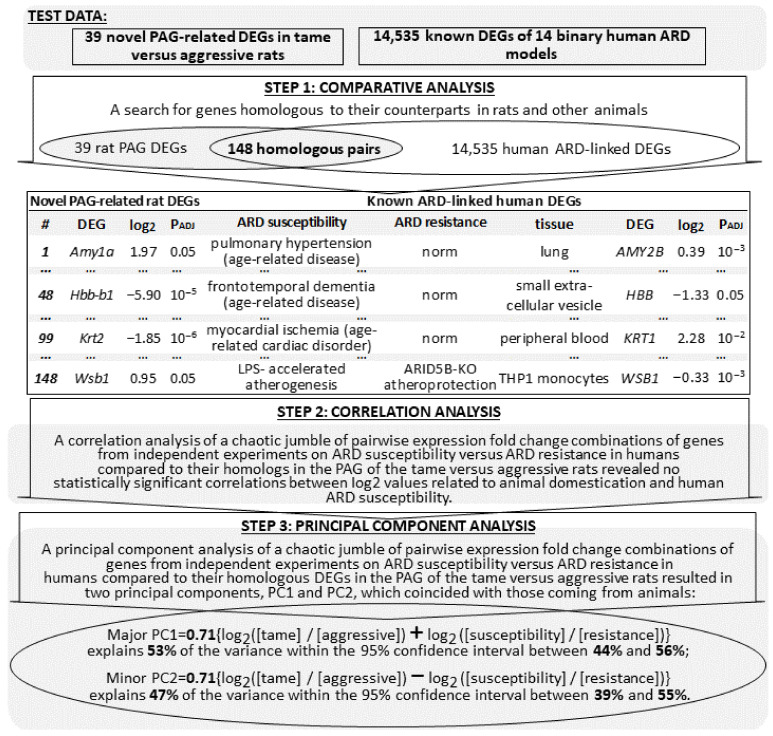
Animal ARD-linked DEGs are relevant to humans. Legend: See the legend to Figure 2 and the footnote to Table 2.

We additionally searched for all the PubMed DEGs in ARD patients and otherwise healthy volunteers [75] (see Table 5). The total number of human ARD-linked DEGs found is 14,535 in ten tissues from 14 binary “susceptibility versus resistance” models of human ARDs (the rightmost column of Table 5) [33,36,37,38,39,40,41,42,43]. Figure 3 illustrates a modification of the previous procedure, with the animal ARD-linked DEGs replaced by their human counterparts (Table 5).

The results serve as independent control medical data (Table S2). Although no correlation was found between the behavior-related log_2_ values for the novel PAG-related rat DEGs and the ARD-susceptibility-related log_2_ values for their human homologs, their half-sum and half-difference corresponded to principal components PC1 (major) and PC2 (minor) within their quite similar 95% confidence intervals (Figure 3). This finding confirmed the results of our meta-analysis of ARD susceptibility versus ARD resistance in animals.

### 2.5. Searching for ARD Molecular Markers among Human Genes Orthologous to 39 Novel PAG-Related DEGs of the Tame and Aggressive Rats

First, we used the PubMed database [75] and characterized each of the 39 novel PAG-related DEGs of the tame and aggressive rats (Table 2) by answering the question as to how under- or overexpression of their human orthologs can aggravate or alleviate ARDs [86,87,88,89,90,91,92,93,94,95,96,97,98,99,100,101,102,103,104,105,106,107,108,109,110,111,112,113,114,115,116,117,118,119,120,121,122,123,124,125,126,127,128,129,130,131,132,133,134,135,136,137,138,139,140,141,142,143,144,145,146,147,148,149,150,151,152,153,154,155,156,157,158,159,160,161,162,163,164,165,166,167,168,169,170,171,172,173,174,175,176,177,178,179,180,181,182,183,184,185,186,187,188,189,190,191,192,193,194,195,196,197,198] (Table S3). Next, for each novel PAG-related rat DEG (Table 2), we determined the sign of the behavior-related log_2_ value and found how many of their homologs in the ARD-susceptible and ARD-resistant subjects have matching signs of the ARD-susceptibility-related log_2_ value (*n* = N_PC1_) and how many have opposite signs (*n* = N_PC2_). This is because the principal components PC1 and PC2 correspond to the half-sum and the half-difference, respectively, of these log_2_ values (Figure 2 and Figure 3). Table 6 shows these figures (N_PC1_ and N_PC2_) alongside their statistical significance estimates according to the binomial distribution both before (*p*-values) and after (*P_ADJ_* values) Bonferroni’s correction for multiple comparisons.

As can be seen from this table, only one of the 39 novel DEGs in the PAG of the tame and aggressive rats is linked with PC1, namely *Fcgr2b* encoding Fcγ receptor IIb, which suppresses the hyperactivation of immune cells [199] (Table 7). Entries in this table suggest that the immunoregulatory DEGs homologous to rat *Fcgr2b* are significantly overexpressed in ARD-susceptible human and animal subjects compared to their ARD-resistant peers [35,39,43,51,55,56,58]. This leads us to propose that an excess of the immunoregulators homologous to rat Fcgr2b might be regarded as a candidate theranostic molecular marker for human ARDs.

### 2.6. Can Human Fcγ Receptor IIb Upregulation Serve as a Theranostic Molecular Marker for ARDs?

The following facts and hypotheses were thought to be relevant to this question: (i) the number of human infections increases when new wild animals are domesticated and become a bridge between the anthropogenic environment and wildlife [200], (ii) stray cats live longer than pet cats due to stronger disease resistance [201], and (iii) human evolutionary origin involves self-domestication [202], although this hypothesis is still debatable [203].

With this information in mind, we searched PubMed [75] and retrieved all available transcriptomes of domestic animals compared to their wild conspecifics (Table 8). As can be seen from the bottom line of Table 8, RNA-Seq data represent 2866 DEGs in eight tissues of seven domestic animal species compared to their seven wild conspecifics studied in ten original experimental works [68,74,204,205,206,207,208,209,210,211].

By looking at the entries in the left half of Table 9, it becomes clear why underexpression of the human *FCGR2B* gene, which is orthologous to the rat *Fcgr2b* gene (this being the only gene we have identified as a theranostic molecular marker for ARD; Table 9), alleviates such diseases [117,118], while its overexpression aggravates them [119,120].

From among the 39 novel PAG-related rat DEGs (Table 2) and these 2866 known animal DEGs (Table 8), we selected seven animal genes homologous to the rat *Fcgr2b* gene (the right half of Table 9). Furthermore, we transformed the log_2_ value characterizing the animal DEGs into either underexpression or overexpression of these animal genes after the domestic and wild animals split from their most recent common ancestor, according to the commonly accepted phylogeny concept [212,213,214,215,216]) (Table 9).

Downregulation of the human gene *FCGR2B*, which provides protection against infection, alleviates ARDs [117,118] and matches downregulation of the homologous genes *Fcer2*, *Fcgr2b*, *Fcgr3a*, *Fcgr3b*, *Fcrl1*, and *Fcrl2* in wild rabbits [206], aggressive rats [68,204], and aggressive foxes [208] after they and their domestic conspecifics split from their most recent common ancestor (Table 9).

By contrast, an excess of this immunosuppressor in humans aggravates the cellular-senescence-related immunogenic disease [119,120] and corresponds to the excess of its homologous immunoregulators in cavies [205], domestic rabbits [206], tame rats [68,204], and tame foxes [208] during their microevolution (Table 9).

The left half of Table 10 summarizes the data shown in Table 9 in the form of a 2 × 2 contingency table, where this difference is statistically significant according to both Pearson’s χ^2^ test (*p* < 0.01) and Fisher’s exact test (*p* < 0.05).

In this regard, upregulation of the immunoregulators homologous to the rat *Fcgr2b* gene can serve as a theranostic ARD molecular marker permanently available in any tissue of anyone’s organism.

## 3. Discussion

### 3.1. Overexpression of Human Genes Homologous to the Rat Fcgr2b Gene Identified as A Molecular Marker for ARDs Is Consistent with Overexpression of Known ARD-Linked DEGs

We have found that upregulation of the human immunoregulators homologous to the rat *Fcgr2b* gene can serve as a theranostic ARD molecular marker permanently available in any tissue of anyone’s organism (see Table 7). As can be seen from Table 7, the immunoregulators homologous to the rat *Fcgr2b* gene are excessive in all tissues of all ARD-affected subjects (humans and animals) with only one exception: FCGR1B deficiency in the lungs of a patient with fibrosis in pulmonary arterial hypertension [39] (Table 7: line #12). This implies that an excess of the human immunoregulators homologous to the rat *Fcgr2b* gene identified in this work as being a molecular marker for age-related human diseases is consistent with the ARD-linked DEGs found independently by other authors.

### 3.2. Excess of Rat Fcgr2b Identified as A Molecular Marker for ARDs Is Consistent with Independent RNA-Seq Data on the Rise in the Levels of Murine Fcgr2b in Astrocytes with Age

**Figure 4:**
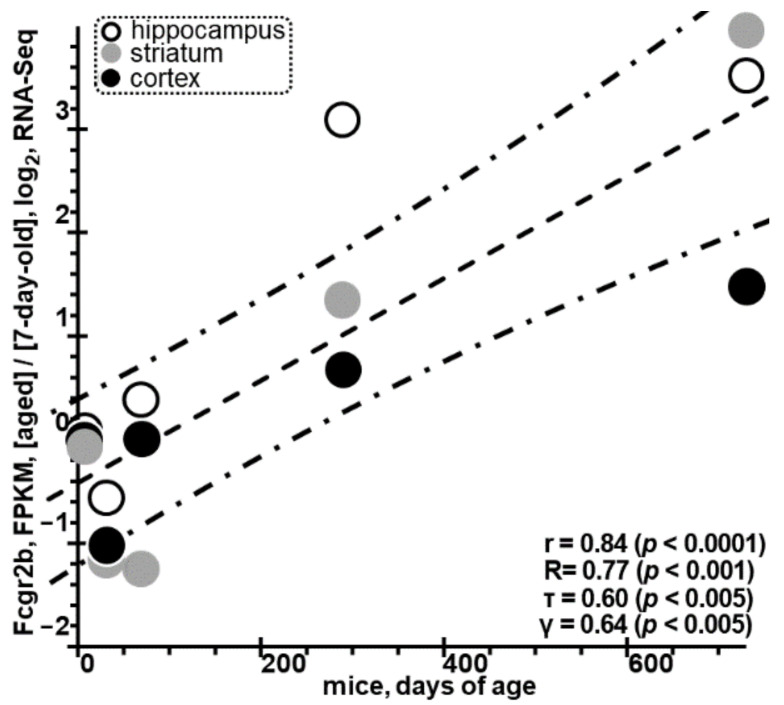
An RNA-Seq experiment run within the mouse model of human ARDs [217] revealed a statistically significant increase in *Fcgr2b* expression in astrocytes from the hippocampus (open circles, ○), striatum (gray circles, ●) and cortex (black circles, ●) with increasing age, providing independent in vivo evidence in support of this finding. Legend: See the legend to Figure 1 and Figure 2; FPKM, fragments per kilobase of transcript per million mapped reads [217].

We will discuss this result in comparison with independent RNA-Seq data [217] from a human ARD model using mice aged 7 days, 32 days, 10 weeks, 9.5 months, and 2 years. Although murine *Fcgr2b* was not identified as a DEG within the model in question [217], we have found a statistically significant increase in the expression of this gene in astrocytes (see Figure 4) from the hippocampus (open circles), striatum (gray circles), and cortex (black circles) of mice with increasing age. This could be regarded as an in vivo piece of independent direct evidence in support to our findings in this work.

### 3.3. Stabilizing Selection Preserves the Expression Pattern of the Human FCGR2B Gene Orthologous to the Rat Fcgr2b Gene, a Molecular Marker for ARDs

Because it now seems interesting to discuss how such an extraordinary expression pattern—that is, with levels increasing with age—can have been preserved, we will examine the proximal promoter of the human *FCGR2B* gene (see Figure S1). As can be seen from Figure S1a, the current build (No. 155) of the dbSNP database [218] contains only one single-nucleotide polymorphism, SNP rs780467580, within the 70 bp proximal promoter of the human *FCGR2B* gene. We have previously used our publicly available development, SNP_TATA_Comparator [219], and manually analyzed 15,080 SNPs within 1585 proximal promoters, each 70 bp in length, located upstream of the transcription start sites of protein-coding transcripts from 534 human genes (for review, see [220]). The number of SNPs within these promoters varied from one to 64 (9.51 ± 0.21; mean ± SEM), with the 95% confidence interval being between 9 and 10; therefore, the existence of only one SNP rs34166473 within the examined region of the human *FCGR2B* gene promoter seems to suggest a statistically significant loss of SNPs (*p* < 0.01, binomial distribution). It is generally recognized that the biological function of any genomic region containing such a small number of SNPs is of vital importance, and so it is preserved by natural selection [221]. This observation is consistent with the neutrality of this single SNP rs34166473 within the human *FCGR2B* gene promoter in question, the neutrality having been confirmed with the use of our previously developed publicly available web service SNP_TATA_Comparator [219] (see Figure S1).

### 3.4. The Use of Tame and Aggressive Rats as Belyaev’s Laboratory-Animal-Based Domestication Model can Represent an Adequate Domesticated-Animal-Based Model of Human ARDs

In searching for ARD molecular markers, we used tame and aggressive rats as Belyaev’s laboratory-animal-based domestication model [69,70,71,72], because we have already found molecular markers for hypertension (incidentally, this being an ARD) and potential molecular markers for neoteny (this can reverse ARDs [73]), which correspond to the hippocampal [74] and midbrain tegmental [68] transcriptomes of these rat strains. As can be seen from Table 9 and Table 10, within tissues of the domesticated compared to wild animals there is a statistically significant excess of immunoregulators homologous to rat Fcgr2b according to two independent criteria, Pearson’s χ^2^ test (χ^2^ = 7.14; *p* < 0.01) and Fisher’s exact test (*p* < 0.05). This implies that domesticated animals are susceptible to ARDs, while their wild conspecifics are not. This result is consistent with the findings reported by Fallahshahroudi and co-workers [201] that, in the wild, natural selection on animals seeks to eliminate affected individuals, while artificial animal selection during domestication does not, as it serves human needs (for example, broilers are the cheapest poultry meat, no matter whether they are susceptible or resistant to ARDs [30,34]). This is in line with our whole-genome observations suggesting that the TATA-binding protein binding sites within the gene promoters in domesticated animals have significantly more candidate SNP markers for rheumatoid polyarthritis [222] and reproductive disorders [223,224], both being ARDs, compared to their wild conspecifics. Overall, we can conclude that Belyaev’s model of domestication using tame and aggressive rats as laboratory animals seems to be applicable as one of the adequate animal-based human ARD models.

### 3.5. Our Focus on the PAG of Tame and Aggressive Rats Is Adequate for the Search for the ARD-Linked Rat DEGs

In this work, we focused on the PAG of the tame and aggressive rats because the activity of this brain structure contributes to elevated pain tolerance with age [77]. Our interest was to find ARD-linked rat DEGs such that, if their human homologs were taken into account, we could help reduce the suffering of patients with such diseases (see Section 2.1). Recently, a meta-analysis of the microarray datasets GSE24982, GSE63442, and GSE63651 (from the Gene Expression Omnibus (GEO) database [225]) identified the rat *Fcgr2b* gene as one of the six most likely hub genes responsible for neuropathic pain and aging [226], just as we found the same rat *Fcgr2b* gene to be a molecular marker for ARDs (see Table 2, Table 6, Table 7, Table 9, and Table 10). Apparently, this independent microarray meta-analysis result [226] favors the adequacy of our focus on the PAG of the tame versus aggressive rats in the search for ARD-linked rat DEGs.

Briefly, we used the PAG of the tame and aggressive rats and found rat *Fcgr2b* overexpression to be a molecular marker for elevated neuropathic pain tolerance in ARDs.

### 3.6. In Silico Associative Gene Network of Human Immunoregulators Homologous to the Rat Fcgr2b Gene Identified as a Molecular Marker for Pain Tolerance in ARDs

**Figure 5:**
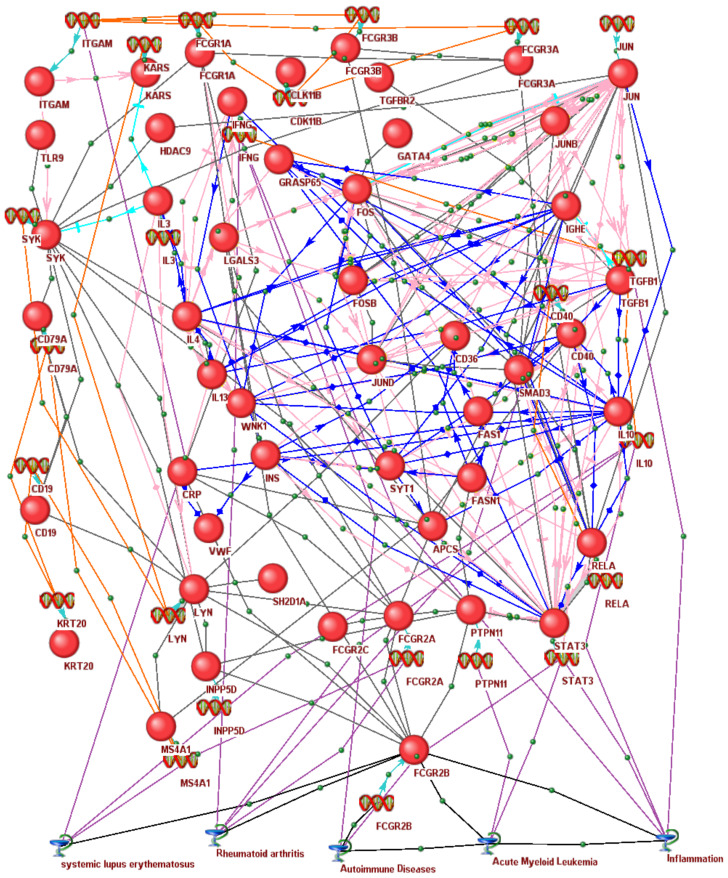
The *FCGR2B*-related associative gene network as a data-mining summary of molecular genetic research articles and databases. The network was built using the automated mode of our publicly available web service ANDSystem [227] with “Human, FCGR2B, gene, protein” as input. *Legend:* Nodes: red circles, proteins; icons “double DNA helix”, genes; “Bowl of Hygieia” symbols, diseases. Edges: turquoise sharp and blunt arrows, expression and degradation, respectively; pink sharp and blunt arrows, up- and downregulation, respectively; blue arrows, transport regulation; purple, orange, and black lines: involvement, coexpression, and association, respectively. CRP and APCS, pentraxin 1 and 2, respectively; CD19, CD36, CD40, and CD79A, proteins CD19, CD36, CD40, and CD79a, respectively; CDK11B, cyclin-dependent kinase 11B; CRP, C-reactive protein; FAS, Fas cell surface death receptor; FASN, fatty acid synthase; FCGR1A, FCGR2A, FCGR2C, FCGR3A, and FCGR3B, Fcγ receptors Ia, IIa, IIc, IIIa, and IIIb, respectively; FOS, FOSB, JUN, JUNB, and JUND, transcription factor AP-1 subunits Fos, FosB, Jun, JunB, and JunD, respectively; GATA4, GATA-binding protein 4; GRASP65, Golgi reassembly-stacking protein 1; HDAC9, histone deacetylase 9; IFNG, interferon γ; IL3, IL4, IL10, and IL13, interleukins 3, 4, 10, and 13, respectively; INPP5D, inositol polyphosphate-5-phosphatase D; INS, insulin; ITGAM, integrin subunit αM; KARS1, lysyl-tRNA synthetase 1; KRT20, keratin 20; LGALS3, galectin 3; LYN, tyrosine-protein kinase Lyn; MS4A1, membrane-spanning 4-domains subfamily A member 1; PTPN11, protein tyrosine phosphatase non-receptor type 11; RELA, NFκB subunit Rela; SH2D1A, SH2 domain-containing protein 1A; SMAD3, SMAD family member 3; STAT3, signal transducer and activator of transcription 3; SYK, spleen tyrosine kinase; SYT1, synaptotagmin 1; TGFB1 and TGFBR2, transforming growth factor β1 and its receptor II TLR9 (Toll-like receptor 9); VWF, von Willebrand factor; WNK1, WNK lysine-deficient protein kinase 1.

For a more detailed discussion of this correspondence between our present findings and the independent experimental data [226], see Figure 5. It presents an *FCGR2B*-related associative gene network as a data-mining summary of both papers and databases, which we built here using the automated mode of our publicly available web service ANDSystem [227] with “Human, FCGR2B, gene, protein” as input data. First of all, there are all six human genes in the figure, *FCGR1A*, *FCGR2A*, *FCGR2B, FCGR2C*, *FCGR3A*, and *FCGR3B*, homologous to the rat *Fcgr2b* gene identified as a molecular marker for elevated neuropathic pain tolerance in ARDs. Furthermore, there are many epigenetic regulation genes (e.g., *HDAC9* for histone deacetylase 9) in line with an ortholog-based expectable age-dependent expression pattern of these immunoregulatory genes (Figure 5), which may be under stabilizing selection (Figure S1). Finally, at the bottom of this figure, are the top five diseases with the best ratings of statistical significance for the contribution of *FCGR2B* to their pathogenesis, namely: acute myeloid leukemia (*P_ADJ_* < 10^−84^), rheumatoid arthritis (*P_ADJ_* < 10^−71^), inflammation (*P_ADJ_* < 10^−69^), systemic lupus erythematosus (*P_ADJ_* < 10^−67^), and autoimmune diseases (*P_ADJ_* < 10^−60^). Two of these five diseases, systemic lupus erythematosus and autoimmune diseases, were among the same top five of 174 diseases most significantly associated with the human *FCGR2B* gene according to the human disease database MalaCards [228] (statistical significance according to the binomial distribution; *p* < 0.01).

### 3.7. Human Immunoregulatory Genes Homologous to the Rat Fcgr2b Gene Identified as a Molecular Marker for Pain Tolerance in Age-Related Diseases Are Young on the Molecular-Evolution Scale

**Figure 6:**
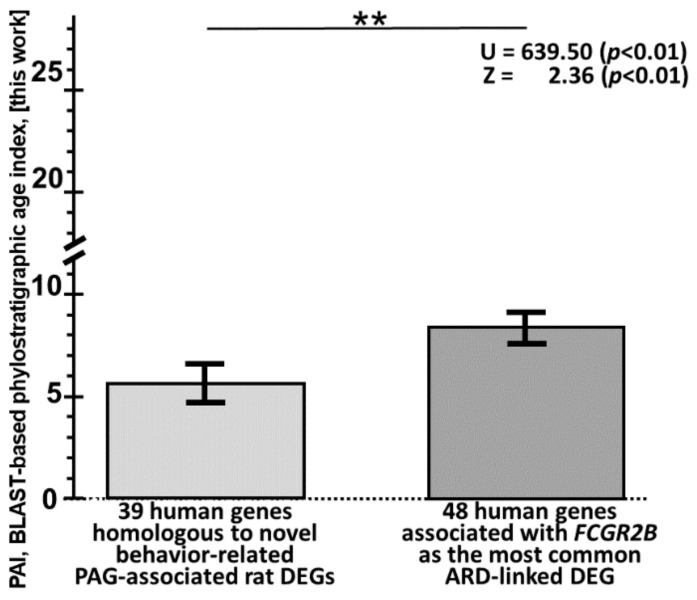
A comparison between 48 human *FCGR2B*-associated genes using the publicly available toolbox ANDSystem [227] (Figure 5) and the human genes homologous to 39 novel PAG-related rat DEGs (Table 2). Legend*:* the double asterisk (i.e., double character “**”) denotes statistical significance at *p* < 0.01 according to nonparametric Mann–Whitney *U* test and parametric Fisher’s Z-test; PAI, a gene’s phylostratigraphic age index evaluated against the BLAST-based scale [232] using the freely available web service Orthoscape [229]; BLAST-based PAI scale: 0, Cellular organisms; 1, Eukaryota; 2, Opisthokonta; 3, Metazoa; 4, Eumetazoa; 5, Bilateria; 6, Deuterostomia; 7, Chordata; 8, Craniata; 9, Vertebrata; 10, Gnathostomata; 11, Teleostomi; 12, Euteleostomi; 13, Sarcopterygii; 14, Dipnotetrapodomorpha; 15, Tetrapoda; 16, Amniota; 17, Mammalia; 18, Theria; 19, Eutheria; 20, Euarchontoglires; 21, Primates; 22, Haplorrhini; 23, Simiiformes; 24, Catarrhini; 25, Hominoidea; 26, Hominidae; 27, Homininae; and 28, Homo.

Because the human *FCGR2B* gene orthologous to the rat *Fcgr2b* gene identified as a molecular marker for elevated neuropathic pain tolerance in age-related diseases seems to be under stabilizing selection (Figure S1), we used our Orthoscape plug-in [229,230] within the Cytoscape software suite [231] and estimated BLAST-based [232] phylostratigraphic age indexes (PAIs) for (a) 39 human genes homologous to the behavior-related PAG-associated rat DEGs identified in this work (Table 2) and (b) 48 human *FCGR2B*-associated genes (Figure 5). The results are in the top and bottom parts of Table S4. As can be seen from the top part of this table, *FCGR2B* is one of the two youngest behavior-related human genes. Furthermore, the top ten youngest genes among all 48 human genes in its bottom part contain all six human *FCGR1A*, *FCGR2A*, *FCGR2B*, *FCGR2C*, *FCGR3A*, and *FCGR3B* genes, which is a statistically significant event according to the binomial distribution (*p* < 0.0001). Finally, as can be seen from Figure 6, the 48 *FCGR2B*-associated human genes examined are significantly younger than the 39 behavior-related human genes, according to the nonparametric Mann–Whitney *U* test (*p* < 0.01) and the parametric Fisher’s Z-test (*p* < 0.01).

### 3.8. Human Immunoregulatory Genes Homologous to the Rat Fcgr2b Gene Identified as a Molecular Marker for Pain Tolerance in ARDs can Be Permanently Available in Any Tissue of Anyone’s Organism

Human immunoregulatory genes homologous to the rat *Fcgr2b* gene that we have identified as a molecular marker for elevated neuropathic pain tolerance in ARDs are expressed at increased rates mostly in conventional, monocyte-derived, and plasmacytoid dendritic cells as well as macrophages [233], which occur in most tissues, where they are critical to tissue homeostasis [234]. According to the GeneCard database [235], a large number of microarray, RNA-Seq, and proteomics experiments have detected molecular products expressed from the human genes *FCGR1A*, *FCGR2A*, *FCGR2B*, *FCGR2C, FCGR3A*, and *FCGR3B* in the majority of the human tissues studied. Because disruptions in the cellular-senescence-associated tissue homeostasis compromise the correct activation of immune responses to pathogens and cancer cells [236,237], these human immunoregulatory genes can be permanently overexpressed in any tissue of anyone’s organism as molecular markers for elevated neuropathic pain tolerance in ARDs (see Table 7).

## 4. Materials and Methods

### 4.1. Animals

The animals used were adult male gray rats (*R. norvegicus*) artificially bred for over 90 generations for either aggressive or tame behavior towards humans as two outbred strains. The rats were kept under standard conditions of the Conventional Animal Facility at the ICG SB RAS (Novosibirsk, Russia), as described elsewhere [72,79,238]. The total number of rats was 22 (11 tame and 11 aggressive), each 4 months old and weighing 250–270 g, all from unrelated litters. For the gene expression analysis, all the rats were decapitated. PAG samples were excised according to a handbook technique [239], flash-frozen in liquid nitrogen, and stored at −70 °C until use. This work was conducted in line with the guidelines of the Declaration of Helsinki, of Directive 2010/63/EU of the European Parliament, and of the European Council resolution of 22 September 2010. The research protocol was approved by the Interinstitutional Commission on Bioethics at the ICG SB RAS, Novosibirsk, Russia (Approval documentation No. 8 dated 19 March 2012).

### 4.2. RNA-Seq

Total RNA was isolated from ~100 mg of the PAG tissue samples of tame (*n* = 3) and aggressive (*n* = 3) rats using the TRIzol™ reagent (Invitrogen, Carlsbad, CA, USA). The quality of the total-RNA samples was measured on a Bioanalyzer 2100 (Agilent, Santa-Clara, CA, USA). Samples with optimal RNA integrity numbers (RINs) were selected for further analysis. Furthermore, the total RNA was analyzed quantitatively on an Invitrogen Qubit™ 2.0 fluorometer (Invitrogen). Different RNA types were separated with the mirVana™ Kit (Thermo Fisher Scientific, Waltham, MA, USA). The Dynabeads mRNA Purification Kit (Invitrogen) was used to prepare highly purified mRNA from 5 μg of the RNA fraction depleted of small RNAs. Preparation of RNA-Seq libraries from 15–30 ng of an mRNA fraction was carried out with the help of the ScriptSeq™ v2 RNA-Seq Library Preparation Kit (epicenter^®^, Madison, WI, USA). The quality of the libraries obtained was examined on a Bioanalyzer 2100. After normalization, barcoded libraries were pooled and handed over to the Multi-Access Center of Genomic Research (ICG SB RAS, Novosibirsk, Russia) for sequencing on an Illumina NextSeq 550 instrument in a NextSeq^®^ 500/550 High Output Kit v2 cassette (75 cycles) under the assumption of a direct read of 75 nucleotides, with at least 40 million reads.

### 4.3. Mapping of RNA Sequences to the R. norvegicus Reference Genome

The primary raw Fastq files were examined using a quality-control tool for high-throughput sequencing data (FastQC; https://www.bioinformatics.babraham.ac.uk/projects/fastqc; accessed on 19 December 2018). Next, we improved the quality of the raw reads using the Trimmomatic tool [240] in a step-by-step manner as follows: (i) discarded a base from either the start or end position if the quality was low; (ii) trimmed bases with a sliding-window method, and (iii) eliminated any remaining reads that were less than 36 bases in length. After that, we aligned the trimmed reads to the *R*. *norvegicus* reference genome (RGSC Rnor_6.0, UCSC version Rn6, July 2014 assembly) using the TopHat2 toolbox [241]. Next, we reformatted these alignments into sorted BAM files in SAMtools version 1.4 [242]. Then we assigned the reads in question to these genes using the htseq-count tool from the preprocessing software HTSeq v.0.7.2 [243] along with gtf files containing the coordinates of the rat genes according to Rnor_6.0 and an indexed SAM file. Finally, we used DESeq2 [244] via the web service IRIS (http://bmbl.sdstate.edu/IRIS/; accessed on 16 January 2020), rated differential expression levels of the rat genes, and, to minimize false-positive error rates, applied Fisher’s Z-test [245] with the Benjamini correction for multiple comparisons as well as discarded all hypothetical, tentative, predicted, uncharacterized, and non-protein-coding genes.

### 4.4. qPCR

To examine independently and selectively the novel tame versus aggressive rat PAG DEGs identified here (Table 2), we performed a qPCR control assay on the total RNA taken from the remaining samples of the PAG of tame (*n* = 8) and aggressive (*n* = 8) rats. First, we isolated total RNA using TRIzol™, purified it on Agencourt RNAClean XP Kit magnetic beads (Beckman, #A63987), and quantified it using a Qubit™ 2.0 fluorometer (Invitrogen/Life Technologies) and a Qubit™ RNA High-Sensitivity Assay Kit (Invitrogen, cat. # Q32852). Next, we synthesized cDNA using the Reverse Transcription Kit (Syntol, #OT-1). Finally, we designed oligonucleotide primers for qPCR using the web service PrimerBLAST [246] (Table 11).

After that, we conducted qPCR on a LightCycler^®^ 96 (Roche, Basel, Basel-Stadt, Switzerland) with the EVA Green I Kit in three technical replicates. We estimated qPCR efficiency using serial cDNA dilutions (standards). Following the recommendations set out in the MIQE guidelines [84], we analyzed three reference genes at once: *B2m* (β-2-microglobulin) [81], *Hprt1* (hypoxanthine phosphoribosyltransferase 1) [82], and *Rpl30* (ribosomal protein L30) [83].

### 4.5. DEGs

We have analyzed all publicly available independent experimental RNA-Seq datasets of the transcriptomes of tissues from ARD-susceptible versus ARD-resistant subjects (humans [33,36,37,38,39,40,41,42,43] and animals [30,32,35,44,45,46,47,48,49,50,51,52,53,54,55,56,57,58,59,60,61,62,63,64]) and the transcriptomes of tissues from domestic versus wild animals [68,74,204,205,206,207,208,209,210,211].

### 4.6. Human Genes

We analyzed the 39 human genes that are orthologous to the 39 PAG-related DEGs of the tame and aggressive rats (Table 2). With reliance on PubMed [75], we gathered clinical information about whether downregulation or upregulation of each of these 39 human genes can alleviate or aggravate ARDs (Table 9 and Table S3).

### 4.7. DNA Sequences

For in silico analysis of the human genes encoding the novel candidate molecular markers for ARDs, we retrieved the DNA sequences and SNPs of their 70 bp proximal promoters from the Ensembl database [247] and the dbSNP database [218], respectively, using the UCSC Genome Browser (reference human genome assembly GRCh38/hg38) [248] in both dialog mode and automated mode using the Bioperl toolkit [249] (Figure S1).

### 4.8. In Silico Analysis of DNA Sequences

We analyzed SNPs within DNA sequences using our previously published publicly available web service SNP_TATA_Comparator [219], which implements our bioinformatic model of three-step binding of TATA-binding protein (TBP) to a given 70 bp proximal promoter of the human gene under study, as detailed in the Supplementary Materials (Section S1 “Supplementary methods for DNA sequence analysis”) [250,251,252,253,254,255,256,257,258,259,260,261,262] and additionally illustrated in Figure S1.

### 4.9. A Knowledge Base for Domestic Animal DEGs Whose Human Orthologs Can Affect ARD Severity

In files with the flat Excel-compatible textual format, we have documented all proposed associations between the domestic and wild animal DEGs homologous to the 39 novel DEGs that we found in the PAG of the tame and aggressive rats. We have also documented how downregulation or upregulation of the human genes homologous to these PAG-related rat DEGs can affect ARD severity. Finally, we have added our current findings to our previously published public PetDEGsDB knowledge base, its new build being freely available at www.sysbio.ru/domestic-wild (accessed on 14 December 2022) in the MariaDB 10.2.12 database management system (MariaDB Corp AB, Espoo, Finland).

### 4.10. Data Mining of Literature Sources and Databases Publicly Available on the Internet

We conducted data mining using the automated mode of our previously published freely available web service ANDSystem [227], with “Human, FCGR2B, gene, protein” as input keywords, with all the other parameters set at their default values.

### 4.11. In Silico Estimation of the BLAST-Based PAIs of a Given Human Gene

We estimated the BLAST-based [232] PAIs for a given human gene whose NCBI Entrez gene number served as input for our Orthoscape plug-in [229,230] within the Cytoscape software suite [231]. The output was the most recent common ancestor of all the animal species whose DNA sequence of this gene is already known. The following evolutionary rank scale was used: 0, Cellular organisms; 1, Eukaryota; 2, Opisthokonta; 3, Metazoa; 4, Eumetazoa; 5, Bilateria; 6, Deuterostomia; 7, Chordata; 8, Craniata; 9, Vertebrata; 10, Gnathostomata; 11, Teleostomi; 12, Euteleostomi; 13, Sarcopterygii; 14, Dipnotetrapodomorpha; 15, Tetrapoda; 16, Amniota; 17, Mammalia; 18, Theria; 19, Eutheria; 20, Euarchontoglires; 21, Primates; 22, Haplorrhini; 23, Simiiformes; 24, Catarrhini; 25, Hominoidea; 26, Hominidae; 27, Homininae; and 28, Homo.

### 4.12. Statistical Analysis

We performed the Mann–Whitney *U* test, Fisher’s Z-test, Pearson’s linear correlation test, Goodman–Kruskal generalized correlation test, Spearman’s and Kendall’s rank correlation tests, Pearson’s χ^2^ test, Fisher’s exact test, and the binomial-distribution analysis using appropriate options in the standard software STATISTICA (Statsoft^TM^). Furthermore, using the PAST4.04 software package [85], we conducted a principal component analysis in the bootstrap refinement mode via its mode selection path “Multivariate”→”Ordination”→“Principal Components (PCA)”→“Correlation”→“Bootstrap.”

## 5. Conclusions

First, we have profiled the PAG transcriptome in three tame adult male rats versus three aggressive conspecifics, all animals being unrelated, and made the primary raw reads publicly available (NCBI SRA database ID: PRJNA668014) [80]. With the use of this transcriptome, we have found 39 PAG-related DEGs whose statistical significance (*P_ADJ_* < 0.05, Fisher’s Z-test with the Benjamini correction for multiple comparisons) was acceptable (Table 2). We have selectively verified these DEGs with independent experimental analyses (qPCR) of eight other tame and eight other aggressive adult male rats from unrelated litters of the same two outbred strains (Table 3 and Figure 1).

Secondly, we have found that Fcγ receptor IIb overexpression is a statistically significant molecular marker for ARDs. To come to this conclusion, we compared 39 novel DEGs in the PAG of tame and aggressive rats to their known homologs associated with ARDs in animals and humans, using correlation and principal component analysis, as well as Bonferroni’s correction for multiple comparisons.

Finally, we propose the human immunoregulatory genes *FCGR1A*, *FCGR2A*, *FCGR2B*, *FCGR2C, FCGR3A*, and *FCGR3B* homologous to the rat *Fcgr2b* gene as theranostic molecular markers for age-related diseases.

## Figures and Tables

**Table 1:** A summary of searches for DEGs in the PAG transcriptomes of three tame adult male rats (*R. norvegicus*) and three aggressive adult male rats, all animals being unrelated.

Group	Tame vs. Aggressive Rats
Total number of sequence reads (NCBI SRA ID: PRJNA668014)	210,128,758
Reads mapped to reference rat genome RGSC Rnor_6.0, UCSC Rn6, July 2014 (%)	177,608,837 (84.5%)
Expressed genes identified	14,039
Statistically significant DEGs (*P_ADJ_* < 0.05, Fisher’s Z-test with the Benjamini correction)	39

**Table 2:** Statistically significant DEGs newly identified in the PAG of the tame and aggressive adult male rats.

#	Rat Gene Name	*Symbol*	log_2_	*P_ADJ_*
1	Amylase α1A	*Amy1a*	1.97	<0.05
2	Aldehyde oxidase 1	*Aox1*	1.82	<0.05
3	Achaete-scute family bHLH transcription factor 3	*Ascl3*	2.74	<10^−4^
4	BTG3-associated nuclear protein	*Banp*	−0.64	<0.05
5	Bradykinin receptor B2	*Bdkrb2*	1.62	<0.05
6	Cocaine- and amphetamine-regulated transcript prepropeptide	*Cartpt*	3.53	<10^−7^
7	Cytochrome P450, family 2, subfamily j, polypeptide 10	*Cyp2j10*	1.10	<10^−2^
8	Defensin β17	*Defb17*	6.96	<10^−2^
9	Empty spiracles homeobox 2	*Emx2*	1.45	<10^−2^
10	FAT atypical cadherin 2	*Fat2*	−4.96	<0.05
11	Fc γ receptor IIb	*Fcgr2b*	2.02	<0.05
12	AP-1 transcription factor subunit FosB proto-oncogene	*Fosb*	−1.85	<10^−3^
13	Glycerol-3-phosphate dehydrogenase 1	*Gpd1*	−0.99	<0.05
14	Hemoglobin, β adult major chain	*Hbb-b1*	−5.90	<10^−5^
15	Heat shock protein family A (Hsp70) member 1B	*Hspa1b*	−2.14	<10^−7^
16	Integral membrane protein 2A	*Itm2a*	0.76	<10^−2^
17	Keratin 2	*Krt2*	−1.85	<10^−6^
18	MORN repeat-containing 1	*Morn1*	0.75	<0.05
19	Myomesin 2	*Myom2*	−1.63	<0.05
20	Nuclear transcription X-box-binding like 1 factor	*Nfxl1*	0.69	<10^−2^
21	Neuromedin B	*Nmb*	−3.27	<10^−4^
22	Nicotinamide nucleotide adenylyltransferase 1	*Nmnat1*	0.94	<10^−4^
23	Purkinje cell protein 2	*Pcp2*	−5.91	<10^−2^
24	Protein disulfide isomerase family A member 4	*Pdia4*	−0.59	<10^−2^
25	Prodynorphin	*Pdyn*	−1.02	<10^−2^
26	Phospholipase A2, group IIC	*Pla2g2c*	−1.60	<10^−4^
27	Procollagen-lysine,2-oxoglutarate 5-dioxygenase 1	*Plod1*	−0.85	<10^−2^
28	Phosphotriesterase-related protein	*Pter*	1.27	<10^−2^
29	Glycogen phosphorylase L	*Pygl*	−0.95	<0.05
30	RNA-binding motif protein 3	*Rbm3*	1.22	<10^−4^
31	Retinol saturase	*Retsat*	−1.01	<0.05
32	Rho-related BTB domain-containing protein 3	*Rhobtb3*	0.85	<0.05
33	Relaxin 3	*Rln3*	−3.73	<10^−3^
34	Sciellin	*Scel*	1.24	<0.05
35	Schlafen family member 13	*Slfn13*	1.33	<0.05
36	Serine peptidase inhibitor, Kunitz type 1	*Spint1*	−1.16	<10^−3^
37	Troponin T type 1 (skeletal, slow)	*Tnnt1*	2.80	<0.05
38	Urocortin	*Ucn*	2.61	<0.05
39	WD repeat and SOCS box-containing protein 1	*Wsb1*	0.95	<0.05

Note: Hereinafter, log_2_: the log_2_-transformed fold change (i.e., the ratio of the expression level of a given gene in tame rats to that in aggressive rats); *p* and *P_ADJ_*: statistical significance according to Fisher’s Z-test and the Benjamini correction for multiple comparisons.

**Table 3:** qPCR data on a selection of DEGs from the PAG of 16 additional unrelated adult male rats, eight tame and eight aggressive.

Design		Glove Test [79] and qPCR Data on Gene Expression [This Work]								
**Rat**	**Set**	No. 1	2	3	4	5	6	7	8	
**Glove** **test**	**A**	−3.5	−3.5	−3.5	−3.5	−3.5	−3.5	−3.5	−3.5	
	**T**	3.5	3.5	3.5	3.5	3.5	3.5	3.5	3.5	
***DEG***	**Set**	**Relative expression with respect to three reference genes, qPCR, M_0_ ± SEM**								**TOTAL**
***Ascl3***	**A**	0.10 ± 0.05	0.26 ± 0.01	0.64 ± 0138	0.12 ± 0.03	0.24 ± 0.07	0.10 ± 0.03	0.13 ± 0.005	0.15 ± 0.02	0.22 ± 0.06
	**T**	1.63 ± 0.03	1.66 ± 0.16	1.86 ± 0.15	4.30 ± 0.06	3.37 ± 0.18	4.91 ± 0.13	4.14 ± 0.90	4.95 ± 0.86	3.35 ± 0.51
***Defb17***	**A**	0.007 ± 0.005	ND	ND	ND	0.005 ± 0.005	0.005 ± 0.005	0.005 ± 0.005	0.005 ± 0.005	0.005 ± 0.005
	**T**	3.17 ± 0.30	3.06 ± 0.16	2.91 ± 0.11	2.74 ± 0.22	1.62 ± 0.17	3.42 ± 0.51	1.67 ± 0.07	3.77 ± 0.07	2.08 ± 0.27

Note: Datasets: A, aggressive rats; T, tame rats. qPCR data: “M_0_ ± SEM” denotes the mean ± standard error of the mean for three technical replicates for each rat; ND, not detected.

**Table 4:** Found in PubMed [75]: DEGs in human ARD models based on animal data.

#	Species	ARD Susceptibility	ARD Resistance	Tissue	N_DEG_	Ref.
1	rat	OXYS: spurt aging, 20-day-old	Wistar, 20-day-old	hippocampus	46	[44]
2	rat	OXYS: spurt aging, 5-month-old	Wistar, 5-month-old	hippocampus	28	[44]
3	rat	OXYS: spurt aging, 18-month-old	Wistar, 18-month-old	hippocampus	85	[44]
4	rat	OXYS: spurt aging, 18-month-old	Wistar, 18-month-old	prefrontal cortex	59	[45]
5	rat	OXYS: spurt aging, 18-month-old	Wistar, 18-month-old	retina	77	[46]
6	rat	ISIAH (hypertensive aged vessels)	WAG (norm)	brain stem	206	[47]
7	rat	ISIAH (hypertensive aged vessels)	WAG (norm)	hypothalamus	137	[48]
8	rat	ISIAH (hypertensive aged vessels)	WAG (norm)	renal medulla	882	[49]
9	rat	ISIAH (hypertensive aged vessels)	WAG (norm)	renal cortex	309	[50]
10	rat	ISIAH (hypertensive aged vessels)	WAG (norm)	adrenal gland	1020	[51]
11	rat	SHR (hypertensive aged vessels)	Wistar (norm)	brain pericytes	21	[52]
12	rat	20-passage-old	5-passage-old	MSC(BM)	9167	[35]
13	rat	5-passage-old	5-passage-old, Aspirin	MSC(BM)	1220	[35]
14	rat	20-passage-old	20-passage-old, Aspirin	MSC(BM)	446	[35]
15	mice	11-month-old, bone fragility	2-month-old, norm	bone	1011	[55]
16	mice	23-month-old, bone fragility	2-month-old, norm	bone	1151	[55]
17	mice	30-month-old, bone fragility	2-month-old, norm	bone	3725	[55]
18	mice	30-month-old, bone fragility	2-month-old, norm	kidney	43	[56]
19	mice	27-month-old, males, renal fibrosis	2-month-old, males	kidney	349	[56]
20	mice	27-month-old, females, renal fibrosis	2-month-old, females	kidney	100	[56]
21	mice	24-month-old, renal fibrosis	3-month-old	kidney	599	[57]
22	mice	PolG D257A, cardiac disorder	wild-typed norm	heart right ventricle	402	[58]
23	mice	20-month-old, parabiont, 8 weeks	6-month-old, parabiont, 8 weeks	aortic arch	23	[59]
24	mice	8 h:8 h biorhythm (autistic-like)	12 h/12 h biorhythm norm	hippocampus	158	[60]
25	mice	wild-type, 20-week-old, 60% diet	wild-type, 20-week-old, ad libitum	skeletal muscle	1178	[61]
26	mice	wild-type, 80-week-old, 60% diet	wild-type, 80-week-old, ad libitum	skeletal muscle	747	[61]
27	mice	*Sirt1*-KO, 20-week-old, 60% diet	*Sirt1*-KO, 20-week-old, ad libitum	skeletal muscle	2323	[61]
28	mice	*Sirt1*-KI, 20-week-old, 60% diet	*Sirt1*-KI, 20-week-old, ad libitum	skeletal muscle	1919	[61]
29	mice	*Sirt1*-KO, 80-week-old, 60% diet	*Sirt1*-KO, 80-week-old, ad libitum	skeletal muscle	721	[61]
30	mice	*Sirt1*-KI, 80-week-old, 60% diet	*Sirt1*-KI, 80-week-old, ad libitum	skeletal muscle	2641	[61]
31	mice	*Sirt1*-KO, 80-week-old*,* ad libitum	wild-type, 80-week-old, ad libitum	skeletal muscle	1976	[61]
32	mice	wild-type, 80-week-old, ad libitum	*Sirt1*-KI, 80-week-old, ad libitum	skeletal muscle	445	[61]
33	mice	*Sirt1*-KO, 20-week-old, ad libitum	wild-type, 20-week-old, ad libitum	skeletal muscle	1152	[61]
34	mice	wild-type, 20-week-old, ad libitum	*Sirt1*-KI, 20-week-old, ad libitum	skeletal muscle	135	[61]
35	mice	BPH/2J, hypertensive, aged vessels	BPN/3J, norm	kidney	883	[62]
36	rabbit	under Goldblatt 2-kidney 1-clip	under sham-operated control	prefrontal cortex	229	[63]
37	chicken	1.2% Ca diet: worst health	0.8% Ca diet: best health	kidney	92	[30]
38	chicken	1% Ca diet, health threshold	0.8% Ca diet: best health	kidney	83	[30]
39	chicken	1.2% Ca diet: worst health	1% Ca diet, health threshold	kidney	64	[30]
40	chicken	456-day-old, subfertility	224-day-old, fertility peak	ovary	259	[34]
41	chicken	469-day-old, hunger, infertility	456-day-old, subfertility	ovary	926	[34]
42	chicken	469-day-old, hunger, infertility	527-day-old, fasting-diet, fertility	ovary	698	[34]
43	fruit fly	10-day-old, Alzheimer disease-like	0-day-old, just post-eclosion	head	99	[64]
Σ	5 species	43 human age-related disease models using animals	17 tissues	37,834	22 Refs	

Note: N_DEG_: the number of DEGs; OXIS, Wistar, BPH/2J, and BPH/3J: laboratory animal strains used; MSC(BM): bone-marrow-derived mesenchymal stromal cells; aspirin and fasting: rejuvenators; *PolG*: DNA polymerase γ, catalytic subunit; Sirt1: sirtuin 1; KO and KI: knock-out and knock-in, respectively.

**Table 5:** DEGs in the binary “susceptibility versus resistance” models of human ARDs (PubMed data, [75]).

#	ARD Susceptibility	ARD Resistance	Tissue	N_DEG_	Ref.
1	renal medullary hypertension	norm	renal medulla	13	[36]
2	pulmonary arterial hypertension	norm	blood	14	[37]
3	pulmonary arterial hypertension	norm	lung	118	[38]
4	fibrosis in pulmonary hypertension	norm	lung	3516	[39]
5	idiopathic pulmonary hypertension	norm	lung	5639	[39]
6	nephrosclerosis as kidney aging	norm	kidney	16	[40]
7	atrial fibrillation as heart aging	norm	auricle tissue	300	[41]
8	myocardial ischemia as aged heart	norm	peripheral blood	1524	[41]
9	ALS as aged motoneurons	norm	small extracellular vesicles	402	[42]
10	FTD as cognitive ageing	norm	small extracellular vesicles	164	[42]
11	ALS as aged motor neurons	norm	large extracellular vesicles	62	[42]
12	FTD as cognitive ageing	norm	large extracellular vesicles	55	[42]
13	before exercise training	after exercise training	vastus externus	170	[33]
14	LPS-stimulated atherogenesis	ARID5B-KO as atheroprotection	THP1 monocytes	2542	[43]
∑	14 binary models of human ARDs		10 tissues	14,535	9 Refs

Note: See the footnote to Table 4. Diseases: ALS, amyotrophic lateral sclerosis; FTD, frontotemporal dementia. LPS, lipopolysaccharide; *ARID5B*, the AT-rich interaction domain 5B gene.

**Table 6:** Searching for ARD molecular genetic markers among human genes homologous to 39 novel PAG-related DEGs of the tame and aggressive rats. The number of these homologous DEGs linked to ARD susceptibility and ARD resistance in humans and animals is taken into account.

Rat Gene		Total Number of DEGs		Binomial Distribution		Rat Gene		Total Number of DEGs		Binomial Distribution	
#	*Symbol*	N_PC1_: Matching Signs	N_PC2_: Opposite Signs	*p*	*P*_ADJ_	#	*Symbol*	N_PC1_: Matching Signs	N_PC2_: Opposite Signs	*P*	*P*_ADJ_
i	ii	iii	iv	v	vi	i	ii	iii	iv	V	vi
1	*Amy1a*	7	3	0.17	1.00	21	*Nmb*	1	4	0.19	1.00
2	*Aox1*	8	5	0.29	1.00	22	*Nmnat1*	3	7	0.17	1.00
3	*Ascl3*	3	1	0.31	1.00	23	*Pcp2*	5	1	0.11	1.00
4	*Banp*	8	3	0.11	1.00	24	*Pdia4*	10	6	0.23	1.00
5	*Bdkrb2*	3	4	0.50	1.00	25	*Pdyn*	0	0	ND	ND
6	*Cartpt*	1	1	0.50	1.00	26	*Pla2g2c*	40	32	0.20	1.00
7	*Cyp2j10*	6	5	0.50	1.00	27	*Plod1*	16	3	0.002	0.08
8	*Defb17*	10	1	0.006	0.23	28	*Pter*	2	3	0.50	1.00
9	*Emx2*	1	0	0.50	1.00	29	*Pygl*	4	9	0.13	1.00
10	*Fat2*	15	5	0.021	0.81	30	*Rbm3*	27	12	0.012	0.46
11	*Fcgr2b*	17	1	0.00007	0.005	31	*Retsat*	4	8	0.19	1.00
12	*Fosb*	7	13	0.13	1.00	32	*Rhobtb3*	8	18	0.038	1.00
13	*Gpd1*	7	4	0.27	1.00	33	*Rln3*	0	2	0.25	1.00
14	*Hbb-b1*	10	18	0.09	1.00	34	*Scel*	2	3	0.50	1.00
15	*Hspa1b*	28	21	0.20	1.00	35	*Slfn13*	16	7	0.05	1.00
16	*Itm2a*	4	17	0.004	0.14	36	*Spint1*	2	4	0.34	1.00
17	*Krt2*	27	21	0.24	1.00	37	*Tnnt1*	5	9	0.21	1.00
18	*Morn1*	12	2	0.006	0.25	38	*Ucn*	2	0	0.25	1.00
19	*Myom2*	11	8	0.32	1.00	39	*Wsb1*	3	3	0.65	1.00
20	*Nfxl1*	3	5	0.36	1.00						

Note: *p* and *P_ADJ_*: significance according to the binomial distribution without or with Bonferroni’s correction for multiple comparisons, respectively; ND: not detected; underlined is the only common statistically significant ARD molecular marker found in this work: *Fcgr2b*. Matching signs: the same direction of expression change; opposite signs: opposite directions of expression change.

**Table 7:** Statistically significant data on upregulation of Fcγ-receptor-IIb-related DEGs linked to ARD susceptibility and ARD resistance in the subject species.

#	Species	Age-Related Disease Susceptibility	Age-Related Disease Resistance	Tissue	*DEG*	log_2_	*P_ADJ_*	Ref.
1	rat	20-passage-old	5-passage-old	MSC(BM)	*Fcgr2b*	6.24	10^−3^	[35]
2	rat	20-passage-old	5-passage-old	MSC(BM)	*Fcgr2a*	3.99	10^−80^	[35]
3	rat	20-passage-old	5-passage-old	MSC(BM)	*Fcgr3a*	2.86	10^−44^	[35]
4	rat	20-passage-old	20-passage-old, Aspirin	MSC(BM)	*Fcgr2b*	1.00	10^−6^	[35]
5	rat	ISIAH, hypertensive aged vessels	WAG (norm)	adrenal gland	*Fcgr1a*	0.76	0.05	[51]
6	rat	ISIAH, hypertensive aged vessels	WAG (norm)	adrenal gland	*Fcgr3a*	0.67	0.05	[51]
7	mice	30-month-old, bone fragility	2-month-old, norm	Bone	*Fcgr2b*	1.11	10^−3^	[55]
8	mice	30-month-old, bone fragility	2-month-old, norm	Bone	*Fcgr1*	1.12	10^−2^	[55]
9	mice	30-month-old, bone fragility	2-month-old, norm	Bone	*Fcgr3*	0.92	10^−2^	[55]
10	mice	30-month-old, bone fragility	2-month-old, norm	Bone	*Fcgr3*	1.39	0.05	[56]
11	mice	PolG D257A, cardiac disorder	wild-type, norm	heart right ventricle	*Fcgr4*	1.91	10^−2^	[58]
12	human	fibrosis in pulmonary hypertension	norm	Lung	*FCGR1B*	−1.30	0.05	[39]
13	human	idiopathic pulmonary hypertension	norm	Lung	*FCGR2A*	0.27	0.05	[39]
14	human	idiopathic pulmonary hypertension	norm	Lung	*FCGR3A*	0.22	0.05	[39]
15	human	LPS-stimulated atherogenesis	ARID5B-KO as atheroprotection	THP1 monocytes	*FCGR1A*	0.41	10^−4^	[43]
16	human	LPS-stimulated atherogenesis	ARID5B-KO as atheroprotection	THP1 monocytes	*FCGR1B*	0.55	10^−4^	[43]
17	human	LPS-stimulated atherogenesis	ARID5B-KO as atheroprotection	THP1 monocytes	*FCGR1C*	0.30	10^−2^	[43]
18	human	LPS-stimulated atherogenesis	ARID5B-KO as atheroprotection	THP1 monocytes	*FCGR2A*	0.49	10^−5^	[43]

*Note*: See footnotes to Table 4 and Table 5.

**Table 8:** RNA-Seq transcriptomes of domestic animals versus their wild conspecifics (PubMed data [75]).

#	Domestic Animals	Wild Animals	Tissue	N_DEG_	Ref.
1	tame rats	aggressive rats	hypothalamus	46	[204]
2	tame rats	aggressive rats	hippocampus	42	[74]
3	tame rats	aggressive rats	midbrain tegmentum	31	[68]
4	tame rats	aggressive rats	frontal cortex	20	[205]
5	guinea pigs	cavy	frontal cortex	883	[205]
6	domestic rabbits	wild rabbits	frontal cortex	17	[205]
7	domestic rabbits	wild rabbits	parietal-temporal cortex	216	[206]
8	domestic rabbits	wild rabbits	amygdala	118	[206]
9	domestic rabbits	wild rabbits	hypothalamus	43	[206]
10	domestic rabbits	wild rabbits	hippocampus	100	[206]
11	dogs	wolves	blood	450	[207]
12	dogs	wolves	frontal cortex	13	[205]
13	tame foxes	aggressive foxes	pituitary	327	[208]
14	pigs	boars	frontal cortex	30	[205]
15	pigs	boars	frontal cortex	34	[209]
16	pigs	boars	pituitary	22	[210]
17	domestic chicken	wild chicken	pituitary	474	[211]
Σ	7 domestic animal species	7 wild animal species	8 tissues	2866	10 Refs

**Table 9:** Comparison of the effects of unidirectional changes in the expression (**a**) of the human *FCGR2B* gene on ARD severity in humans and (**b**) of its animal homologs on the microevolutionary events leading to domestic and wild animals.

(a) Humans				(b) Animals						
Effect of changes in human *FCGR2B* expression on ARDs: aggravating (→) or alleviating (←)				Effect of changes in the expression of animal genes homologous to human *FCGR2B*						
deficient	effect	excessive	effect	deficient	excessive	tissue	*DEG*	log_2_	*P_ADJ_*	Refs
In South Asia and Africa, the human FCGR2B-deficient alleles have the most frequent occurrence as protectors against infection [117], susceptibility to which increases with age as immunosenescence [118]	←	*FCGR2B* has been explored using the “C-type lectin-like molecule-1”/”Fc-domain” fusion protein as a target antigen for chemotherapy against acute myeloid leukemia [119] as a cellular-senescence-related immunogenic disease [120]	→	aggressive rat	tame rat	PAG	*Fcgr2b*	2.02	0.05	[this work]
				aggressive rat	tame rat	hypothalamus	*Fcrl2*	1.12	0.05	[204]
				aggressive rat	tame rat	hypothalamus	*Fcgr3a*	2.06	10^−2^	[204]
				aggressive rat	tame rat	midbrain tegmentum	*Fcgr2b*	2.01	0.05	[68]
				aggressive fox	tame fox	pituitary	*Fcrl1*	0.43	10^−2^	[208]
				wild rabbit	domestic rabbit	parietal-temporal cortex	*Fcgr3b*	1.35	10^−2^	[206]
				guinea pig	cavy	frontal cortex	*Fcer2*	−1.36	0.05	[205]

Note: See footnotes to Table 4 and Table 5. *Fcer2*: Fc epsilon receptor II; *Fcrl1* and *Fcrl2:* Fc-receptor-like 1 and 2, respectively.

**Table 10:** Correlations between the effects of unidirectional changes in the expression (**a**) of the human *FCGR2B* gene on ARD severity in humans and (**b**) of its animal homologs on the microevolutionary events leading to domestic and wild animals.

	(a) Humans	Effect of Changes in Human *FCGR2B* Expression on ARDs		Pearson’s χ^2^ Test		Fisher’s Exact Test
(b) Animals		Alleviating (←)	Aggravating (→)	χ^2^	*p*	
**Effect of changes in the expression of animal homologs to human** *FCGR2B*	**wild**	6	1	7.14	10^−2^	0.05
	**domestic**	1	6			

**Table 11:** qPCR primers selected via the publicly available web service PrimerBLAST [246].

No.	*Gene*	Forward, 5′→3′	Reverse, 5′→3′
**Novel DEGs Identified in the PAG of Tame versus Aggressive Adult Male Rats**			
1	*Ascl3*	CCTCTGCTGCCCTTTTCCAG	ACTTGACTCGCTGCCTCTCT
2	*Defb17*	TGGTAGCTTGGACTTGAGGAAAGAA	TGCAGCAGTGTGTTCCAGGTC
**Reference genes**			
3	*B2m*	GTGTCTCAGTTCCACCCACC	TTACATGTCTCGGTCCCAGG
4	*Hprt1*	TCCCAGCGTCGTGATTAGTGA	CCTTCATGACATCTCGAGCAAG
5	*Rpl30*	CATCTTGGCGTCTGATCTTG	TCAGAGTCTGTTTGTACCCC

Note: For the DEGs subjected to this qPCR-based verification, see Table 2; reference rat genes: *B2m*, β-2-microglobulin [81]; *Hprt1*, hypoxanthine phosphoribosyltransferase 1 [82]; and *Rpl30*, ribosomal protein L30 [83].

## Data Availability

The primary RNA-Seq data obtained in this work were deposited in the NCBI SRA database (ID = PRJNA668014).

## Supplementary Materials

The following supporting information can be downloaded at: https://www.mdpi.com/article/10.3390/ijms24043996/s1.

## Abbreviations

ARD	age-related disease
DEG	differentially expressed gene
log2 value	log_2_-transformed gene expression fold change
PAG	periaqueductal gray matter
PAI	phylostratigraphic age index
PC1 (PC2)	major (minor) principal component
qPCR	quantitative polymerase chain reaction
RNA-Seq	RNA sequencing
SNP	single-nucleotide polymorphism
TBP	TATA-binding protein
WHO	World Health Organization
