# Supplementary material for: Differentially Expressed Genes and Molecular Susceptibility to Human Age-Related Diseases
Source: Int J Mol Sci. 2023 Feb 16;24(4):3996. doi: 10.3390/ijms24043996 (PMC9966505; doi:10.3390/ijms24043996)
Supplement: Supplementary file 1 [file ijms-24-03996-s001.zip › ijms-2163784-supplementary.pdf]

International Journal of
Molecular Sciences

MDPI

Supplementary Materials

Differentially Expressed Genes and Molecular Susceptibility to Human Age-Related Diseases

Svetlana Shikhevich[1], Irina Chadaeva[1], Bato Khandaev[1,2], Rimma Kozhemyakina[1], Karina Zolotareva[1,2], Anna Kazachek[1,2], Dmitry Oshchepkov[1,2], Anton Bogomolov[1,2], Natalya V. Klimova[1], Vladimir A. Ivanisenko[1,2], Pavel Demenkov[1], Zakhar Mustafin[1,2], Arcady Markel[1,2], Ludmila Savinkova[1], Nikolay A. Kolchanov[1,2], Vladimir Kozlov[3], Mikhail Ponomarenko[1,*]

[1]Institute of Cytology and Genetics, Novosibirsk 630090, Russia
[2]Novosibirsk State University, Novosibirsk 630090, Russia
[3]Research Institute of Fundamental and Clinical Immunology (RIFCI SB RAS), Novosibirsk, Russia
Correspondence: pon@bionet.nsc.ru. Tel.: +7 (383) 363-49-63 ext. 1311 (M.P.)

Abstract: Mainstream transcriptome profiling of susceptibility versus resistance to age-related diseases (ARDs) is focused on differentially expressed genes (DEGs) specific to gender, age, and pathogeneses. This approach fits in well with predictive preventive personalized participatory medicine and helps understand how, why, when, and what ARD anyone can develop depending on the genetic background. Within this mainstream paradigm, we wanted to find out whether the known ARD-linked DEGs available in PubMed can reveal a molecular marker that will serve the purpose in anyone's any tissue at any time. We sequenced the periaqueductal gray (PAG) transcriptome of tame versus aggressive rats, identified rat behavior-related DEGs and compared them with their known homologous animal ARD-linked DEGs. This analysis yielded statistically significant correlations between behavior-related and ARD susceptibility-related fold changes (log2 values) in the expression of these DEG homologs. We found principal components, PC1 and PC2, corresponding to the half-sum and the half-difference of these log2 values, respectively. With the DEGs linked to ARD susceptibility and ARD resistance in humans used as controls, we verified these principal components. This yielded only one statistically significant common molecular marker for ARDs: an excess of Fcγ receptor IIb suppressing immune cell hyperactivation.

Keywords: human; age-related disease; molecular marker; *Rattus norvegicus*; RNA-Seq; qPCR; differentially expressed gene; meta-analysis; correlation; principal component; bootstrap

CONTENTS

Table S1. Novel DEGs in the periaqueductal gray matter (PAG) of the rats in comparison with their known homologs in animal-based human ARD models.

Table S2. Novel PAG-related rat DEGs in comparison with their known homologs in the binary "susceptibility versus resistance" models of human ARDs.

Table S3. Effects of underexpression or overexpression of the human genes homologous to the novel PAG-related rat DEGs on ARD development.

Section S1. Supplementary methods for DNA sequence analysis.

Figure S1. The result obtained with our web service SNP_TATA_Comparator [219] applied to only one SNP rs780467580 within the 70-bp proximal promoter of human FCGR2B.

Table S4. BLAST-based [232] phylostratigraphic age indices (PAIs) of 39 human genes homologous to the behavior-related PAG-associated rat DEGs (Table 2) and 48 human FCGR2B-associated genes (Figure 5).

Table S1. Novel DEGs in the periaqueductal gray matter (PAG) of the rats in comparison with their known homologs in animal-based human ARD models.

#	PAG, tame *vs* aggressive rats			DEGs in ARD-susceptible *vs* ARD-resistant animals							
	DEG	**log₂**	**P_ADJ**	**Species**	**ARD susceptibility**	**ARD resistance**	**Tissue**	***DEG***	**log₂**	**P_ADJ**	**Ref**
i	ii	iii	iv	v	vi	vii	viii	ix	x	xi	xii
1	*Amy1a*	1.97	0.05	mice	PolG D257A, cardiac disorder	wild-type norm	heart right ventricle	*Amy1a*	−1.00	0.05	[58]
2	*Amy1a*	1.97	0.05	mice	80-week-old, wild-type, 60% diet	80-week-old, wild-type, *ad libitum*	skeletal muscle	*Amy1*	0.47	0.05	[61]
3	*Amy1a*	1.97	0.05	mice	20-week-old, Sirt1-KI, 60% diet	20-week-old, Sirt1-KI, *ad libitum*	skeletal muscle	*Amy1*	0.46	0.05	[61]
4	*Amy1a*	1.97	0.05	mice	80-week-old, Sirt1-KI, 60% diet	80-week-old, Sirt1-KI, *ad libitum*	skeletal muscle	*Amy1*	0.61	10^{-3}	[61]
5	*Amy1a*	1.97	0.05	mice	80-week-old, Sirt1-KO, *ad libitum*	80-week-old, wild-type, *ad libitum*	skeletal muscle	*Amy1*	0.88	10^{-2}	[61]
6	*Amy1a*	1.97	0.05	mice	80-week-old, wild-type, *ad libitum*	80-week-old, Sirt1-KI, *ad libitum*	skeletal muscle	*Amy1*	−0.65	0.05	[61]
7	*Amy1a*	1.97	0.05	chicken	456-day-old, subfertility	224-day-old, fertility peak	ovary	*Amy2a*	1.14	0.05	[34]
8	*Aox1*	1.83	0.05	rat	20-passage-old	5-passage-old	MSC(BM) cells	*Aox1*	1.21	10^{-203}	[35]
9	*Aox1*	1.83	0.05	rat	20-passage-old	20-passage-old, Aspirin	MSC(BM) cells	*Aox1*	−0.15	10^{-2}	[35]
10	*Aox1*	1.83	0.05	rat	20-passage-old	5-passage-old	MSC(BM) cells	*Aox2*	−1.16	10^{-203}	[35]
11	*Aox1*	1.83	0.05	rat	20-passage-old	5-passage-old	MSC(BM) cells	*Aox3*	4.06	10^{-11}	[35]
12	*Aox1*	1.83	0.05	rat	20-passage-old	5-passage-old	MSC(BM) cells	*Aox4*	6.99	10^{-9}	[35]
13	*Aox1*	1.83	0.05	rat	ISIAH (hypertensive aged vessels)	WAG (norm)	renal medulla	*Aox1*	−1.73	10^{-2}	[49]
14	*Aox1*	1.83	0.05	rat	ISIAH (hypertensive aged vessels)	WAG (norm)	renal medulla	*Aox2*	1.32	10^{-2}	[49]
15	*Aox1*	1.83	0.05	rat	ISIAH (hypertensive aged vessels)	WAG (norm)	renal cortex	*Aox1*	−1.26	0.05	[50]
16	*Aox1*	1.83	0.05	rat	ISIAH (hypertensive aged vessels)	WAG (norm)	adrenal gland	*Aox1*	1.74	10^{-2}	[51]
17	*Aox1*	1.83	0.05	rat	ISIAH (hypertensive aged vessels)	WAG (norm)	adrenal gland	*Aox3*	0.65	0.05	[51]
18	*Aox1*	1.83	0.05	rat	ISIAH (hypertensive aged vessels)	WAG (norm)	hypothalamus	*Aox2*	1.47	10^{-2}	[48]
19	*Aox1*	1.83	0.05	mice	20-week-old, Sirt1-KO, *ad libitum*	20-week-old, wild-type, *ad libitum*	skeletal muscle	*Aox1*	−0.36	0.05	[61]
20	*Ascl3*	2.74	10^{-4}	rat	OXYS : spurred aging, 18-month-old	Wistar, 18-month-old	prefrontal cortex	*Ascl1*	0.44	10^{-2}	[45]
21	*Ascl3*	2.74	10^{-4}	chicken	456-day-old, subfertility	527-day-old, renewal fasting-diet	ovary	*Ascl4*	2.51	10^{-2}	[34]
22	*Banp*	−0.64	0.05	rat	ISIAH (hypertensive aged vessels)	WAG (norm)	renal medulla	*Banp*	−0.68	0.05	[49]
23	*Banp*	−0.64	0.05	rat	20-passage-old	5-passage-old	MSC(BM) cells	*Banp*	0.73	10^{-17}	[35]
24	*Banp*	−0.64	0.05	mice	80-week-old, wild-type, 60% diet	80-week-old, wild-type, *ad libitum*	skeletal muscle	*Banp*	−0.74	0.05	[61]
25	*Banp*	−0.64	0.05	mice	20-week-old, wild-type, 60% diet	20-week-old, wild-type, *ad libitum*	skeletal muscle	*Banp*	−1.44	0.05	[61]
26	*Banp*	−0.64	0.05	mice	20-week-old, Sirt1-KO, 60% diet	20-week-old, Sirt1-KO, *ad libitum*	skeletal muscle	*Banp*	0.74	10^{-2}	[61]

Table S1 (continued).

#	PAG, tame *vs* aggressive rats			DEGs in ARD-susceptible *vs* ARD-resistant animals							
	DEG	log2	PADJ	Species	ARD susceptibility	ARD resistance	Tissue	*DEG*	log2	PADJ	Ref
i	ii	iii	iv	v	vi	vii	viii	ix	x	xi	xii
27	*Banp*	−0.64	0.05	mice	80-week-old, Sirt1-KO, 60% diet	80-week-old, Sirt1-KO, *ad libitum*	skeletal muscle	*Banp*	−0.95	10^{-2}	[61]
28	*Banp*	−0.64	0.05	mice	20-week-old, Sirt1-KI, 60% diet	20-week-old, Sirt1-KI, *ad libitum*	skeletal muscle	*Banp*	−1.02	10^{-2}	[61]
29	*Banp*	−0.64	0.05	mice	80-week-old, Sirt1-KI, 60% diet	80-week-old, Sirt1-KI, *ad libitum*	skeletal muscle	*Banp*	−0.63	0.05	[61]
30	*Banp*	−0.64	0.05	mice	80-week-old, Sirt1-KO, *ad libitum*	80-week-old, wild-type, *ad libitum*	skeletal muscle	*Banp*	0.99	10^{-2}	[61]
31	*Banp*	−0.64	0.05	mice	20-week-old, Sirt1-KO, *ad libitum*	20-week-old, wild-type, *ad libitum*	skeletal muscle	*Banp*	−1.09	0.05	[61]
32	*Bdkrb2*	1.62	0.05	rat	OXYS : spurred aging, 20-day-old	Wistar, 20-day-old	hippocampus	*Bdkrb2*	−0.81	10^{-2}	[44]
33	*Bdkrb2*	1.62	0.05	rat	OXYS : spurred aging, 5-month-old	Wistar, 5-month-old	hippocampus	*Bdkrb2*	1.45	10^{-5}	[44]
34	*Bdkrb2*	1.62	0.05	rat	OXYS : spurred aging, 18-month-old	Wistar, 18-month-old	hippocampus	*Bdkrb2*	−1.34	10^{-5}	[44]
35	*Bdkrb2*	1.62	0.05	rat	20-passage-old	5-passage-old	MSC(BM) cells	*Bdkrb2*	−0.72	10^{-2}	[35]
36	*Bdkrb2*	1.62	0.05	mice	PolG D257A, cardiac disorder	wild-type norm	heart right ventricle	*Bdkrb2*	1.95	10^{-3}	[58]
37	*Bdkrb2*	1.62	0.05	mice	8h/8h-biorhythm (autistic-like aging)	norm	hippocampus	*Bdkrb1*	2.58	0.05	[60]
38	*Cartpt*	3.53	10^{-7}	rat	OXYS : spurred aging, 20-day-old	Wistar, 20-day-old	hippocampus	*Cartpt*	0.93	10^{-2}	[44]
39	*Cartpt*	3.53	10^{-7}	rat	ISIAH (hypertensive aged vessels)	WAG (norm)	adrenal gland	*Cartpt*	−3.09	10^{-2}	[51]
40	*Cyp2j10*	1.10	10^{-2}	rat	ISIAH (hypertensive aged vessels)	WAG (norm)	brain stem	*Cyp2j10*	−0.79	10^{-2}	[47]
41	*Cyp2j10*	1.10	10^{-2}	rat	ISIAH (hypertensive aged vessels)	WAG (norm)	hypothalamus	*Cyp2j10*	−0.94	0.05	[48]
42	*Cyp2j10*	1.10	10^{-2}	rat	ISIAH (hypertensive aged vessels)	WAG (norm)	renal cortex	*Cyp2j4*	−0.81	0.05	[50]
43	*Cyp2j10*	1.10	10^{-2}	rat	ISIAH (hypertensive aged vessels)	WAG (norm)	renal medulla	*Cyp2j5l*	0.78	10^{-2}	[49]
44	*Cyp2j10*	1.10	10^{-2}	rat	20-passage-old	5-passage-old	MSC(BM) cells	*Cyp2j10*	3.15	0.05	[35]
45	*Cyp2j10*	1.10	10^{-2}	rat	20-passage-old	5-passage-old	MSC(BM) cells	*Cyp2j3*	4.20	10^{-2}	[35]
46	*Cyp2j10*	1.10	10^{-2}	rat	20-passage-old	5-passage-old	MSC(BM) cells	*Cyp2j4*	2.75	10^{-8}	[35]
47	*Cyp2j10*	1.10	10^{-2}	mice	23-month-old, bone fragility	2-month-old	bone	*Cyp2j6*	−0.56	0.05	[55]
48	*Cyp2j10*	1.10	10^{-2}	mice	30-month-old, bone fragility	2-month-old	bone	*Cyp2j6*	−0.75	10^{-5}	[55]
49	*Cyp2j10*	1.10	10^{-2}	mice	BPH/2l hypertensive	BPN/3J, norm	kidney	*Cyp2j6*	1.40	10^{-4}	[62]
50	*Cyp2j10*	1.10	10^{-2}	mice	BPH/2l hypertensive	BPN/3J, norm	kidney	*Cyp2j9*	1.22	0.05	[62]

Table S1 (continued).

#	PAG, tame *vs* aggressive rats			DEGs in ARD-susceptible *vs* ARD-resistant animals							
	DEG	**log2**	**PADJ**	**Species**	**ARD susceptibility**	**ARD resistance**	**Tissue**	***DEG***	**log2**	**PADJ**	**Ref**
i	ii	iii	iv	v	vi	vii	viii	ix	x	xi	xii
51	*Defb17*	6.96	10^{-2}	rat	ISIAH (hypertensive aged vessels)	WAG (norm)	renal cortex	*Defb1*	0.59	0.05	[50]
52	*Defb17*	6.96	10^{-2}	mice	8h/8h-biorhythm (autistic-like aging)	norm	hippocampus	*Defb2*	4.00	0.05	[60]
53	*Defb17*	6.96	10^{-2}	chicken	1.2% Ca diet: the worst health	0.8% Ca diet: the best health	kidney	*Avbd1*	1.33	10^{-2}	[30]
54	*Defb17*	6.96	10^{-2}	chicken	1.2% Ca diet: the worst health	0.8% Ca diet: the best health	kidney	*Avbd7*	1.36	10^{-2}	[30]
55	*Defb17*	6.96	10^{-2}	chicken	1.2% Ca diet: the worst health	0.8% Ca diet: the best health	kidney	*Defb4a*	1.41	10^{-2}	[30]
56	*Defb17*	6.96	10^{-2}	chicken	1.2% Ca diet: the worst health	1% Ca diet limits health	kidney	*Avbd1*	1.48	10^{-2}	[30]
57	*Defb17*	6.96	10^{-2}	chicken	1.2% Ca diet: the worst health	1% Ca diet limits health	kidney	*Avbd7*	1.40	10^{-2}	[30]
58	*Defb17*	6.96	10^{-2}	chicken	1.2% Ca diet: the worst health	1% Ca diet limits health	kidney	*Defb4a*	3.27	10^{-2}	[30]
59	*Emx2*	1.45	10^{-2}	rat	20-passage-old	5-passage-old	MSC(BM) cells	*Emx2*	0.68	10^{-9}	[35]
60	*Fat2*	−4.96	0.05	rat	ISIAH (hypertensive aged vessels)	WAG (norm)	brain stem	*Fat2*	0.94	10^{-2}	[47]
61	*Fat2*	−4.96	0.05	rat	ISIAH (hypertensive aged vessels)	WAG (norm)	renal cortex	*Fat3*	−2.86	0.05	[50]
62	*Fat2*	−4.96	0.05	rat	20-passage-old	5-passage-old	MSC(BM) cells	*Fat1*	−1.51	10^{-305}	[35]
63	*Fat2*	−4.96	0.05	rat	20-passage-old	5-passage-old	MSC(BM) cells	*Fat4*	1.90	10^{-170}	[35]
64	*Fat2*	−4.96	0.05	rat	5-passage-old	5-passage-old, Aspirin	MSC(BM) cells	*Fat1*	0.08	0.05	[35]
65	*Fat2*	−4.96	0.05	mice	11-month-old, bone fragility	2-month-old	bone	*Fat3*	−1.52	10^{-3}	[55]
66	*Fat2*	−4.96	0.05	mice	23-month-old, bone fragility	2-month-old	bone	*Fat3*	−0.75	0.05	[55]
67	*Fat2*	−4.96	0.05	mice	23-month-old, bone fragility	2-month-old	bone	*Fat4*	−0.89	10^{-2}	[55]
68	*Fat2*	−4.96	0.05	mice	30-month-old, bone fragility	2-month-old	bone	*Fat3*	−0.89	0.05	[55]
69	*Fat2*	−4.96	0.05	mice	30-month-old, bone fragility	2-month-old	bone	*Fat4*	−1.35	10^{-20}	[55]
70	*Fat2*	−4.96	0.05	mice	80-week-old, wild-type, 60% diet	80-week-old, wild-type, *ad libitum*	skeletal muscle	*Fat4*	−0.86	0.05	[61]
71	*Fat2*	−4.96	0.05	mice	80-week-old, Sirt1-KI, 60% diet	80-week-old, Sirt1-KI, *ad libitum*	skeletal muscle	*Fat3*	−0.88	0.05	[61]
72	*Fat2*	−4.96	0.05	mice	20-week-old, Sirt1-KI, 60% diet	20-week-old, Sirt1-KI, *ad libitum*	skeletal muscle	*Fat1*	−0.79	0.05	[61]
73	*Fat2*	−4.96	0.05	chicken	469-day-old, spurt aging by hunger	456-day-old, subfertility	ovary	*Fat3*	1.21	0.05	[34]

able S1 (continued).

#	PAG, tame *vs* aggressive rats			DEGs in ARD-susceptible *vs* ARD-resistant animals							
	DEG	log2	PADJ	Species	ARD susceptibility	ARD resistance	Tissue	*DEG*	log2	PADJ	Ref
i	ii	iii	iv	v	vi	vii	viii	ix	x	xi	xii
74	*Fat2*	−4.96	0.05	fruit fly	10-day-old, Alzheimer disease-like	0-day-old, just post-eclosion	head	*tf (Fat4)*	−2.65	10^{-3}	[64]
75	*Fcgr2b*	2.01	0.05	rat	20-passage-old	20-passage-old, Aspirin	MSC(BM) cells	*Fcgr2b*	1.00	10^{-6}	[35]
76	*Fcgr2b*	2.01	0.05	rat	20-passage-old	5-passage-old	MSC(BM) cells	*Fcgr2b*	6.32	10^{-3}	[35]
77	*Fcgr2b*	2.01	0.05	rat	20-passage-old	5-passage-old	MSC(BM) cells	*Fcgr2a*	3.99	10^{-80}	[35]
78	*Fcgr2b*	2.01	0.05	rat	20-passage-old	5-passage-old	MSC(BM) cells	*Fcgr3a*	2.86	10^{-44}	[35]
79	*Fcgr2b*	2.01	0.05	rat	ISIAH (hypertensive aged vessels)	WAG (norm)	adrenal gland	*Fcgr1a*	0.76	0.05	[51]
80	*Fcgr2b*	2.01	0.05	rat	ISIAH (hypertensive aged vessels)	WAG (norm)	adrenal gland	*Fcgr3a*	0.67	0.05	[51]
81	*Fcgr2b*	2.01	0.05	mice	30-month-old, bone fragility	2-month-old	bone	*Fcgr2b*	1.11	10^{-3}	[55]
82	*Fcgr2b*	2.01	0.05	mice	30-month-old, bone fragility	2-month-old	bone	*Fcgr1*	1.12	10^{-2}	[55]
83	*Fcgr2b*	2.01	0.05	mice	30-month-old, bone fragility	2-month-old	bone	*Fcgr3*	0.92	10^{-2}	[55]
84	*Fcgr2b*	2.01	0.05	mice	27-month-old, male	2-month-old, male	kidney	*Fcgr3*	1.39	0.05	[56]
85	*Fcgr2b*	2.01	0.05	mice	PolG D257A, cardiac disorder	wild-type norm	heart right ventricle	*Fcgr4*	1.91	10^{-2}	[58]
86	*Fosb*	−1.85	0.05	rat	20-passage-old	5-passage-old	MSC(BM) cells	*Fosb*	0.90	10^{-9}	[35]
87	*Fosb*	−1.85	0.05	rat	20-passage-old	5-passage-old	MSC(BM) cells	*Fos*	1.36	10^{-2}	[35]
88	*Fosb*	−1.85	0.05	rat	20-passage-old	5-passage-old	MSC(BM) cells	*Fosl1*	−2.64	10^{-163}	[35]
89	*Fosb*	−1.85	0.05	rat	20-passage-old	5-passage-old	MSC(BM) cells	*Fosl2*	0.43	0.05	[35]
90	*Fosb*	−1.85	0.05	mice	27-month-old, male	2-month-old, male	kidney	*Fos*	−1.04	0.05	[56]
91	*Fosb*	−1.85	0.05	mice	20-week-old, Sirt1-KO, 60% diet	20-week-old, Sirt1-KO, *ad libitum*	skeletal muscle	*Fos*	0.75	0.05	[61]
92	*Fosb*	−1.85	0.05	mice	20-week-old, Sirt1-KO, 60% diet	20-week-old, Sirt1-KO, *ad libitum*	skeletal muscle	*Fosl2*	0.83	0.05	[61]
93	*Fosb*	−1.85	0.05	mice	80-week-old, Sirt1-KI, 60% diet	80-week-old, Sirt1-KI, *ad libitum*	skeletal muscle	*Fos*	1.19	10^{-2}	[61]
94	*Fosb*	−1.85	0.05	mice	80-week-old, Sirt1-KO, 60% diet	80-week-old, wild-type, *ad libitum*	skeletal muscle	*Fos*	1.40	0.05	[61]
95	*Fosb*	−1.85	0.05	chicken	1% Ca diet limits health	0.8% Ca diet: the best health	kidney	*Fos*	0.81	10^{-2}	[30]
96	*Fosb*	−1.85	0.05	chicken	1.2% Ca diet: the worst health	1% Ca diet limits health	kidney	*Fos*	−0.75	10^{-2}	[30]
97	*Gpd1*	−0.99	0.05	rat	ISIAH (hypertensive aged vessels)	WAG (norm)	renal medulla	*Gpd1*	0.44	0.05	[49]
98	*Gpd1*	−0.99	0.05	rat	20-passage-old	5-passage-old	MSC(BM) cells	*Gpd1l*	0.74	10^{-6}	[35]
99	*Gpd1*	−0.99	0.05	rat	20-passage-old	5-passage-old	MSC(BM) cells	*Gpd2*	−0.31	10^{-4}	[35]
100	*Gpd1*	−0.99	0.05	mice	30-month-old, bone fragility	2-month-old	bone	*Gpd1*	−1.34	0.05	[55]

Table S1 (continued).

#	PAG, tame *vs* aggressive rats			DEGs in ARD-susceptible *vs* ARD-resistant animals							
	DEG	**log₂**	**P_{ADJ}**	**Species**	**ARD susceptibility**	**ARD resistance**	**Tissue**	***DEG***	**log₂**	P_{ADJ}	Ref
i	ii	iii	iv	v	vi	vii	viii	ix	x	xi	xii
101	*Gpd1*	−0.99	0.05	mice	24-month-old, male	3-month-old, male	kidney	*Gpd1*	1.21	0.05	[57]
102	*Gpd1*	−0.99	0.05	mice	80-week-old, Sirt1-KI, 60% diet	80-week-old, Sirt1-KI, *ad libitum*	skeletal muscle	*Gpd1*	−0.38	10^{-2}	[61]
103	*Gpd1*	−0.99	0.05	mice	80-week-old, Sirt1-KI, 60% diet	80-week-old, Sirt1-KI, *ad libitum*	skeletal muscle	*Gpd2*	−0.60	10^{-5}	[61]
104	*Gpd1*	−0.99	0.05	mice	80-week-old, wild-type, *ad libitum*	80-week-old, Sirt1-KI, *ad libitum*	skeletal muscle	*Gpd2*	−0.52	10^{-2}	[61]
105	*Hbb-b1*	−5.90	10^{-5}	rat	ISIAH (hypertensive aged vessels)	WAG (norm)	brain stem	*Hbb-b1*	1.42	10^{-2}	[47]
106	*Hbb-b1*	−5.90	10^{-5}	rat	ISIAH (hypertensive aged vessels)	WAG (norm)	brain stem	*Hbb-b*	1.88	10^{-2}	[47]
107	*Hbb-b1*	−5.90	10^{-5}	rat	ISIAH (hypertensive aged vessels)	WAG (norm)	hypothalamus	*Hbb-b1*	2.02	10^{-2}	[48]
108	*Hbb-b1*	−5.90	10^{-5}	rat	ISIAH (hypertensive aged vessels)	WAG (norm)	hypothalamus	*Hbb*	3.23	10^{-2}	[48]
109	*Hbb-b1*	−5.90	10^{-5}	rat	ISIAH (hypertensive aged vessels)	WAG (norm)	renal medulla	*Hbb-b1*	1.18	10^{-2}	[49]
110	*Hbb-b1*	−5.90	10^{-5}	rat	ISIAH (hypertensive aged vessels)	WAG (norm)	renal medulla	*Hbb*	−0.68	10^{-2}	[49]
111	*Hbb-b1*	−5.90	10^{-5}	rat	ISIAH (hypertensive aged vessels)	WAG (norm)	renal medulla	*Hbb-bs*	2.38	10^{-2}	[49]
112	*Hbb-b1*	−5.90	10^{-5}	rat	ISIAH (hypertensive aged vessels)	WAG (norm)	adrenal gland	*Hbb-b1*	1.32	10^{-2}	[51]
113	*Hbb-b1*	−5.90	10^{-5}	rat	ISIAH (hypertensive aged vessels)	WAG (norm)	adrenal gland	*Hbbs*	2.02	10^{-2}	[51]
114	*Hbb-b1*	−5.90	10^{-5}	mice	11-month-old, bone fragility	2-month-old	bone	*Hbb-b1*	1.17	0.05	
115	*Hbb-b1*	−5.90	10^{-5}	mice	PolG D257A, cardiac disorder	wild-type norm	heart right ventricle	*Hbb-bs*	1.30	10^{-4}	[58]
116	*Hbb-b1*	−5.90	10^{-5}	mice	80-week-old, wild-type, 60% diet	80-week-old, wild-type, *ad libitum*	skeletal muscle	*Hbb-bs*	−0.92	10^{-2}	[61]
117	*Hbb-b1*	−5.90	10^{-5}	mice	80-week-old, wild-type, 60% diet	80-week-old, wild-type, *ad libitum*	skeletal muscle	*Hb-bt*	−1.51	0.05	[61]
118	*Hbb-b1*	−5.90	10^{-5}	mice	20-week-old, Sirt1-KO, 60% diet	20-week-old, Sirt1-KO, *ad libitum*	skeletal muscle	*Hbb-bs*	0.87	10^{-2}	[61]
119	*Hbb-b1*	−5.90	10^{-5}	mice	20-week-old, Sirt1-KO, 60% diet	20-week-old, Sirt1-KO, *ad libitum*	skeletal muscle	*Hb-bt*	0.90	0.05	[61]
120	*Hbb-b1*	−5.90	10^{-5}	mice	80-week-old, Sirt1-KO, 60% diet	80-week-old, Sirt1-KO, *ad libitum*	skeletal muscle	*Hbb-bs*	2.20	10^{-3}	[61]
121	*Hbb-b1*	−5.90	10^{-5}	mice	80-week-old, Sirt1-KO, 60% diet	80-week-old, Sirt1-KO, *ad libitum*	skeletal muscle	*Hb-bt*	2.19	10^{-3}	[61]

Table S1 (continued).

#	PAG, tame *vs* aggressive rats			DEGs in ARD-susceptible *vs* ARD-resistant animals							
	DEG	**log$_2$**	**P$_{ADJ}$**	**Species**	**ARD susceptibility**	**ARD resistance**	**Tissue**	***DEG***	**log$_2$**	P$_{ADJ}$	Ref
i	ii	iii	iv	v	vi	vii	viii	ix	x	xi	xii
122	*Hbb-b1*	−5.90	10^{-5}	mice	20-week-old, Sirt1-KI, 60% diet	20-week-old, Sirt1-KI, *ad libitum*	skeletal muscle	*Hbb-bs*	−0.57	0.05	[61]
123	*Hbb-b1*	−5.90	10^{-5}	mice	20-week-old, Sirt1-KI, 60% diet	20-week-old, Sirt1-KI, *ad libitum*	skeletal muscle	*Hb-bt*	−1.04	10^{-2}	[61]
124	*Hspa1b*	−2.14	10^{-7}	rat	20-passage-old	5-passage-old	MSC(BM) cells	*Hspa1b*	1.36	10^{-55}	[35]
125	*Hspa1b*	−2.14	10^{-7}	rat	20-passage-old	5-passage-old	MSC(BM) cells	*Hspa12a*	−1.44	10^{-28}	[35]
126	*Hspa1b*	−2.14	10^{-7}	rat	20-passage-old	5-passage-old	MSC(BM) cells	*Hspa12b*	1.99	10^{-111}	[35]
127	*Hspa1b*	−2.14	10^{-7}	rat	20-passage-old	5-passage-old	MSC(BM) cells	*Hspa1a*	1.12	10^{-3}	[35]
128	*Hspa1b*	−2.14	10^{-7}	rat	20-passage-old	5-passage-old	MSC(BM) cells	*Hspa1l*	−0.66	0.05	[35]
129	*Hspa1b*	−2.14	10^{-7}	rat	20-passage-old	5-passage-old	MSC(BM) cells	*Hspa2*	0.32	10^{-2}	[35]
130	*Hspa1b*	−2.14	10^{-7}	rat	20-passage-old	5-passage-old	MSC(BM) cells	*Hspa4*	−0.98	10^{-109}	[35]
131	*Hspa1b*	−2.14	10^{-7}	rat	20-passage-old	5-passage-old	MSC(BM) cells	*Hspa4l*	−0.63	0.05	[35]
132	*Hspa1b*	−2.14	10^{-7}	rat	20-passage-old	5-passage-old	MSC(BM) cells	*Hspa5*	−0.91	10^{-160}	[35]
133	*Hspa1b*	−2.14	10^{-7}	rat	20-passage-old	5-passage-old	MSC(BM) cells	*Hspa8*	0.18	10^{-5}	[35]
134	*Hspa1b*	−2.14	10^{-7}	rat	20-passage-old	5-passage-old	MSC(BM) cells	*Hspa9*	−0.53	10^{-32}	[35]
135	*Hspa1b*	−2.14	10^{-7}	rat	20-passage-old	20-passage-old, Aspirin	MSC(BM) cells	*Hspa1b*	0.25	0.05	[35]
136	*Hspa1b*	−2.14	10^{-7}	rat	5-passage-old	5-passage-old, Aspirin	MSC(BM) cells	*Hspa4*	0.11	0.05	[35]
137	*Hspa1b*	−2.14	10^{-7}	rat	5-passage-old	5-passage-old, Aspirin	MSC(BM) cells	*Hspa5*	0.24	10^{-25}	[35]
138	*Hspa1b*	−2.14	10^{-7}	rat	5-passage-old	5-passage-old, Aspirin	MSC(BM) cells	*Hspa8*	0.12	10^{-4}	[35]
139	*Hspa1b*	−2.14	10^{-7}	rat	ISIAH (hypertensive aged vessels)	WAG (norm)	adrenal gland	*Hspa1a*	0.70	0.05	[51]
140	*Hspa1b*	−2.14	10^{-7}	rat	ISIAH (hypertensive aged vessels)	WAG (norm)	adrenal gland	*Hspa4l*	−0.55	0.05	[51]
141	*Hspa1b*	−2.14	10^{-7}	rat	ISIAH (hypertensive aged vessels)	WAG (norm)	adrenal gland	*Hspa6l*	0.90	0.05	[51]
142	*Hspa1b*	−2.14	10^{-7}	mice	24-month-old, male	3-month-old, male	kidney	*Hspa1b*	−1.46	10^{-2}	[57]
143	*Hspa1b*	−2.14	10^{-7}	mice	24-month-old, male	3-month-old, male	kidney	*Hspa1a*	−1.47	0.05	[57]
144	*Hspa1b*	−2.14	10^{-7}	mice	24-month-old, male	3-month-old, male	kidney	*Hspa4*	−1.22	0.05	[57]
145	*Hspa1b*	−2.14	10^{-7}	mice	24-month-old, male	3-month-old, male	kidney	*Hspa5*	−1.28	10^{-3}	[57]
146	*Hspa1b*	−2.14	10^{-7}	mice	24-month-old, male	3-month-old, male	kidney	*Hspa8*	−1.48	0.05	[57]
147	*Hspa1b*	−2.14	10^{-7}	mice	20-week-old, Sirt1-KO, 60% diet	20-week-old, Sirt1-KO, *ad libitum*	skeletal muscle	*Hspa1b*	0.91	10^{-2}	[61]
148	*Hspa1b*	−2.14	10^{-7}	mice	80-week-old, Sirt1-KI, 60% diet	80-week-old, Sirt1-KI, *ad libitum*	skeletal muscle	*Hspa1b*	−1.03	10^{-2}	[61]
149	*Hspa1b*	−2.14	10^{-7}	mice	20-week-old, Sirt1-KO, 60% diet	20-week-old, Sirt1-KO, *ad libitum*	skeletal muscle	*Hspa1a*	0.77	10^{-2}	[61]
150	*Hspa1b*	−2.14	10^{-7}	mice	80-week-old, Sirt1-KO, 60% diet	80-week-old, wild-type, *ad libitum*	skeletal muscle	*Hspa12b*	−0.57	0.05	[61]

Table S1 (continued).

#	PAG, tame *vs* aggressive rats			DEGs in ARD-susceptible *vs* ARD-resistant animals							
	DEG	log2	PADJ	Species	ARD susceptibility	ARD resistance	Tissue	*DEG*	log2	PADJ	Ref
i	ii	iii	iv	v	vi	vii	viii	ix	x	xi	xii
151	*Hspa1b*	−2.14	10^{-7}	mice	80-week-old, Sirt1-KO, 60% diet	80-week-old, wild-type, *ad libitum*	skeletal muscle	*Hspa8*	0.50	0.05	[61]
152	*Hspa1b*	−2.14	10^{-7}	mice	20-week-old, Sirt1-KO, 60% diet	20-week-old, Sirt1-KO, *ad libitum*	skeletal muscle	*Hspa1l*	−0.42	0.05	[61]
153	*Hspa1b*	−2.14	10^{-7}	mice	20-week-old, Sirt1-KO, 60% diet	20-week-old, Sirt1-KO, *ad libitum*	skeletal muscle	*Hspa8*	−0.68	10^{-3}	[61]
154	*Hspa1b*	−2.14	10^{-7}	mice	20-week-old, Sirt1-KI, 60% diet	20-week-old, Sirt1-KI, *ad libitum*	skeletal muscle	*Hspa13*	−0.40	0.05	[61]
155	*Hspa1b*	−2.14	10^{-7}	mice	20-week-old, Sirt1-KI, 60% diet	20-week-old, Sirt1-KI, *ad libitum*	skeletal muscle	*Hspa4l*	−0.47	0.05	[61]
156	*Hspa1b*	−2.14	10^{-7}	mice	20-week-old, Sirt1-KI, 60% diet	20-week-old, Sirt1-KI, *ad libitum*	skeletal muscle	*Hspa8*	−0.58	10^{-2}	[61]
157	*Hspa1b*	−2.14	10^{-7}	mice	80-week-old, Sirt1-KI, 60% diet	80-week-old, Sirt1-KI, *ad libitum*	skeletal muscle	*Hspa1a*	−0.47	0.05	[61]
158	*Hspa1b*	−2.14	10^{-7}	mice	80-week-old, Sirt1-KI, 60% diet	80-week-old, Sirt1-KI, *ad libitum*	skeletal muscle	*Hspa4l*	−0.65	10^{-2}	[61]
159	*Hspa1b*	−2.14	10^{-7}	mice	80-week-old, Sirt1-KI, 60% diet	80-week-old, Sirt1-KI, *ad libitum*	skeletal muscle	*Hspa5*	0.34	10^{-3}	[61]
160	*Hspa1b*	−2.14	10^{-7}	mice	23-month-old, bone fragility	2-month-old	bone	*Hspa12a*	−0.77	10^{-3}	[55]
161	*Hspa1b*	−2.14	10^{-7}	mice	30-month-old, bone fragility	2-month-old	bone	*Hspa12a*	−1.15	10^{-8}	[55]
162	*Hspa1b*	−2.14	10^{-7}	chicken	456-day-old, subfertility	224-day-old, fertility peak	ovary	*Hspa2*	3.00	0.05	[34]
163	*Hspa1b*	−2.14	10^{-7}	chicken	469-day-old, spurt aging by hunger	456-day-old, subfertility	ovary	*Hspa2*	−3.25	10^{-2}	[34]
164	*Hspa1b*	−2.14	10^{-7}	chicken	469-day-old, spurt aging by hunger	456-day-old, subfertility	ovary	*Hspa5*	−1.06	10^{-2}	[34]
165	*Itm2a*	0.76	10^{-2}	rat	20-passage-old	20-passage-old, Aspirin	MSC(BM) cells	*Itm2a*	−0.51	10^{-31}	[35]
166	*Itm2a*	0.76	10^{-2}	rat	5-passage-old	5-passage-old, Aspirin	MSC(BM) cells	*Itm2c*	−0.12	10^{-3}	[35]
167	*Itm2a*	0.76	10^{-2}	rat	20-passage-old	5-passage-old	MSC(BM) cells	*Itm2a*	6.99	10^{-305}	[35]
168	*Itm2a*	0.76	10^{-2}	rat	20-passage-old	5-passage-old	MSC(BM) cells	*Itm2b*	0.52	10^{-58}	[35]
169	*Itm2a*	0.76	10^{-2}	rat	20-passage-old	5-passage-old	MSC(BM) cells	*Itm2c*	0.80	10^{-107}	[35]
170	*Itm2a*	0.76	10^{-2}	mice	11-month-old, bone fragility	2-month-old	bone	*Itm2a*	−1.36	10^{-2}	[55]
171	*Itm2a*	0.76	10^{-2}	mice	11-month-old, bone fragility	2-month-old	bone	*Itm2c*	−0.69	0.05	[55]
172	*Itm2a*	0.76	10^{-2}	mice	23-month-old, bone fragility	2-month-old	bone	*Itm2a*	−1.87	10^{-9}	[55]
173	*Itm2a*	0.76	10^{-2}	mice	30-month-old, bone fragility	2-month-old	bone	*Itm2a*	−2.69	10^{-26}	[55]
174	*Itm2a*	0.76	10^{-2}	mice	80-week-old, wild-type, 60% diet	80-week-old, wild-type, *ad libitum*	skeletal muscle	*Itm2a*	−1.00	0.05	[61]

Table S1 (continued).

#	PAG, tame *vs* aggressive rats			DEGs in ARD-susceptible *vs* ARD-resistant animals							
	DEG	log2	P_{ADJ}	Species	ARD susceptibility	ARD resistance	Tissue	*DEG*	log2	P_{ADJ}	Ref
i	ii	iii	iv	v	vi	vii	viii	ix	x	xi	xii
175	*Itm2a*	0.76	10^{-2}	mice	20-week-old, wild-type, 60% diet	20-week-old, wild-type, *ad libitum*	skeletal muscle	*Itm2a*	−0.47	10^{-2}	[61]
176	*Itm2a*	0.76	10^{-2}	mice	20-week-old, wild-type, 60% diet	20-week-old, Sirt1-KI, *ad libitum*	skeletal muscle	*Itm2c*	−0.29	0.05	[61]
177	*Itm2a*	0.76	10^{-2}	mice	20-week-old, Sirt1-KI, 60% diet	20-week-old, Sirt1-KI, *ad libitum*	skeletal muscle	*Itm2a*	−0.71	10^{-2}	[61]
178	*Itm2a*	0.76	10^{-2}	mice	80-week-old, Sirt1-KI, 60% diet	80-week-old, Sirt1-KI, *ad libitum*	skeletal muscle	*Itm2a*	−1.31	10^{-3}	[61]
179	*Itm2a*	0.76	10^{-2}	mice	80-week-old, Sirt1-KO, *ad libitum*	80-week-old, wild-type, *ad libitum*	skeletal muscle	*Itm2a*	−1.24	0.05	[61]
180	*Itm2a*	0.76	10^{-2}	mice	20-week-old, wild-type, 60% diet	20-week-old, wild-type, *ad libitum*	skeletal muscle	*Itm2c*	0.34	0.05	[61]
181	*Krt2*	−1.85	10^{-6}	rat	20-passage-old	5-passage-old	MSC(BM) cells	*Krt10*	0.79	10^{-2}	[35]
182	*Krt2*	−1.85	10^{-6}	rat	20-passage-old	5-passage-old	MSC(BM) cells	*Krt7*	−3.24	10^{-40}	[35]
183	*Krt2*	−1.85	10^{-6}	rat	20-passage-old	5-passage-old	MSC(BM) cells	*Krt71*	−4.89	10^{-2}	[35]
184	*Krt2*	−1.85	10^{-6}	rat	20-passage-old	5-passage-old	MSC(BM) cells	*Krt76*	−4.57	0.05	[35]
185	*Krt2*	−1.85	10^{-6}	rat	20-passage-old	5-passage-old	MSC(BM) cells	*Krt79*	−4.58	10^{-16}	[35]
186	*Krt2*	−1.85	10^{-6}	rat	20-passage-old	5-passage-old	MSC(BM) cells	*Krt8*	1.47	10^{-4}	[35]
187	*Krt2*	−1.85	10^{-6}	rat	ISIAH (hypertensive aged vessels)	WAG (norm)	renal medulla	*Krt15*	2.90	0.05	[49]
188	*Krt2*	−1.85	10^{-6}	rat	ISIAH (hypertensive aged vessels)	WAG (norm)	renal medulla	*Krt19*	4.18	10^{-2}	[49]
189	*Krt2*	−1.85	10^{-6}	rat	ISIAH (hypertensive aged vessels)	WAG (norm)	renal medulla	*Krt23*	−1.35	0.05	[49]
190	*Krt2*	−1.85	10^{-6}	rat	ISIAH (hypertensive aged vessels)	WAG (norm)	renal medulla	*Krt28*	0.49	0.05	[49]
191	*Krt2*	−1.85	10^{-6}	rat	ISIAH (hypertensive aged vessels)	WAG (norm)	renal cortex	*Krt19*	3.37	0.05	[50]
192	*Krt2*	−1.85	10^{-6}	rat	ISIAH (hypertensive aged vessels)	WAG (norm)	renal cortex	*Krt8*	0.47	0.05	[50]
193	*Krt2*	−1.85	10^{-6}	rat	ISIAH (hypertensive aged vessels)	WAG (norm)	adrenal gland	*Krt10*	−1.82	10^{-2}	[51]
194	*Krt2*	−1.85	10^{-6}	rat	ISIAH (hypertensive aged vessels)	WAG (norm)	adrenal gland	*Krt18*	2.82	10^{-2}	[51]
195	*Krt2*	−1.85	10^{-6}	rat	ISIAH (hypertensive aged vessels)	WAG (norm)	adrenal gland	*Krt8*	−0.63	0.05	[51]
196	*Krt2*	−1.85	10^{-6}	mice	30-month-old, bone fragility	2-month-old	bone	*Krt10*	−0.89	10^{-2}	[55]
197	*Krt2*	−1.85	10^{-6}	mice	30-month-old, bone fragility	2-month-old	bone	*Krt19*	−1.89	10^{-2}	[55]
198	*Krt2*	−1.85	10^{-6}	mice	30-month-old, bone fragility	2-month-old	bone	*Krt222*	−0.72	0.05	[55]
199	*Krt2*	−1.85	10^{-6}	mice	30-month-old, bone fragility	2-month-old	bone	*Krt7*	0.74	0.05	[55]
200	*Krt2*	−1.85	10^{-6}	mice	30-month-old, bone fragility	2-month-old	bone	*Krt80*	1.27	10^{-2}	[55]

Table S1 (continued).

#	PAG, tame *vs* aggressive rats			DEGs in ARD-susceptible *vs* ARD-resistant animals							
	DEG	log2	P_{ADJ}	Species	ARD susceptibility	ARD resistance	Tissue	*DEG*	log2	P_{ADJ}	Ref
i	ii	iii	iv	v	vi	vii	viii	ix	x	xi	xii
201	*Krt2*	−1.85	10^{-6}	mice	23-month-old, bone fragility	2-month-old	bone	*Krt222*	−0.67	0.05	[55]
202	*Krt2*	−1.85	10^{-6}	mice	80-week-old, Sirt1-KI, 60% diet	80-week-old, Sirt1-KI, *ad libitum*	skeletal muscle	*Krt47eg*	−0.85	0.05	[61]
203	*Krt2*	−1.85	10^{-6}	mice	80-week-old, Sirt1-KO, *ad libitum*	80-week-old, wild-type, *ad libitum*	skeletal muscle	*Krt222*	−1.81	10^{-2}	[61]
204	*Krt2*	−1.85	10^{-6}	mice	BPH/2l hypertensive	BPN/3J, norm	kidney	*Krt18*	1.31	10^{-2}	[62]
205	*Krt2*	−1.85	10^{-6}	rabbit	under Goldblatt 2-kidney 1-clip	norm	prefrontal cortex	*Krt7*	1.21	10^{-4}	[63]
206	*Krt2*	−1.85	10^{-6}	rabbit	under Goldblatt 2-kidney 1-clip	norm	prefrontal cortex	*Krt84*	−1.15	10^{-4}	[63]
207	*Krt2*	−1.85	10^{-6}	chicken	469-day-old, spurt aging by hunger	456-day-old, subfertility	ovary	*Krt23*	−2.47	10^{-2}	[34]
208	*Krt2*	−1.85	10^{-6}	chicken	469-day-old, spurt aging by hunger	527-day-old, renewal fasting-diet	ovary	*Krt18*	−1.40	10^{-2}	[34]
209	*Krt2*	−1.85	10^{-6}	chicken	469-day-old, spurt aging by hunger	527-day-old, renewal fasting-diet	ovary	*Krt22*	−3.38	10^{-4}	[34]
210	*Morn1*	0.75	0.05	rat	ISIAH (hypertensive aged vessels)	WAG (norm)	brain stem	*Morn1*	2.05	10^{-2}	[47]
211	*Morn1*	0.75	0.05	rat	ISIAH (hypertensive aged vessels)	WAG (norm)	hypothalamus	*Morn1*	1.44	10^{-2}	[48]
212	*Morn1*	0.75	0.05	rat	ISIAH (hypertensive aged vessels)	WAG (norm)	adrenal gland	*Morn1*	1.01	10^{-2}	[51]
213	*Morn1*	0.75	0.05	rat	20-passage-old	5-passage-old	MSC(BM) cells	*Morn2*	0.49	0.05	[35]
214	*Morn1*	0.75	0.05	rat	20-passage-old	5-passage-old	MSC(BM) cells	*Morn4*	0.63	10^{-7}	[35]
215	*Morn1*	0.75	0.05	mice	30-month-old, bone fragility	2-month-old	bone	*Morn4*	−1.20	10^{-4}	[55]
216	*Morn1*	0.75	0.05	mice	20-week-old, wild-type, 60% diet	20-week-old, wild-type, *ad libitum*	skeletal muscle	*Morn4*	1.93	10^{-3}	[61]
217	*Morn1*	0.75	0.05	mice	20-week-old, Sirt1-KI, 60% diet	20-week-old, Sirt1-KI, *ad libitum*	skeletal muscle	*Morn4*	0.99	0.05	[61]
218	*Morn1*	0.75	0.05	mice	80-week-old, Sirt1-KI, 60% diet	80-week-old, Sirt1-KI, *ad libitum*	skeletal muscle	*Morn4*	1.32	0.05	[61]
219	*Morn1*	0.75	0.05	mice	80-week-old, wild-type, 60% diet	80-week-old, wild-type, *ad libitum*	skeletal muscle	*Morn4*	−0.77	0.05	[61]
220	*Morn1*	0.75	0.05	mice	20-week-old, wild-type, 60% diet	20-week-old, wild-type, *ad libitum*	skeletal muscle	*Morn4*	1.16	0.05	[61]
221	*Morn1*	0.75	0.05	mice	8h/8h-biorhythm (autistic-like aging)	norm	hippocampus	*Morn3*	1.18	0.05	[60]
222	*Morn1*	0.75	0.05	mice	8h/8h-biorhythm (autistic-like aging)	norm	hippocampus	*Morn5*	1.16	0.05	[60]
223	*Myom2*	−1.63	0.05	rat	ISIAH (hypertensive aged vessels)	WAG (norm)	adrenal gland	*Myom2*	0.81	10^{-2}	[51]

Table S1 (continued).

#	PAG, tame *vs* aggressive rats			DEGs in ARD-susceptible *vs* ARD-resistant animals							
	DEG	**log2**	**PADJ**	**Species**	**ARD susceptibility**	**ARD resistance**	**Tissue**	***DEG***	**log2**	PADJ	Ref
i	ii	iii	iv	v	vi	vii	viii	ix	x	xi	xii
224	*Myom2*	−1.63	0.05	rat	ISIAH (hypertensive aged vessels)	WAG (norm)	brain stem	*Myom3*	−1.62	10^{-2}	[47]
225	*Myom2*	−1.63	0.05	rat	20-passage-old	5-passage-old	MSC(BM) cells	*Myom3*	−2.51	10^{-110}	[35]
226	*Myom2*	−1.63	0.05	mice	27-month-old	2-month-old	kidney	*Myom2*	−2.82	10^{-10}	[56]
227	*Myom2*	−1.63	0.05	mice	27-month-old, female	2-month-old, female	kidney	*Myom2*	−1.90	10^{-2}	[56]
228	*Myom2*	−1.63	0.05	mice	27-month-old, male	2-month-old, male	kidney	*Myom2*	−3.34	10^{-12}	[56]
229	*Myom2*	−1.63	0.05	mice	27-month-old, male	2-month-old, male	kidney	*Myom2*	−3.71	0.05	[56]
230	*Myom2*	−1.63	0.05	mice	PolG D257A, cardiac disorder	wild-type norm	heart right ventricle	*Myom2*	0.69	10^{-2}	[58]
231	*Myom2*	−1.63	0.05	mice	80-week-old, wild-type, *ad libitum*	80-week-old, Sirt1-KI, *ad libitum*(	skeletal muscle	*Myom2*	−0.36	0.05	[61]
232	*Myom2*	−1.63	0.05	mice	20-week-old, Sirt1-KO, 60% diet	20-week-old, Sirt1-KO, *ad libitum*	skeletal muscle	*Myom2*	0.34	10^{-2}	[61]
233	*Myom2*	−1.63	0.05	mice	20-week-old, Sirt1-KO, 60% diet	20-week-old, Sirt1-KO, *ad libitum*	skeletal muscle	*Myom1*	0.65	10^{-3}	[61]
234	*Myom2*	−1.63	0.05	mice	20-week-old, Sirt1-KO, 60% diet	20-week-old, Sirt1-KO, *ad libitum*	skeletal muscle	*Myom3*	0.56	10^{-2}	[61]
235	*Myom2*	−1.63	0.05	mice	80-week-old, Sirt1-KO, 60% diet	80-week-old, Sirt1-KO, *ad libitum*	skeletal muscle	*Myom3*	0.62	10^{-2}	[61]
236	*Myom2*	−1.63	0.05	mice	20-week-old, Sirt1-KI, 60% diet	20-week-old, Sirt1-KI, *ad libitum*	skeletal muscle	*Myom1*	0.45	10^{-2}	[61]
237	*Myom2*	−1.63	0.05	mice	80-week-old, Sirt1-KI, 60% diet	80-week-old, Sirt1-KI, *ad libitum*	skeletal muscle	*Myom1*	0.46	10^{-3}	[61]
238	*Myom2*	−1.63	0.05	mice	80-week-old, Sirt1-KI, 60% diet	80-week-old, Sirt1-KI, *ad libitum*	skeletal muscle	*Myom3*	−0.57	10^{-2}	[61]
239	*Myom2*	−1.63	0.05	mice	20-week-old, Sirt1-KO, *ad libitum*	20-week-old, wild-type, *ad libitum*	skeletal muscle	*Myom1*	−0.46	0.05	[61]
240	*Myom2*	−1.63	0.05	mice	20-month-old, 2 parabionts	6-month-old, 2 parabionts	aortic arch	*Myom1*	−2.24	0.05	[59]
241	*Nfxl1*	0.69	10^{-2}	rat	ISIAH (hypertensive aged vessels)	WAG (norm)	adrenal gland	*Nfx1*	0.50	0.05	[51]
242	*Nfxl1*	0.69	10^{-2}	rat	20-passage-old	5-passage-old	MSC(BM) cells	*Nfx1*	0.37	10^{-9}	[35]
243	*Nfxl1*	0.69	10^{-2}	mice	80-week-old, Sirt1-KO, *ad libitum*	80-week-old, wild-type, *ad libitum*	skeletal muscle	*Nfxl1*	−0.61	0.05	[61]
244	*Nfxl1*	0.69	10^{-2}	mice	BPH/2l hypertensive	BPN/3J, norm	kidney	*Nfxl1*	−1.24	0.05	[62]
245	*Nfxl1*	0.69	10^{-2}	chicken	456-day-old, subfertility	224-day-old, fertility peak	ovary	*Nfx1*	1.12	10^{-4}	[34]
246	*Nmb*	−3.27	10^{-4}	mice	23-month-old, bone fragility	2-month-old	bone	*Nmb*	0.75	0.05	[55]
247	*Nmb*	−3.27	10^{-4}	mice	80-week-old, wild-type, 60% diet	80-week-old, wild-type, *ad libitum*	skeletal muscle	*Nmb*	1.55	10^{-2}	[61]
248	*Nmb*	−3.27	10^{-4}	mice	20-week-old, wild-type, 60% diet	20-week-old, wild-type, *ad libitum*	skeletal muscle	*Nmb*	−1.21	0.05	[61]
249	*Nmb*	−3.27	10^{-4}	mice	80-week-old, Sirt1-KI, 60% diet	80-week-old, Sirt1-KI, *ad libitum*	skeletal muscle	*Nmb*	1.27	0.05	[61]
250	*Nmnat1*	0.94	10^{-4}	rat	20-passage-old	5-passage-old	MSC(BM) cells	*Nmnat3*	1.20	10^{-11}	[35]

Table S1 (continued).

#	PAG, tame *vs* aggressive rats			DEGs in ARD-susceptible *vs* ARD-resistant animals							
	DEG	log2	PADJ	Species	ARD susceptibility	ARD resistance	Tissue	*DEG*	log2	PADJ	Ref
i	ii	iii	iv	v	vi	vii	viii	ix	x	xi	xii
251	*Nmnat1*	0.94	10^{-4}	mice	30-month-old, bone fragility	2-month-old	bone	*Nmnat1*	−0.81	0.05	[55]
252	*Nmnat1*	0.94	10^{-4}	mice	11-month-old, bone fragility	2-month-old	bone	*Nmnat2*	−1.29	10^{-2}	[55]
253	*Nmnat1*	0.94	10^{-4}	mice	30-month-old, bone fragility	2-month-old	bone	*Nmnat2*	−1.64	10^{-5}	[55]
254	*Nmnat1*	0.94	10^{-4}	mice	20-week-old, Sirt1-KO, 60% diet	20-week-old, Sirt1-KO, *ad libitum*	skeletal muscle	*Nmnat1*	−0.42	0.05	[61]
255	*Nmnat1*	0.94	10^{-4}	mice	80-week-old, Sirt1-KO, *ad libitum*	80-week-old, wild-type, *ad libitum*	skeletal muscle	*Nmnat1*	−0.47	0.05	[61]
256	*Nmnat1*	0.94	10^{-4}	mice	80-week-old, Sirt1-KO, *ad libitum*	80-week-old, wild-type, *ad libitum*	skeletal muscle	*Nmnat3*	−0.57	0.05	[61]
257	*Nmnat1*	0.94	10^{-4}	mice	BPH/2l hypertensive	BPN/3J, norm	kidney	*Nmnat1*	1.31	10^{-3}	[62]
258	*Nmnat1*	0.94	10^{-4}	rabbit	under Goldblatt 2-kidney 1-clip	norm	prefrontal cortex	*Nmnat3*	1.09	10^{-4}	[63]
259	*Pcp2*	−5.91	10^{-2}	rat	20-passage-old	5-passage-old	MSC(BM) cells	*Pcp4*	−2.55	10^{-12}	[35]
260	*Pcp2*	−5.91	10^{-2}	rat	20-passage-old	5-passage-old	MSC(BM) cells	*Pcp4l1*	2.21	10^{-23}	[35]
261	*Pcp2*	−5.91	10^{-2}	mice	80-week-old, Sirt1-KI, 60% diet	80-week-old, Sirt1-KI, *ad libitum*	skeletal muscle	*Pcp4l1*	−0.87	10^{-3}	[61]
262	*Pcp2*	−5.91	10^{-2}	mice	80-week-old, Sirt1-KO, *ad libitum*	80-week-old, wild-type, *ad libitum*	skeletal muscle	*Pcp4l1*	−1.85	0.05	[61]
263	*Pcp2*	−5.91	10^{-2}	chicken	469-day-old, spurt aging by hunger	527-day-old, renewal fasting-diet	ovary	*Pcp4*	−2.15	0.05	[34]
264	*Pdia4*	−0.59	10^{-2}	rat	ISIAH (hypertensive aged vessels)	WAG (norm)	renal cortex	*Pdia5*	0.76	0.05	[50]
265	*Pdia4*	−0.59	10^{-2}	rat	20-passage-old	5-passage-old	MSC(BM) cells	*Pdia4*	−0.13	10^{-3}	[35]
266	*Pdia4*	−0.59	10^{-2}	rat	20-passage-old	5-passage-old	MSC(BM) cells	*Pdia3*	−0.20	10^{-8}	[35]
267	*Pdia4*	−0.59	10^{-2}	rat	20-passage-old	5-passage-old	MSC(BM) cells	*Pdia6*	−0.23	10^{-9}	[35]
268	*Pdia4*	−0.59	10^{-2}	rat	5-passage-old	5-passage-old, Aspirin	MSC(BM) cells	*Pdia4*	0.16	10^{-7}	[35]
269	*Pdia4*	−0.59	10^{-2}	rat	5-passage-old	5-passage-old, Aspirin	MSC(BM) cells	*Pdia3*	0.08	10^{-2}	[35]
270	*Pdia4*	−0.59	10^{-2}	mice	80-week-old, wild-type, 60% diet	80-week-old, wild-type, *ad libitum*	skeletal muscle	*Pdia4*	−0.66	0.05	[61]
271	*Pdia4*	−0.59	10^{-2}	mice	20-week-old, Sirt1-KO, 60% diet	20-week-old, Sirt1-KO, *ad libitum*	skeletal muscle	*Pdia3*	−0.47	10^{-2}	[61]
272	*Pdia4*	−0.59	10^{-2}	mice	20-week-old, Sirt1-KI, 60% diet	20-week-old, Sirt1-KI, *ad libitum*	skeletal muscle	*Pdia3*	−0.61	10^{-2}	[61]
273	*Pdia4*	−0.59	10^{-2}	mice	80-week-old, Sirt1-KI, 60% diet	80-week-old, Sirt1-KI, *ad libitum*	skeletal muscle	*Pdia3*	−0.26	0.05	[61]
274	*Pdia4*	−0.59	10^{-2}	mice	BPH/2l hypertensive	BPN/3J, norm	kidney	*Pdia6*	−1.25	10^{-3}	[62]

Table S1 (continued).

#	PAG, tame *vs* aggressive rats			DEGs in ARD-susceptible *vs* ARD-resistant animals							
	DEG	**log2₂**	**P$_{ADJ}$**	**Species**	**ARD susceptibility**	**ARD resistance**	**Tissue**	***DEG***	**log2₂**	P$_{ADJ}$	Ref
i	ii	iii	iv	v	vi	vii	viii	ix	x	xi	xii
275	*Pdia4*	−0.59	10^{-2}	mice	11-month-old, bone fragility	2-month-old	bone	*Pdia5*	−0.90	0.05	[55]
276	*Pdia4*	−0.59	10^{-2}	chicken	469-day-old, spurt aging by hunger	456-day-old, subfertility	ovary	*Pdia2*	−1.39	0.05	[34]
277	*Pla2g2c*	−1.60	10^{-4}	rat	OXYS : spurred aging, 20-day-old	Wistar, 20-day-old	hippocampus	*Pla2g2d*	0.87	10^{-2}	[44]
278	*Pla2g2c*	−1.60	10^{-4}	rat	OXYS : spurred aging, 5-month-old	Wistar, 5-month-old	hippocampus	*Pla2g2d*	1.16	10^{-4}	[44]
279	*Pla2g2c*	−1.60	10^{-4}	rat	OXYS : spurred aging, 20-day-old	Wistar, 20-day-old	hippocampus	*Pla2g5*	1.22	10^{-3}	[44]
280	*Pla2g2c*	−1.60	10^{-4}	rat	ISIAH (hypertensive aged vessels)	WAG (norm)	brain stem	*Pla2g3*	−0.84	10^{-2}	[47]
281	*Pla2g2c*	−1.60	10^{-4}	rat	ISIAH (hypertensive aged vessels)	WAG (norm)	renal medulla	*Pla2g4a*	−0.69	10^{-2}	[49]
282	*Pla2g2c*	−1.60	10^{-4}	rat	ISIAH (hypertensive aged vessels)	WAG (norm)	renal cortex	*Pla2g16a*	0.58	0.05	[50]
283	*Pla2g2c*	−1.60	10^{-4}	rat	ISIAH (hypertensive aged vessels)	WAG (norm)	renal cortex	*Pla2g7a*	0.71	0.05	[50]
284	*Pla2g2c*	−1.60	10^{-4}	rat	ISIAH (hypertensive aged vessels)	WAG (norm)	adrenal gland	*Pla2g16*	0.62	10^{-2}	[51]
285	*Pla2g2c*	−1.60	10^{-4}	rat	ISIAH (hypertensive aged vessels)	WAG (norm)	adrenal gland	*Pla2g2d*	2.49	10^{-2}	[51]
286	*Pla2g2c*	−1.60	10^{-4}	rat	ISIAH (hypertensive aged vessels)	WAG (norm)	adrenal gland	*Pla2g7*	−0.94	10^{-2}	[51]
287	*Pla2g2c*	−1.60	10^{-4}	rat	SHR (hypertensive aged vessels)	Wistar (norm)	brain pericytes	*Pla2g2c*	4.74	0.05	[52]
288	*Pla2g2c*	−1.60	10^{-4}	rat	20-passage-old	5-passage-old	MSC(BM) cells	*Pla2g12a*	0.79	10^{-7}	[35]
289	*Pla2g2c*	−1.60	10^{-4}	rat	20-passage-old	5-passage-old	MSC(BM) cells	*Pla2g16*	−0.55	10^{-6}	[35]
290	*Pla2g2c*	−1.60	10^{-4}	rat	20-passage-old	5-passage-old	MSC(BM) cells	*Pla2g2e*	−1.51	10^{-3}	[35]
291	*Pla2g2c*	−1.60	10^{-4}	rat	20-passage-old	5-passage-old	MSC(BM) cells	*Pla2g4a*	0.24	10^{-2}	[35]
292	*Pla2g2c*	−1.60	10^{-4}	rat	20-passage-old	5-passage-old	MSC(BM) cells	*Pla2g4d*	2.53	10^{-2}	[35]
293	*Pla2g2c*	−1.60	10^{-4}	rat	20-passage-old	5-passage-old	MSC(BM) cells	*Pla2g4f*	1.88	10^{-6}	[35]
294	*Pla2g2c*	−1.60	10^{-4}	rat	20-passage-old	5-passage-old	MSC(BM) cells	*Pla2g5*	−3.53	10^{-11}	[35]
295	*Pla2g2c*	−1.60	10^{-4}	rat	20-passage-old	5-passage-old	MSC(BM) cells	*Pla2g6*	0.42	10^{-7}	[35]
296	*Pla2g2c*	−1.60	10^{-4}	rat	20-passage-old	5-passage-old	MSC(BM) cells	*Pla2g7*	−2.08	10^{-20}	[35]
297	*Pla2g2c*	−1.60	10^{-4}	rat	5-passage-old	5-passage-old, Aspirin	MSC(BM) cells	*Pla2g15*	−0.26	0.05	[35]
298	*Pla2g2c*	−1.60	10^{-4}	rat	5-passage-old	5-passage-old, Aspirin	MSC(BM) cells	*Pla2g2a*	0580	0.05	[35]
299	*Pla2g2c*	−1.60	10^{-4}	rat	5-passage-old	5-passage-old, Aspirin	MSC(BM) cells	*Pla2g7*	−0.39	0.05	[35]
300	*Pla2g2c*	−1.60	10^{-4}	mice	8h/8h-biorhythm (autistic-like aging)	norm	hippocampus	*Pla2g5*	1.73	0.05	[60]

Table S1 (continued).

#	PAG, tame *vs* aggressive rats			DEGs in ARD-susceptible *vs* ARD-resistant animals							
	DEG	**log2$_2$**	P_{ADJ}	**Species**	**ARD susceptibility**	**ARD resistance**	**Tissue**	***DEG***	**log2$_2$**	P_{ADJ}	**Ref**
i	ii	iii	iv	v	vi	vii	viii	ix	x	xi	xii
301	*Pla2g2c*	−1.60	10^{-4}	mice	11-month-old, bone fragility	2-month-old	bone	*Pla2g5*	−1.34	10^{-2}	[55]
302	*Pla2g2c*	−1.60	10^{-4}	mice	23-month-old, bone fragility	2-month-old	bone	*Pla2g5*	−0.96	10^{-3}	[55]
303	*Pla2g2c*	−1.60	10^{-4}	mice	23-month-old, bone fragility	2-month-old	bone	*Pla2g2d*	1.01	0.05	[55]
304	*Pla2g2c*	−1.60	10^{-4}	mice	30-month-old, bone fragility	2-month-old	bone	*Pla2g5*	−1.05	0.05	[55]
305	*Pla2g2c*	−1.60	10^{-4}	mice	30-month-old, bone fragility	2-month-old	bone	*Pla2g2d*	2.00	10^{-4}	[55]
306	*Pla2g2c*	−1.60	10^{-4}	mice	PolG D257A, cardiac disorder	wild-type norm	heart right ventricle	*Pla2g2d*	−1.77	0.05	[58]
307	*Pla2g2c*	−1.60	10^{-4}	mice	PolG D257A, cardiac disorder	wild-type norm	heart right ventricle	*Pla2g5*	−1.12	10^{-3}	[58]
308	*Pla2g2c*	−1.60	10^{-4}	mice	20-week-old, wild-type, 60% diet	20-week-old, wild-type, *ad libitum*	skeletal muscle	*Pla2g16*	0.42	0.05	[61]
309	*Pla2g2c*	−1.60	10^{-4}	mice	20-week-old, wild-type, 60% diet	20-week-old, wild-type, *ad libitum*	skeletal muscle	*Pla2g5*	−2.31	10^{-4}	[61]
310	*Pla2g2c*	−1.60	10^{-4}	mice	20-week-old, wild-type, 60% diet	20-week-old, wild-type, *ad libitum*	skeletal muscle	*Pla2g6*	0.41	0.05	[61]
311	*Pla2g2c*	−1.60	10^{-4}	mice	20-week-old, wild-type, 60% diet	20-week-old, wild-type, *ad libitum*	skeletal muscle	*Pla2g7*	0.72	10^{-2}	[61]
312	*Pla2g2c*	−1.60	10^{-4}	mice	20-week-old, Sirt1-KO, 60% diet	20-week-old, Sirt1-KO, *ad libitum*	skeletal muscle	*Pla2g12a*	0.48	10^{-2}	[61]
313	*Pla2g2c*	−1.60	10^{-4}	mice	20-week-old, Sirt1-KO, 60% diet	20-week-old, Sirt1-KO, *ad libitum*	skeletal muscle	*Pla2g4e*	0.26	0.05	[61]
314	*Pla2g2c*	−1.60	10^{-4}	mice	20-week-old, Sirt1-KO, 60% diet	20-week-old, Sirt1-KO, *ad libitum*	skeletal muscle	*Pla2g7c*	1.29	10^{-3}	[61]
315	*Pla2g2c*	−1.60	10^{-4}	mice	80-week-old, Sirt1-KO, 60% diet	80-week-old, Sirt1-KO, *ad libitum*	skeletal muscle	*Pla2g7c*	−1.50	10^{-2}	[61]
316	*Pla2g2c*	−1.60	10^{-4}	mice	20-week-old, Sirt1-KI, 60% diet	20-week-old, Sirt1-KI, *ad libitum*	skeletal muscle	*Pla2g16c*	−0.35	10^{-2}	[61]
317	*Pla2g2c*	−1.60	10^{-4}	mice	80-week-old, Sirt1-KI, 60% diet	80-week-old, Sirt1-KI, *ad libitum*	skeletal muscle	*Pla2g16c*	0.32	0.05	[61]
318	*Pla2g2c*	−1.60	10^{-4}	mice	80-week-old, Sirt1-KI, 60% diet	80-week-old, Sirt1-KI, *ad libitum*	skeletal muscle	*Pla2g4e*	0.34	10^{-2}	[61]
319	*Pla2g2c*	−1.60	10^{-4}	mice	80-week-old, Sirt1-KI, 60% diet	80-week-old, Sirt1-KI, *ad libitum*	skeletal muscle	*Pla2g5*	−2.50	10^{-3}	[61]
320	*Pla2g2c*	−1.60	10^{-4}	mice	80-week-old, Sirt1-KI, 60% diet	80-week-old, Sirt1-KI, *ad libitum*	skeletal muscle	*Pla2g7*	1.62	10^{-2}	[61]
321	*Pla2g2c*	−1.60	10^{-4}	mice	20-week-old, Sirt1-KO, *ad libitum*	20-week-old, wild-type, *ad libitum*	skeletal muscle	*Pla2g7*	−2.25	10^{-4}	[61]
322	*Pla2g2c*	−1.60	10^{-4}	mice	80-week-old, wild-type, *ad libitum*	80-week-old, Sirt1-KI, *ad libitum*	skeletal muscle	*Pla2g5*	−3.53	0.05	[61]

Table S1 (continued).

#	PAG, tame *vs* aggressive rats			DEGs in ARD-susceptible *vs* ARD-resistant animals							
	DEG	**log2₂**	P_{ADJ}	**Species**	**ARD susceptibility**	**ARD resistance**	**Tissue**	***DEG***	**log2₂**	P_{ADJ}	**Ref**
i	ii	iii	iv	v	vi	vii	viii	ix	x	xi	xii
323	*Pla2g2c*	−1.60	10^{-4}	mice	20-week-old, wild-type, *ad libitum*	20-week-old, Sirt1-KI, *ad libitum*	skeletal muscle	*Pla2g5*	−0.70	0.05	[61]
324	*Pla2g2c*	−1.60	10^{-4}	mice	BPH/2l hypertensive	BPN/3J, norm	kidney	*Pla2g5*	2.59	10^{-6}	[62]
325	*Pla2g2c*	−1.60	10^{-4}	rabbit	under Goldblatt 2-kidney 1-clip	norm	prefrontal cortex	*Pla2g1b*	1.26	10^{-4}	[63]
326	*Pla2g2c*	−1.60	10^{-4}	chicken	469-day-old, spurt aging by hunger	456-day-old, subfertility	ovary	*Pla2g3*	−1.73	10^{-2}	[34]
327	*Pla2g2c*	−1.60	10^{-4}	chicken	469-day-old, spurt aging by hunger	527-day-old, renewal fasting-diet	ovary	*Pla2g4b*	−1.85	0.05	[34]
328	*Plod1*	−0.85	10^{-2}	rat	ISIAH (hypertensive aged vessels)	WAG (norm)	adrenal gland	*Plod1*	−0.98	10^{-2}	[51]
329	*Plod1*	−0.85	10^{-2}	rat	ISIAH (hypertensive aged vessels)	WAG (norm)	hypothalamus	*Plod3*	1.14	0.05	[48]
330	*Plod1*	−0.85	10^{-2}	rat	5-passage-old	5-passage-old, Aspirin	MSC(BM) cells	*Plod1*	−0.20	10^{-10}	[35]
331	*Plod1*	−0.85	10^{-2}	rat	5-passage-old	5-passage-old, Aspirin	MSC(BM) cells	*Plod2*	−0.08	0.05	[35]
332	*Plod1*	−0.85	10^{-2}	rat	5-passage-old	5-passage-old, Aspirin	MSC(BM) cells	*Plod3*	−0.21	10^{-8}	[35]
333	*Plod1*	−0.85	10^{-2}	mice	11-month-old, bone fragility	2-month-old	bone	*Plod1*	−0.93	10^{-3}	[55]
334	*Plod1*	−0.85	10^{-2}	mice	11-month-old, bone fragility	2-month-old	bone	*Plod2*	−1.81	10^{-4}	[55]
335	*Plod1*	−0.85	10^{-2}	mice	23-month-old, bone fragility	2-month-old	bone	*Plod1*	−0.64	10^{-6}	[55]
336	*Plod1*	−0.85	10^{-2}	mice	23-month-old, bone fragility	2-month-old	bone	*Plod2*	−0.92	0.05	[55]
337	*Plod1*	−0.85	10^{-2}	mice	30-month-old, bone fragility	2-month-old	bone	*Plod1*	−0.68	10^{-8}	[55]
338	*Plod1*	−0.85	10^{-2}	mice	30-month-old, bone fragility	2-month-old	bone	*Plod2*	−1.19	10^{-2}	[55]
339	*Plod1*	−0.85	10^{-2}	mice	80-week-old, Sirt1-KI, 60% diet	80-week-old, Sirt1-KI, *ad libitum*	skeletal muscle	*Plod1*	−0.30	0.05	[61]
340	*Plod1*	−0.85	10^{-2}	mice	80-week-old, Sirt1-KI, 60% diet	80-week-old, Sirt1-KI, *ad libitum*	skeletal muscle	*Plod2*	−0.96	10^{-2}	[61]
341	*Plod1*	−0.85	10^{-2}	mice	20-week-old, wild-type, 60% diet	20-week-old, wild-type, *ad libitum*	skeletal muscle	*Plod2*	−1.56	10^{-3}	[61]
342	*Plod1*	−0.85	10^{-2}	mice	20-week-old, Sirt1-KI, 60% diet	20-week-old, Sirt1-KI, *ad libitum*	skeletal muscle	*Plod2*	−1.01	0.05	[61]
343	*Plod1*	−0.85	10^{-2}	mice	80-week-old, Sirt1-KO, 60% diet	80-week-old, Sirt1-KO, *ad libitum*	skeletal muscle	*Plod3*	−0.54	0.05	[61]
344	*Plod1*	−0.85	10^{-2}	mice	BPH/2l hypertensive	BPN/3J, norm	kidney	*Plod2*	1.53	10^{-3}	[62]
345	*Pter*	1.27	0.05	rat	ISIAH (hypertensive aged vessels)	WAG (norm)	adrenal gland	*Pter*	0.96	10^{-2}	[51]
346	*Pter*	1.27	0.05	rat	20-passage-old	5-passage-old	MSC(BM) cells	*Pter*	−0.90	10^{-18}	[35]
347	*Pter*	1.27	0.05	mice	24-month-old, male	3-month-old, male	kidney	*Pter*	−3.52	10^{-2}	[57]
348	*Pter*	1.27	0.05	mice	80-week-old, wild-type, *ad libitum*	80-week-old, Sirt1-KI, *ad libitum*	skeletal muscle	*Pter*	−0.53	0.05	[61]
349	*Pygl*	−0.95	0.05	rat	20-passage-old	20-passage-old, Aspirin	MSC(BM) cells	*Pygl*	0.19	0.05	[35]
350	*Pygl*	−0.95	0.05	rat	20-passage-old	5-passage-old	MSC(BM) cells	*Pygl*	−0.30	10^{-9}	[35]

Table S1 (continued).

#	PAG, tame *vs* aggressive rats			DEGs in ARD-susceptible *vs* ARD-resistant animals							
	DEG	log2₂	P_{ADJ}	Species	ARD susceptibility	ARD resistance	Tissue	*DEG*	log2₂	P_{ADJ}	Ref
i	ii	iii	iv	v	vi	vii	viii	ix	x	xi	xii
351	*Pygl*	−0.95	0.05	rat	20-passage-old	5-passage-old	MSC(BM) cells	*Pygb*	0.91	10^{-80}	[35]
352	*Pygl*	−0.95	0.05	rat	20-passage-old	5-passage-old	MSC(BM) cells	*Pygm*	1.18	10^{-5}	[35]
353	*Pygl*	−0.95	0.05	mice	30-month-old, bone fragility	2-month-old	bone	*Pygl*	0.99	10^{-3}	[55]
354	*Pygl*	−0.95	0.05	mice	30-month-old, bone fragility	2-month-old	bone	*Pygm*	−1.18	0.05	[55]
355	*Pygl*	−0.95	0.05	mice	20-week-old, Sirt1-KO, 60% diet	20-week-old, Sirt1-KO, *ad libitum*	skeletal muscle	*Pygl*	1.34	10^{-2}	[61]
356	*Pygl*	−0.95	0.05	mice	20-week-old, Sirt1-KI, 60% diet	20-week-old, Sirt1-KI, *ad libitum*	skeletal muscle	*Pygb*	0.54	0.05	[61]
357	*Pygl*	−0.95	0.05	mice	27-month-old, male	2-month-old, male	kidney	*Pygm*	−2.44	10^{-4}	[56]
358	*Rbm3*	1.22	10^{-4}	rat	OXYS : spurred aging, 18-month-old	Wistar, 18-month-old	prefrontal cortex	*Rbm3*	−0.35	0.05	[45]
359	*Rbm3*	1.22	10^{-4}	rat	ISIAH (hypertensive aged vessels)	WAG (norm)	renal cortex	*Rbm3*	0.49	0.05	[50]
360	*Rbm3*	1.22	10^{-4}	rat	20-passage-old	5-passage-old	MSC(BM) cells	*Rbm3*	0.11	10^{-2}	[35]
361	*Rbm3*	1.22	10^{-4}	rat	20-passage-old	5-passage-old	MSC(BM) cells	*Rbm19*	−0.38	10^{-3}	[35]
362	*Rbm3*	1.22	10^{-4}	rat	20-passage-old	5-passage-old	MSC(BM) cells	*Rbm39*	0.62	10^{-70}	[35]
363	*Rbm3*	1.22	10^{-4}	rat	20-passage-old	5-passage-old	MSC(BM) cells	*Rbm4*	−0.26	10^{-2}	[35]
364	*Rbm3*	1.22	10^{-4}	rat	20-passage-old	5-passage-old	MSC(BM) cells	*Rbm4b*	0.33	10^{-2}	[35]
365	*Rbm3*	1.22	10^{-4}	rat	20-passage-old	5-passage-old	MSC(BM) cells	*Rbmx*	0.48	10^{-18}	[35]
366	*Rbm3*	1.22	10^{-4}	rat	5-passage-old	5-passage-old, Aspirin	MSC(BM) cells	*Rbm3*	0.26	10^{-15}	[35]
367	*Rbm3*	1.22	10^{-4}	rat	20-passage-old	20-passage-old, Aspirin	MSC(BM) cells	*Rbm3*	0.13	0.05	[35]
368	*Rbm3*	1.22	10^{-4}	mice	24-month-old, male	3-month-old, male	kidney	*Rbm3*	1.49	0.05	[57]
369	*Rbm3*	1.22	10^{-4}	mice	BPH/2l hypertensive	BPN/3J, norm	kidney	*Rbm39*	−1.61	0.05	[62]
370	*Rbm3*	1.22	10^{-4}	mice	BPH/2l hypertensive	BPN/3J, norm	kidney	*Rbm4b*	−1.61	10^{-4}	[62]
371	*Rbm3*	1.22	10^{-4}	mice	80-week-old, wild-type, 60% diet	80-week-old, wild-type, *ad libitum*	skeletal muscle	*Rbm3*	1.53	10^{-2}	[61]
372	*Rbm3*	1.22	10^{-4}	mice	20-week-old, wild-type, 60% diet	20-week-old, wild-type, *ad libitum*	skeletal muscle	*Rbm3*	0.84	10^{-2}	[61]
373	*Rbm3*	1.22	10^{-4}	mice	80-week-old, Sirt1-KO, *ad libitum*	80-week-old, wild-type, *ad libitum*	skeletal muscle	*Rbm3*	−1.48	10^{-2}	[61]
374	*Rbm3*	1.22	10^{-4}	mice	20-week-old, Sirt1-KO, *ad libitum*	20-week-old, wild-type, *ad libitum*	skeletal muscle	*Rbm3*	0.95	0.05	[61]
375	*Rbm3*	1.22	10^{-4}	mice	20-week-old, Sirt1-KI, 60% diet	20-week-old, Sirt1-KI, *ad libitum*	skeletal muscle	*Rbm3*	0.47	10^{-3}	[61]
376	*Rbm3*	1.22	10^{-4}	mice	20-week-old, Sirt1-KI, 60% diet	20-week-old, Sirt1-KI, *ad libitum*	skeletal muscle	*Rbmx*	0.41	0.05	[61]

Table S1 (continued).

#	PAG, tame *vs* aggressive rats			DEGs in ARD-susceptible *vs* ARD-resistant animals							
	DEG	log2	PADJ	Species	ARD susceptibility	ARD resistance	Tissue	*DEG*	log2	PADJ	Ref
i	ii	iii	iv	v	vi	vii	viii	ix	x	xi	xii
377	*Rbm3*	1.22	10^{-4}	mice	80-week-old, Sirt1-KI, 60% diet	80-week-old, Sirt1-KI, *ad libitum*	skeletal muscle	*Rbm3*	0.99	10^{-4}	[61]
378	*Rbm3*	1.22	10^{-4}	mice	80-week-old, Sirt1-KI, 60% diet	80-week-old, Sirt1-KI, *ad libitum*	skeletal muscle	*Rbm19*	0.28	0.05	[61]
379	*Rbm3*	1.22	10^{-4}	mice	80-week-old, Sirt1-KI, 60% diet	80-week-old, Sirt1-KI, *ad libitum*	skeletal muscle	*Rbmx*	0.27	10^{-2}	[61]
380	*Rbm3*	1.22	10^{-4}	mice	80-week-old, Sirt1-KO, 60% diet	80-week-old, Sirt1-KO, *ad libitum*	skeletal muscle	*Rbm3*	1.46	10^{-3}	[61]
381	*Rbm3*	1.22	10^{-4}	mice	80-week-old, Sirt1-KO, 60% diet	80-week-old, Sirt1-KO, *ad libitum*	skeletal muscle	*Rbmx*	0.60	0.05	[61]
382	*Rbm3*	1.22	10^{-4}	mice	20-week-old, Sirt1-KO, 60% diet	20-week-old, Sirt1-KO, *ad libitum*	skeletal muscle	*Rbm19*	0.58	0.05	[61]
383	*Rbm3*	1.22	10^{-4}	mice	30-month-old, bone fragility	2-month-old	bone	*Rbm47*	1.09	10^{-3}	[55]
384	*Rbm3*	1.22	10^{-4}	mice	8h/8h-biorhythm (autistic-like aging)	norm	hippocampus	*Rbm47*	1.41	0.05	[60]
385	*Retsat*	−1.01	0.05	rat	OXYS : spurred aging, 18-month-old	Wistar, 18-month-old	retina	*Retsat*	−0.97	0.05	[46]
386	*Retsat*	−1.01	0.05	rat	ISIAH (hypertensive aged vessels)	WAG (norm)	brain stem	*Retsat*	1.86	10^{-2}	[47]
387	*Retsat*	−1.01	0.05	rat	ISIAH (hypertensive aged vessels)	WAG (norm)	hypothalamus	*Retsat*	1.82	10^{-2}	[48]
388	*Retsat*	−1.01	0.05	rat	ISIAH (hypertensive aged vessels)	WAG (norm)	renal cortex	*Retsat*	1.85	0.05	[50]
389	*Retsat*	−1.01	0.05	rat	ISIAH (hypertensive aged vessels)	WAG (norm)	renal medulla	*Retsat*	2.05	10^{-2}	[49]
390	*Retsat*	−1.01	0.05	rat	ISIAH (hypertensive aged vessels)	WAG (norm)	adrenal gland	*Retsat*	2.59	10^{-2}	[51]
391	*Retsat*	−1.01	0.05	rat	20-passage-old	5-passage-old	MSC(BM) cells	*Retsat*	0.70	10^{-37}	[35]
392	*Retsat*	−1.01	0.05	rat	20-passage-old	20-passage-old, Aspirin	MSC(BM) cells	*Retsat*	−0.22	10^{-3}	[35]
393	*Retsat*	−1.01	0.05	mice	80-week-old, Sirt1-KO, 60% diet	80-week-old, Sirt1-KO, *ad libitum*	skeletal muscle	*Retsat*	0.65	10^{-2}	[61]
394	*Retsat*	−1.01	0.05	chicken	469-day-old, spurt aging by hunger	527-day-old, renewal fasting-diet	ovary	*Retsatl*	−2.20	10^{-2}	[34]
395	*Rhobtb3*	0.86	0.05	rat	ISIAH (hypertensive aged vessels)	WAG (norm)	brain stem	*Rhobtb3*	−0.68	0.05	[47]
396	*Rhobtb3*	0.86	0.05	rat	ISIAH (hypertensive aged vessels)	WAG (norm)	adrenal gland	*Rhobtb3*	−0.77	10^{-2}	[51]
397	*Rhobtb3*	0.86	0.05	rat	ISIAH (hypertensive aged vessels)	WAG (norm)	adrenal gland	*Rhobtb1*	−0.57	0.05	[51]
398	*Rhobtb3*	0.86	0.05	rat	20-passage-old	5-passage-old	MSC(BM) cells	*Rhobtb3*	−0.45	10^{-11}	[35]
399	*Rhobtb3*	0.86	0.05	rat	20-passage-old	5-passage-old	MSC(BM) cells	*Rhobtb1*	−0.20	0.05	[35]
400	*Rhobtb3*	0.86	0.05	rat	20-passage-old	5-passage-old	MSC(BM) cells	*Rhobtb2*	0.27	10^{-2}	[35]

Table S1 (continued).

#	PAG, tame *vs* aggressive rats			DEGs in ARD-susceptible *vs* ARD-resistant animals							
	DEG	**log2**	**PADJ**	**Species**	**ARD susceptibility**	**ARD resistance**	**Tissue**	***DEG***	**log2**	PADJ	Ref
i	ii	iii	iv	v	vi	vii	viii	ix	x	xi	xii
401	*Rhobtb3*	0.86	0.05	mice	30-month-old, bone fragility	2-month-old	bone	*Rhobtb3*	−0.57	10^{-2}	[55]
402	*Rhobtb3*	0.86	0.05	mice	80-week-old, Sirt1-KO, 60% diet	80-week-old, Sirt1-KO, *ad libitum*	skeletal muscle	*Rhobtb3*	−0.30	0.05	[61]
403	*Rhobtb3*	0.86	0.05	mice	80-week-old, wild-type, *ad libitum*	80-week-old, Sirt1-KI, *ad libitum*	skeletal muscle	*Rhobtb3*	−0.55	0.05	[61]
404	*Rhobtb3*	0.86	0.05	mice	80-week-old, wild-type, *ad libitum*	80-week-old, Sirt1-KI, *ad libitum*	skeletal muscle	*Rhobtb1*	−1.71	10^{-4}	[61]
405	*Rhobtb3*	0.86	0.05	mice	80-week-old, wild-type, 60% diet	80-week-old, wild-type, *ad libitum*	skeletal muscle	*Rhobtb1*	−0.90	0.05	[61]
406	*Rhobtb3*	0.86	0.05	mice	20-week-old, wild-type, 60% diet	20-week-old, wild-type, *ad libitum*	skeletal muscle	*Rhobtb1*	−0.56	0.05	[61]
407	*Rhobtb3*	0.86	0.05	mice	20-week-old, wild-type, 60% diet	20-week-old, wild-type, *ad libitum*	skeletal muscle	*Rhobtb2*	0.59	10^{-2}	[61]
408	*Rhobtb3*	0.86	0.05	mice	20-week-old, Sirt1-KO, 60% diet	20-week-old, Sirt1-KO, *ad libitum*	skeletal muscle	*Rhobtb1*	−0.80	0.05	[61]
409	*Rhobtb3*	0.86	0.05	mice	20-week-old, Sirt1-KI, 60% diet	20-week-old, Sirt1-KI, *ad libitum*	skeletal muscle	*Rhobtb1*	−1.22	10^{-3}	[61]
410	*Rhobtb3*	0.86	0.05	mice	80-week-old, Sirt1-KI, 60% diet	80-week-old, Sirt1-KI, *ad libitum*	skeletal muscle	*Rhobtb1*	−1.25	10^{-5}	[61]
411	*Rhobtb3*	0.86	0.05	mice	BPH/2l hypertensive	BPN/3J, norm	kidney	*Rhobtb2*	1.34	10^{-2}	[62]
412	*Rhobtb3*	0.86	0.05	chicken	469-day-old, spurt aging by hunger	527-day-old, renewal fasting-diet	ovary	*Rhob*	−1.69	10^{-3}	[34]
413	*Rln3*	−3.73	10^{-3}	mice	8h/8h-biorhythm (autistic-like aging)	norm	hippocampus	*Rln1*	1.26	0.05	[60]
414	*Scel*	1.24	0.05	rat	20-passage-old	5-passage-old	MSC(BM) cells	*Scel*	−1.22	10^{-7}	[35]
415	*Scel*	1.24	0.05	mice	23-month-old, bone fragility	2-month-old	bone	*Scel*	1.06	0.05	[55]
416	*Scel*	1.24	0.05	mice	30-month-old, bone fragility	2-month-old	bone	*Scel*	1.39	0.05	[55]
417	*Scel*	1.24	0.05	mice	27-month-old	2-month-old	kidney	*Scel*	−1.29	10^{-2}	[56]
418	*Scel*	1.24	0.05	mice	27-month-old, male	2-month-old, male	kidney	*Scel*	−1.55	10^{-2}	[56]
419	*Slfn13*	1.33	0.05	rat	ISIAH (hypertensive aged vessels)	WAG (norm)	renal medulla	*Slfn13*	0.90	0.05	[49]
420	*Slfn13*	1.33	0.05	rat	ISIAH (hypertensive aged vessels)	WAG (norm)	adrenal gland	*Slfn13*	2.43	10^{-2}	[51]
421	*Slfn13*	1.33	0.05	rat	ISIAH (hypertensive aged vessels)	WAG (norm)	adrenal gland	*Slfn2*	0.66	10^{-2}	[51]
422	*Slfn13*	1.33	0.05	rat	ISIAH (hypertensive aged vessels)	WAG (norm)	adrenal gland	*Slfn3*	0.88	10^{-2}	[51]
423	*Slfn13*	1.33	0.05	rat	ISIAH (hypertensive aged vessels)	WAG (norm)	adrenal gland	*Slfn5*	0.51	0.05	[51]

Table S1 (continued).

#	PAG, tame *vs* aggressive rats			DEGs in ARD-susceptible *vs* ARD-resistant animals							
	DEG	**log2**	**PADJ**	**Species**	**ARD susceptibility**	**ARD resistance**	**Tissue**	***DEG***	**log2**	PADJ	Ref
i	ii	iii	iv	v	vi	vii	viii	ix	x	xi	xii
424	*Slfn13*	1.33	0.05	rat	20-passage-old	20-passage-old, Aspirin	MSC(BM) cells	*Slfn13*	0.13	0.05	[35]
425	*Slfn13*	1.33	0.05	rat	20-passage-old	5-passage-old	MSC(BM) cells	*Slfn13*	1.25	10^{-206}	[35]
426	*Slfn13*	1.33	0.05	rat	20-passage-old	5-passage-old	MSC(BM) cells	*Slfn1*	2.30	10^{-10}	[35]
427	*Slfn13*	1.33	0.05	rat	20-passage-old	5-passage-old	MSC(BM) cells	*Slfn2*	1.35	10^{-73}	[35]
428	*Slfn13*	1.33	0.05	rat	20-passage-old	5-passage-old	MSC(BM) cells	*Slfn3*	−2.38	10^{-2}	[35]
429	*Slfn13*	1.33	0.05	rat	20-passage-old	5-passage-old	MSC(BM) cells	*Slfn4*	−1.69	10^{-125}	[35]
430	*Slfn13*	1.33	0.05	rat	20-passage-old	5-passage-old	MSC(BM) cells	*Slfn5*	0.34	10^{-15}	[35]
431	*Slfn13*	1.33	0.05	mice	30-month-old, bone fragility	2-month-old	bone	*Slfn1*	1.15	10^{-3}	[55]
432	*Slfn13*	1.33	0.05	mice	30-month-old, bone fragility	2-month-old	bone	*Slfn10ps*	1.23	0.05	[55]
433	*Slfn13*	1.33	0.05	mice	30-month-old, bone fragility	2-month-old	bone	*Slfn2*	1.05	10^{-2}	[55]
434	*Slfn13*	1.33	0.05	mice	30-month-old, bone fragility	2-month-old	bone	*Slfn4*	1.28	10^{-4}	[55]
435	*Slfn13*	1.33	0.05	mice	27-month-old, male	2-month-old, male	kidney	*Slfn9*	1.64	0.05	[56]
436	*Slfn13*	1.33	0.05	mice	20-week-old, Sirt1-KI, 60% diet	20-week-old, Sirt1-KI, *ad libitum*	skeletal muscle	*Slfn5*	−0.66	0.05	[61]
437	*Slfn13*	1.33	0.05	mice	80-week-old, Sirt1-KI, 60% diet	80-week-old, Sirt1-KI, *ad libitum*	skeletal muscle	*Slfn5*	−0.42	0.05	[61]
438	*Slfn13*	1.33	0.05	mice	80-week-old, Sirt1-KO, 60% diet	80-week-old, Sirt1-KO, *ad libitum*	skeletal muscle	*Slfn5*	0.53	10^{-2}	[61]
439	*Spint1*	−1.16	10^{-3}	mice	23-month-old, bone fragility	2-month-old	bone	*Spint2*	0.77	10^{-2}	[55]
440	*Spint1*	−1.16	10^{-3}	mice	30-month-old, bone fragility	2-month-old	bone	*Spint2*	1.00	10^{-5}	[55]
441	*Spint1*	−1.16	10^{-3}	mice	80-week-old, Sirt1-KI, 60% diet	80-week-old, Sirt1-KI, *ad libitum*	skeletal muscle	*Spint2*	0.85	0.05	[61]
442	*Spint1*	−1.16	10^{-3}	chicken	469-day-old, spurt aging by hunger	527-day-old, renewal fasting-diet	ovary	*Spint2l0*	−1.12	10^{-7}	[34]
443	*Tnnt1*	2.80	0.05	rat	ISIAH (hypertensive aged vessels)	WAG (norm)	brain stem	*Tnnt1*	−3.35	10^{-2}	[47]
444	*Tnnt1*	2.80	0.05	rat	ISIAH (hypertensive aged vessels)	WAG (norm)	brain stem	*Tnnt2*	−1.21	10^{-2}	[47]
445	*Tnnt1*	2.80	0.05	rat	ISIAH (hypertensive aged vessels)	WAG (norm)	hypothalamus	*Tnnt1*	−4.37	10^{-2}	[48]
446	*Tnnt1*	2.80	0.05	rat	ISIAH (hypertensive aged vessels)	WAG (norm)	hypothalamus	*Tnnt2*	−0.91	10^{-2}	[48]
447	*Tnnt1*	2.80	0.05	rat	20-passage-old	5-passage-old	MSC(BM) cells	*Tnnt3*	2.52	10^{-136}	[35]
448	*Tnnt1*	2.80	0.05	mice	20-week-old, wild-type, 60% diet	20-week-old, wild-type, *ad libitum*	skeletal muscle	*Tnnt1*	−4.32	10^{-2}	[61]
449	*Tnnt1*	2.80	0.05	mice	80-week-old, Sirt1-KO, 60% diet	80-week-old, Sirt1-KO, *ad libitum*	skeletal muscle	*Tnnt1*	1.10	0.05	[61]
450	*Tnnt1*	2.80	0.05	mice	80-week-old, Sirt1-KI, 60% diet	80-week-old, Sirt1-KI, *ad libitum*	skeletal muscle	*Tnnt1*	−2.45	10^{-9}	[61]

Table S1 (continued).

#	PAG, tame *vs* aggressive rats			DEGs in ARD-susceptible *vs* ARD-resistant animals							
	DEG	log_2	P_{ADJ}	Species	ARD susceptibility	ARD resistance	Tissue	*DEG*	log_2	P_{ADJ}	Ref
i	ii	iii	iv	v	vi	vii	viii	ix	x	xi	xii
451	*Tnnt1*	2.80	0.05	mice	20-week-old, Sirt1-KO, 60% diet	20-week-old, Sirt1-KO, *ad libitum*	skeletal muscle	*Tnnt3*	−0.27	0.05	[61]
452	*Tnnt1*	2.80	0.05	mice	80-week-old, Sirt1-KO, *ad libitum*	80-week-old, wild-type, *ad libitum*	skeletal muscle	*Tnnt3*	−0.52	0.05	[61]
453	*Tnnt1*	2.80	0.05	mice	20-week-old, Sirt1-KO, *ad libitum*	20-week-old, wild-type, *ad libitum*	skeletal muscle	*Tnnt3*	0.30	0.05	[61]
454	*Tnnt1*	2.80	0.05	fruit fly	10-day-old, Alzheimer disease-like	0-day-old, just post-eclosion	head	*up (Tnnt2)*	−1.73	10^{-2}	[64]
455	*Ucn*	3.61	0.05	mice	80-week-old, Sirt1-KI, 60% diet	80-week-old, Sirt1-KI, *ad libitum*	skeletal muscle	*Ucn*	0.93	0.05	[61]
456	*Wsb1*	0.95	0.05	rat	20-passage-old	5-passage-old	MSC(BM) cells	*Wsb1*	−1.13	10^{-175}	[35]
457	*Wsb1*	0.95	0.05	mice	24-month-old, male	3-month-old, male	kidney	*Wsb1*	−1.06	0.05	[57]
458	*Wsb1*	0.95	0.05	mice	20-week-old, wild-type, 60% diet	20-week-old, wild-type, *ad libitum*	skeletal muscle	*Wsb2*	0.34	10^{-2}	[61]
459	*Wsb1*	0.95	0.05	mice	80-week-old, Sirt1-KI, 60% diet	80-week-old, Sirt1-KI, *ad libitum*	skeletal muscle	*Wsb2*	0.27	0.05	[61]

Notes. Hereinafter, log_2: the log_2-transformed fold change (i.e., the ratio of tame to aggressive rats' expression levels of a given gene); P_{ADJ}: statistical significance according to Fisher's Z-test with the Benjamini correction for multiple comparisons. OXIS, Wistar, BPH/2J and BPH/3J: laboratory animal strains; MSC(BM): bone marrow-derived mesenchymal stromal cells; Aspirin and fasting: rejuvenators; *PolG*: DNA polymerase γ, catalytic subunit; *Sirt1*: sirtuin 1; KO and KI: knock-out and knock-in, respectively. Genes: *Amy1a*, Amylase α1A*; Aox1*, Aldehyde oxidase 1*; Ascl3*, Achaete-scute family bHLH transcription factor 3*; Banp*, BTG3 associated nuclear protein*; Bdkrb2*, Bradykinin receptor B2*; Cartpt*, Cocaine- and amphetamine-regulated transcript prepropeptide*; Cyp2j10*, Cytochrome P450, family 2, subfamily j, polypeptide 10*; Defb17*, Defensin β17*; Emx2*, Empty spiracles homeobox 2*; Fat2*, FAT atypical cadherin 2*; Fcgr2b*, Fc γ receptor IIb; *Fosb*, AP-1 transcription factor subunit FosB proto-oncogene*; Gpd1*, Glycerol-3-phosphate dehydrogenase 1*; Hbb-b1*, Hemoglobin, β adult major chain*; Hspa1b*, Heat shock protein family A (Hsp70) member 1B*; Itm2a*, Integral membrane protein 2A*; Krt2*, Keratin 2*; Morn1*, MORN repeat-containing 1*; Myom2*, Myomesin 2*; Nfxl1*, Nuclear transcription X-box binding like 1 factor*; Nmb*, Neuromedin B*; Nmnat1*, Nicotinamide nucleotide adenylyltransferase 1*; Pcp2*, Purkinje cell protein 2*; Pdia4*, Protein disulfide isomerase family A member 4*; Pdyn*, Prodynorphin*; Pla2g2c* Phospholipase A2, group IIC; *Plod1*, Procollagen-lysine,2-oxoglutarate 5-dioxygenase 1*; Pter*, Phosphotriesterase-related protein*; Pygl*, Glycogen phosphorylase L*; Rbm3*, RNA-binding motif protein 3*; Retsat*, Retinol saturase*; Rhobtb3*, Rho-related BTB domain-containing protein 3*; Rln3*, Relaxin 3*; Scel*, Sciellin*; Slfn13*, Schlafen family member 13*; Spint1*, Serine peptidase inhibitor, Kunitz type 1*; Tnnt1*, Troponin T type 1 (skeletal, slow)*; Ucn*, Urocortin*; Wsb1*, WD repeat and SOCS box-containing protein 1.

Table S2. Novel PAG-related rat DEGs in comparison with their known homologs in the binary "susceptibility versus resistance" models of human ARDs

#	PAG, tame *vs* aggressive rats			DEGs in patients *vs* healthy volunteers						
	DEG	**log₂**	**P_{ADJ}**	**ARD susceptibility**	**ARD resistance**	**Tissue**	***DEG***	**log₂**	**P_{ADJ}**	**Ref**
1	*Amy1a*	1.97	0.05	pulmonary hypertension aging vessels	norm	lung	*AMY2B*	0.39	10^{-3}	[39]
2	*Amy1a*	1.97	0.05	LPS-stimulated atherogenesis aging vessels	*ARID5B*-KO as atheroprotection	THP1 monocytes	*AMY1B*	0.34	10^{-4}	[43]
3	*Amy1a*	1.97	0.05	LPS-stimulated atherogenesis aging vessels	*ARID5B*-KO as atheroprotection	THP1 monocytes	*AMY1B*	-0.29	10^{-2}	[43]
4	*Aox1*	1.83	0.05	pulmonary hypertension aging vessels	norm	lung	*AOX1*	0.49	0.05	[39]
5	*Ascl3*	2.74	10^{-4}	pulmonary hypertension aging vessels	norm	lung	*ASCL1*	0.09	10^{-3}	
6	*Ascl3*	2.74	10^{-4}	pulmonary hypertension fibrosis aging lung	norm	lung	*ASCL2*	-1.46	10^{-3}	[39]
7	*Banp*	-0.64	0.05	LPS-stimulated atherogenesis aging vessels	*ARID5B*-KO as atheroprotection	THP1 monocytes	*BANP*	-0.72	10^{-8}	[43]
8	*Bdkrb2*	1.62	0.05	pulmonary hypertension aging vessels	norm	lung	*BDKRB2*	0.39	10^{-3}	[39]
9	*Defb17*	6.96	10^{-2}	pulmonary hypertension aging vessels	norm	lung	*DEFB104B*	-1.35	10^{-3}	[39]
10	*Defb17*	6.96	10^{-2}	pulmonary hypertension fibrosis aging lung	norm	lung	*DEFB115*	0.05	0.05	[39]
11	*Defb17*	6.96	10^{-2}	myocardial ischemia as aged heart	norm	peripheral blood	*DEFA4*	2.14	0.05	[41]
12	*Fat2*	-4.96	0.05	male, pulmonary hypertension aging lung	norm	blood	*FAT3*	1.16	10^{-3}	[37]
13	*Fat2*	-4.96	0.05	pulmonary hypertension aging vessels	norm	lung	*FAT1*	-0.28	10^{-2}	[39]
14	*Fat2*	-4.96	0.05	pulmonary hypertension aging vessels	norm	lung	*FAT3*	-0.94	10^{-7}	[39]
15	*Fat2*	-4.96	0.05	pulmonary hypertension aging vessels	norm	lung	*FAT4*	-0.31	10^{-3}	[39]
16	*Fat2*	-4.96	0.05	pulmonary hypertension fibrosis aging lung	norm	lung	*FAT4*	0.99	10^{-2}	[39]
17	*Fcgr2b*	2.01	0.05	pulmonary hypertension aging vessels	norm	lung	*FCGR2A*	0.27	0.05	[39]
18	*Fcgr2b*	2.01	0.05	pulmonary hypertension aging vessels	norm	lung	*FCGR3A*	0.22	0.05	[39]
19	*Fcgr2b*	2.01	0.05	pulmonary hypertension fibrosis aging lung	norm	lung	*FCGR1B*	-1.30	10^{-2}	[39]
20	*Fcgr2b*	2.01	0.05	LPS-stimulated atherogenesis aging vessels	*ARID5B*-KO as atheroprotection	THP1 monocytes	*FCGR1A*	0.41	10^{-4}	[43]
21	*Fcgr2b*	2.01	0.05	LPS-stimulated atherogenesis aging vessels	*ARID5B*-KO as atheroprotection	THP1 monocytes	*FCGR1B*	0.55	10^{-4}	[43]
22	*Fcgr2b*	2.01	0.05	LPS-stimulated atherogenesis aging vessels	*ARID5B*-KO as atheroprotection	THP1 monocytes	*FCGR1C*	0.30	10^{-2}	[43]
23	*Fcgr2b*	2.01	0.05	LPS-stimulated atherogenesis aging vessels	*ARID5B*-KO as atheroprotection	THP1 monocytes	*FCGR2A*	0.49	10^{-5}	[43]
24	*Fosb*	-1.85	0.05	myocardial ischemia as aged heart	norm	peripheral blood	*FOSB*	-1.11	0.05	[41]

Table S2 (continued).

#	PAG, tame *vs* aggressive rats			DEGs in patients *vs* healthy volunteers						
	DEG	**log₂**	**P_{ADJ}**	**ARD susceptibility**	**ARD resistance**	**Tissue**	***DEG***	**log₂**	**P_{ADJ}**	**Ref**
25	*Fosb*	−1.85	0.05	LPS-stimulated atherogenesis aging vessels	*ARID5B*-KO as atheroprotection	THP1 monocytes	*FOSB*	0.80	10^{-5}	[43]
26	*Fosb*	−1.85	0.05	LPS-stimulated atherogenesis aging vessels	*ARID5B*-KO as atheroprotection	THP1 monocytes	*FOSL1*	0.32	10^{-3}	[43]
27	*Fosb*	−1.85	0.05	LPS-stimulated atherogenesis aging vessels	*ARID5B*-KO as atheroprotection	THP1 monocytes	*FOSL2*	0.38	10^{-5}	[43]
28	*Fosb*	−1.85	0.05	renal medullary hypertension aging vessels	norm	renal medulla	*FOS*	1.04	0.05	[36]
29	*Fosb*	−1.85	0.05	pulmonary hypertension aging vessels	norm	lung	*FOS*	−0.53	0.05	[39]
30	*Fosb*	−1.85	0.05	pulmonary hypertension fibrosis aging lung	norm	lung	*FOSL1*	1.56	10^{-2}	[39]
31	*Fosb*	−1.85	0.05	frontotemporal dementia as cognitive ageing	norm	large extracellular vesicle	*FOS*	−2.34	0.05	[42]
32	*Fosb*	−1.85	0.05	before training	post-training renewal	vastus externus	*FOSL2*	−0.21	10^{-2}	[33]
33	*Gpd1*	−0.99	0.05	pulmonary hypertension aging vessels	norm	lung	*GPD1*	−0.53	10^{-4}	[39]
34	*Gpd1*	−0.99	0.05	pulmonary hypertension fibrosis aging lung	norm	lung	*GPD1*	−1.98	10^{-2}	[39]
35	*Gpd1*	−0.99	0.05	LPS-stimulated atherogenesis aging vessels	*ARID5B*-KO as atheroprotection	THP1 monocytes	*GPD1L*	0.19	10^{-3}	[43]
36	*Hbb-b1*	−5.90	10^{-5}	pulmonary hypertension aging vessels	norm	lung	*HBD*	−0.47	0.05	[39]
37	*Hbb-b1*	−5.90	10^{-5}	pulmonary hypertension aging vessels	norm	lung	*HBB*	−2.46	10^{-12}	[39]
38	*Hbb-b1*	−5.90	10^{-5}	pulmonary hypertension aging vessels	norm	lung	*HBG1*	−0.33	0.05	[39]
39	*Hbb-b1*	−5.90	10^{-5}	pulmonary hypertension fibrosis aging lung	norm	lung	*HBD*	−2.83	10^{-3}	[39]
40	*Hbb-b1*	−5.90	10^{-5}	pulmonary hypertension aging vessels	norm	lung	*HBB*	2.46	10^{-10}	[38]
41	*Hbb-b1*	−5.90	10^{-5}	frontotemporal dementia as cognitive ageing	norm	large extracellular vesicle	*HBD*	1.89	0.05	[42]
42	*Hbb-b1*	−5.90	10^{-5}	frontotemporal dementia as cognitive ageing	norm	small extracellular vesicle	*HBB*	−1.33	0.05	[42]
43	*Hbb-b1*	−5.90	10^{-5}	myocardial ischemia as aged heart	norm	peripheral blood	*HBBP1*	1.03	0.05	[41]
44	*Hbb-b1*	−5.90	10^{-5}	myocardial ischemia as aged heart	norm	peripheral blood	*HBE1*	1.42	0.05	[41]
45	*Hspa1b*	−2.14	10^{-7}	LPS-stimulated atherogenesis aging vessels	*ARID5B*-KO as atheroprotection	THP1 monocytes	*HSPA1B*	−0.46	10^{-4}	[43]
46	*Hspa1b*	−2.14	10^{-7}	LPS-stimulated atherogenesis aging vessels	*ARID5B*-KO as atheroprotection	THP1 monocytes	*HSPA6*	−0.36	10^{-2}	[43]
47	*Hspa1b*	−2.14	10^{-7}	pulmonary hypertension fibrosis aging lung	norm	lung	*HSPA12A*	1.53	10^{-4}	[39]
48	*Hspa1b*	−2.14	10^{-7}	pulmonary hypertension fibrosis aging lung	norm	lung	*HSPA4L*	1.73	10^{-2}	[39]
49	*Hspa1b*	−2.14	10^{-7}	pulmonary hypertension aging vessels	norm	lung	*HSPA12A*	−0.15	10^{-3}	[39]
50	*Hspa1b*	−2.14	10^{-7}	pulmonary hypertension aging vessels	norm	lung	*HSPA12B*	0.16	0.05	[39]

Table S2 (continued).

#	PAG, tame *vs* aggressive rats			DEGs in patients *vs* healthy volunteers						
	DEG	**log2**	**PADJ**	**ARD susceptibility**	**ARD resistance**	**Tissue**	***DEG***	**log2**	PADJ	Ref
51	*Hspa1b*	−2.14	10^{-7}	pulmonary hypertension aging vessels	norm	lung	*HSPA5*	0.38	10^{-2}	[39]
52	*Hspa1b*	−2.14	10^{-7}	atrial fibrillation as heart aging	norm	auricle tissue	*HSPA4*	1.33	10^{-2}	[41]
53	*Itm2a*	0.76	10^{-2}	pulmonary hypertension aging vessels	norm	lung	*ITM2A*	−0.51	10^{-3}	[39]
54	*Itm2a*	0.76	10^{-2}	pulmonary hypertension aging vessels	norm	lung	*ITM2C*	−0.44	10^{-3}	[39]
55	*Itm2a*	0.76	10^{-2}	frontotemporal dementia as cognitive ageing	norm	small extracellular vesicle	*ITM2B*	−1.98	10^{-2}	[42]
56	*Itm2a*	0.76	10^{-2}	amyotrophic lateral sclerosis ageing neurons	norm	small extracellular vesicle	*ITM2B*	−2.28	10^{-2}	[42]
57	*Itm2a*	0.76	10^{-2}	amyotrophic lateral sclerosis ageing neurons	norm	large extracellular vesicle	*ITM2B*	−1.09	10^{-2}	[42]
58	*Krt2*	−1.85	10^{-6}	pulmonary hypertension fibrosis aging lung	norm	lung	*KRT32*	−0.95	10^{-2}	[39]
59	*Krt2*	−1.85	10^{-6}	pulmonary hypertension fibrosis aging lung	norm	lung	*KRT33A*	−1.25	10^{-3}	[39]
60	*Krt2*	−1.85	10^{-6}	pulmonary hypertension fibrosis aging lung	norm	lung	*KRT33B*	−0.71	10^{-2}	[39]
61	*Krt2*	−1.85	10^{-6}	pulmonary hypertension fibrosis aging lung	norm	lung	*KRT36*	−0.89	10^{-2}	[39]
62	*Krt2*	−1.85	10^{-6}	pulmonary hypertension fibrosis aging lung	norm	lung	*KRT76*	−1.58	10^{-2}	[39]
63	*Krt2*	−1.85	10^{-6}	pulmonary hypertension fibrosis aging lung	norm	lung	*KRT79*	−1.25	10^{-2}	[39]
64	*Krt2*	−1.85	10^{-6}	pulmonary hypertension fibrosis aging lung	norm	lung	*KRT9*	−1.35	10^{-2}	[39]
65	*Krt2*	−1.85	10^{-6}	pulmonary hypertension aging vessels	norm	lung	*KRT13*	0.11	10^{-3}	[39]
66	*Krt2*	−1.85	10^{-6}	pulmonary hypertension aging vessels	norm	lung	*KRT15*	0.33	0.05	[39]
67	*Krt2*	−1.85	10^{-6}	pulmonary hypertension aging vessels	norm	lung	*KRT17P5*	0.14	10^{-2}	[39]
68	*Krt2*	−1.85	10^{-6}	pulmonary hypertension aging vessels	norm	lung	*KRT19*	0.25	0.05	[39]
69	*Krt2*	−1.85	10^{-6}	pulmonary hypertension aging vessels	norm	lung	*KRT23*	0.12	10^{-2}	[39]
70	*Krt2*	−1.85	10^{-6}	pulmonary hypertension aging vessels	norm	lung	*KRT33A*	−0.05	0.05	[39]
71	*Krt2*	−1.85	10^{-6}	pulmonary hypertension aging vessels	norm	lung	*KRT4*	0.64	10^{-9}	[39]
72	*Krt2*	−1.85	10^{-6}	myocardial ischemia as aged heart	norm	peripheral blood	*KRT1*	2.28	10^{-2}	[41]
73	*Krt2*	−1.85	10^{-6}	myocardial ischemia as aged heart	norm	peripheral blood	*KRT10*	−1.13	0.05	[41]
74	*Krt2*	−1.85	10^{-6}	myocardial ischemia as aged heart	norm	peripheral blood	*KRT23*	2.64	0.05	[41]

Table S2 (continued).

#	PAG, tame *vs* aggressive rats			DEGs in patients *vs* healthy volunteers						
	DEG	**log2**	**PADJ**	**ARD susceptibility**	**ARD resistance**	**Tissue**	***DEG***	**log2**	PADJ	Ref
75	*Krt2*	−1.85	10^{-6}	myocardial ischemia as aged heart	norm	peripheral blood	*KRT33B*	1.10	0.05	[41]
76	*Krt2*	−1.85	10^{-6}	before training	post-training renewal	vastus externus	*KRT10*	−0.21	10^{-3}	[33]
77	*Morn1*	0.75	0.05	myocardial ischemia as aged heart	norm	peripheral blood	*MORN1*	1.09	10^{-2}	[41]
78	*Myom2*	−1.63	0.05	pulmonary hypertension aging vessels	norm	lung	*MYOM1*	−0.11	10^{-2}	[39]
79	*Nfxl1*	0.69	10^{-2}	pulmonary hypertension aging vessels	norm	lung	*NFXL1*	−0.14	0.05	[39]
80	*Nfxl1*	0.69	10^{-2}	LPS-stimulated atherogenesis aging vessels	*ARID5B*-KO as atheroprotection	THP1 monocytes	*NFXL1*	−0.44	10^{-5}	[43]
81	*Nfxl1*	0.69	10^{-2}	LPS-stimulated atherogenesis aging vessels	*ARID5B*-KO as atheroprotection	THP1 monocytes	*NFX1*	−0.30	10^{-3}	[43]
82	*Nmb*	−3.27	10^{-4}	LPS-stimulated atherogenesis aging vessels	*ARID5B*-KO as atheroprotection	THP1 monocytes	*NMB*	0.28	10^{-3}	[43]
83	*Nmnat1*	0.94	10^{-4}	pulmonary hypertension aging vessels	norm	lung	*NMNAT1*	−0.07	10^{-3}	[39]
84	*Pcp2*	−5.91	10^{-2}	pulmonary hypertension fibrosis aging lung	norm	lung	*PCP2*	−1.64	10^{-3}	[39]
85	*Pdia4*	−0.59	10^{-2}	pulmonary hypertension aging vessels	norm	lung	*PDIA4*	0.33	0.05	[39]
86	*Pdia4*	−0.59	10^{-2}	pulmonary hypertension aging vessels	norm	lung	*PDIA6*	0.26	10^{-2}	[39]
87	*Pdia4*	−0.59	10^{-2}	atrial fibrillation as heart aging	norm	auricle tissue	*PDIA6*	1.23	0.05	[41]
88	*Pla2g2c*	−1.60	10^{-4}	pulmonary hypertension fibrosis aging lung	norm	lung	*PLA2G10*	−1.26	10^{-2}	[39]
89	*Pla2g2c*	−1.60	10^{-4}	pulmonary hypertension fibrosis aging lung	norm	lung	*PLA2G12A*	−0.67	10^{-2}	[39]
90	*Pla2g2c*	−1.60	10^{-4}	pulmonary hypertension fibrosis aging lung	norm	lung	*PLA2G12B*	−2.27	10^{-4}	[39]
91	*Pla2g2c*	−1.60	10^{-4}	pulmonary hypertension fibrosis aging lung	norm	lung	*PLA2G15*	−0.95	10^{-2}	[39]
92	*Pla2g2c*	−1.60	10^{-4}	pulmonary hypertension fibrosis aging lung	norm	lung	*PLA2G1B*	−1.74	10^{-2}	[39]
93	*Pla2g2c*	−1.60	10^{-4}	pulmonary hypertension fibrosis aging lung	norm	lung	*PLA2G2D*	−1.06	10^{-2}	[39]
94	*Pla2g2c*	−1.60	10^{-4}	pulmonary hypertension fibrosis aging lung	norm	lung	*PLA2G2F*	−1.20	10^{-2}	[39]
95	*Pla2g2c*	−1.60	10^{-4}	pulmonary hypertension fibrosis aging lung	norm	lung	*PLA2G3*	−1.46	10^{-2}	[39]
96	*Pla2g2c*	−1.60	10^{-4}	pulmonary hypertension fibrosis aging lung	norm	lung	*PLA2G4F*	−1.54	10^{-3}	[39]
97	*Pla2g2c*	−1.60	10^{-4}	pulmonary hypertension aging vessels	norm	lung	*PLA2G12B*	0.13	10^{-2}	[39]
98	*Pla2g2c*	−1.60	10^{-4}	pulmonary hypertension aging vessels	norm	lung	*PLA2G1B*	−0.51	0.05	[39]
99	*Pla2g2c*	−1.60	10^{-4}	pulmonary hypertension aging vessels	norm	lung	*PLA2G4C*	−0.32	10^{-2}	[39]
100	*Pla2g2c*	−1.60	10^{-4}	pulmonary hypertension aging vessels	norm	lung	*PLA2G4E*	0.09	0.05	[39]

Table S2 (continued).

#	PAG, tame *vs* aggressive rats			DEGs in patients *vs* healthy volunteers						
	DEG	**log2**	**PADJ**	**ARD susceptibility**	**ARD resistance**	**Tissue**	***DEG***	**log2**	**PADJ**	**Ref**
101	*Pla2g2c*	−1.60	10^{-4}	pulmonary hypertension aging vessels	norm	lung	*PLA2G5*	−0.27	0.05	[39]
102	*Pla2g2c*	−1.60	10^{-4}	pulmonary hypertension aging vessels	norm	lung	*PLA2G6*	−0.09	0.05	[39]
103	*Pla2g2c*	−1.60	10^{-4}	pulmonary hypertension aging vessels	norm	lung	*PLA2G7*	0.94	10^{-3}	[39]
104	*Pla2g2c*	−1.60	10^{-4}	atrial fibrillation as heart aging	norm	auricle tissue	*PLA2G12B*	−1.29	10^{-2}	[41]
105	*Pla2g2c*	−1.60	10^{-4}	myocardial ischemia as aged heart	norm	peripheral blood	*PLA2G12A*	−1.31	0.05	[41]
106	*Pla2g2c*	−1.60	10^{-4}	myocardial ischemia as aged heart	norm	peripheral blood	*PLA2G7*	−1.18	0.05	[41]
107	*Pla2g2c*	−1.60	10^{-4}	LPS-stimulated atherogenesis aging vessels	*ARID5B*-KO as atheroprotection	THP1 monocytes	*PLA2G4C*	−0.17	10^{-2}	[43]
108	*Pla2g2c*	−1.60	10^{-4}	LPS-stimulated atherogenesis aging vessels	*ARID5B*-KO as atheroprotection	THP1 monocytes	*PLA2G7*	0.59	10^{-41}	[43]
109	*Plod1*	−0.85	10^{-2}	pulmonary hypertension aging vessels	norm	lung	*PLOD1*	0.22	0.05	[39]
110	*Plod1*	−0.85	10^{-2}	LPS-stimulated atherogenesis aging vessels	*ARID5B*-KO as atheroprotection	THP1 monocytes	*PLOD2*	−0.51	10^{-2}	[43]
111	*Pter*	1.27	0.05	pulmonary hypertension aging vessels	norm	lung	*PTER*	0.12	0.05	[39]
112	*Pygl*	−0.95	0.05	pulmonary hypertension aging vessels	norm	lung	*PYGL*	0.64	10^{-10}	[39]
113	*Pygl*	−0.95	0.05	pulmonary hypertension fibrosis aging lung	norm	lung	*PYGB*	−0.82	10^{-2}	[39]
114	*Pygl*	−0.95	0.05	LPS-stimulated atherogenesis aging vessels	*ARID5B*-KO as atheroprotection	THP1 monocytes	*PYGL*	0.28	10^{-3}	[43]
115	*Pygl*	−0.95	0.05	LPS-stimulated atherogenesis aging vessels	*ARID5B*-KO as atheroprotection	THP1 monocytes	*PYGB*	0.29	10^{-4}	[43]
116	*Rbm3*	1.22	10^{-4}	pulmonary hypertension fibrosis aging lung	norm	lung	*RBM3*	0.93	10^{-2}	[39]
117	*Rbm3*	1.22	10^{-4}	pulmonary hypertension fibrosis aging lung	norm	lung	*RBMX2*	0.62	10^{-2}	[39]
118	*Rbm3*	1.22	10^{-4}	pulmonary hypertension aging vessels	norm	lung	*RBM23*	0.11	0.05	[39]
119	*Rbm3*	1.22	10^{-4}	pulmonary hypertension aging vessels	norm	lung	*RBM39*	−0.15	10^{-3}	[39]
120	*Rbm3*	1.22	10^{-4}	pulmonary hypertension aging vessels	norm	lung	*RBM47*	0.21	10^{-3}	[39]
121	*Rbm3*	1.22	10^{-4}	pulmonary hypertension aging vessels	norm	lung	*RBMXL2*	−0.07	10^{-2}	[39]
122	*Rbm3*	1.22	10^{-4}	myocardial ischemia as aged heart	norm	peripheral blood	*RBM39*	−2.13	0.05	[41]
123	*Rbm3*	1.22	10^{-4}	myocardial ischemia as aged heart	norm	peripheral blood	*RBM4*	−2.14	10^{-2}	[41]

Table S2 (continued).

#	PAG, tame *vs* aggressive rats			DEGs in patients *vs* healthy volunteers						
	DEG	**log2**	**PADJ**	**ARD susceptibility**	**ARD resistance**	**Tissue**	***DEG***	**log2**	PADJ	Ref
124	*Rbm3*	1.22	10^{-4}	amyotrophic lateral sclerosis ageing neurons	norm	small extracellular vesicle	*RBM39*	3.36	10^{-3}	[42]
125	*Rbm3*	1.22	10^{-4}	frontotemporal dementia as cognitive ageing	norm	small extracellular vesicle	*RBM39*	2.30	0.05	[42]
126	*Rbm3*	1.22	10^{-4}	LPS-stimulated atherogenesis aging vessels	*ARID5B*-KO as atheroprotection	THP1 monocytes	*RBM4*	−0.27	10^{-2}	[43]
127	*Rbm3*	1.22	10^{-4}	LPS-stimulated atherogenesis aging vessels	*ARID5B*-KO as atheroprotection	THP1 monocytes	*RBMX2*	−0.21	10^{-2}	[43]
128	*Retsat*	−1.01	0.05	pulmonary hypertension fibrosis aging lung	norm	lung	*RETSAT*	−0.83	10^{-3}	[39]
129	*Retsat*	−1.01	0.05	LPS-stimulated atherogenesis aging vessels	*ARID5B*-KO as atheroprotection	THP1 monocytes	*RETSAT*	0.21	10^{-3}	[43]
130	*Rhobtb3*	0.86	0.05	atrial fibrillation as heart aging	norm	auricle tissue	*RHOBTB3*	1.26	10^{-2}	[41]
131	*Rhobtb3*	0.86	0.05	myocardial ischemia as aged heart	norm	peripheral blood	*RHOBTB3*	1.51	10^{-3}	[41]
132	*Rhobtb3*	0.86	0.05	LPS-stimulated atherogenesis aging vessels	*ARID5B*-KO as atheroprotection	THP1 monocytes	*RHOBTB3*	0.43	10^{-4}	[43]
133	*Rhobtb3*	0.86	0.05	LPS-stimulated atherogenesis aging vessels	*ARID5B*-KO as atheroprotection	THP1 monocytes	*RHOB*	0.51	10^{-3}	[43]
134	*Rhobtb3*	0.86	0.05	LPS-stimulated atherogenesis aging vessels	*ARID5B*-KO as atheroprotection	THP1 monocytes	*RHOG*	−0.18	10^{-2}	[43]
135	*Rhobtb3*	0.86	0.05	LPS-stimulated atherogenesis aging vessels	*ARID5B*-KO as atheroprotection	THP1 monocytes	*RHOT1*	0.37	10^{-4}	[43]
136	*Rhobtb3*	0.86	0.05	pulmonary hypertension fibrosis aging lung	norm	lung	*RHOBTB2*	−1.20	10^{-4}	[39]
137	*Rhobtb3*	0.86	0.05	pulmonary hypertension aging vessels	norm	lung	*RHOBTB1*	−0.28	10^{-3}	[39]
138	*Rln3*	−3.73	10^{-3}	pulmonary hypertension fibrosis aging lung	norm	lung	*RLN1*	0.56	10^{-2}	[39]
139	*Slfn13*	1.33	0.05	LPS-stimulated atherogenesis aging vessels	*ARID5B*-KO as atheroprotection	THP1 monocytes	*SLFN11*	−0.33	10^{-3}	[43]
140	*Slfn13*	1.33	0.05	pulmonary hypertension fibrosis aging lung	norm	lung	*SLFNL1*	−1.17	10^{-2}	[39]
141	*Slfn13*	1.33	0.05	pulmonary hypertension aging vessels	norm	lung	*SLFN12L*	−0.52	10^{-4}	[39]
142	*Spint1*	−1.16	10^{-3}	LPS-stimulated atherogenesis aging vessels	*ARID5B*-KO as atheroprotection	THP1 monocytes	*SPINT2*	0.31	10^{-3}	[43]
143	*Spint1*	−1.16	10^{-3}	pulmonary hypertension fibrosis aging lung	norm	lung	*SPINT1*	−0.82	10^{-2}	[39]
144	*Tnnt1*	2.80	0.05	pulmonary hypertension aging vessels	norm	lung	*TNNT2*	0.22	10^{-3}	[39]
145	*Tnnt1*	2.80	0.05	atrial fibrillation as heart aging	norm	auricle tissue	*TNNT3*	1.27	10^{-2}	[41]
146	*Ucn*	3.61	0.05	pulmonary hypertension aging vessels	norm	lung	*UCN3*	0.04	0.05	[39]
147	*Wsb1*	0.95	0.05	LPS-stimulated atherogenesis aging vessels	*ARID5B*-KO as atheroprotection	THP1 monocytes	*WSB1*	−0.33	10^{-3}	[43]
148	*Wsb1*	0.95	0.05	pulmonary hypertension aging vessels	norm	lung	*WSB2*	0.23	10^{-3}	[39]

Note: See the footnote to Table S1. LPS, lipopolysaccharide; *ARID5B*, AT-Rich Interaction Domain 5B gene.

Table S3. Effects of underexpression or overexpression of the human genes homologous to the novel PAG-related rat DEGs on ARD development.

No	Gene (NCBI Entrez)	Effect of changes in human gene expression on aging rates [Reference]			
		**Deficient (↓)**	**ARD**	**Excessive (↑)**	**ARD**
1	*AMY1A* (276)	within the retrospective clinical meta-analysis [86]: *AMYA1* deficit prevents Alzheimer's disease, which occurs in elderly people [87]	←	high *AMY1A* copy number is associated with a decreased hazard ratio of Alzheimer's dementia [86] which occurs in elderly people [87].	←
2	*AOX1* (316)	DNA methylation causing *low AOX1* might be biomarkers for prostate cancer risk [88]; age is the most important risk factor for prostate cancer [89].	→	within the human aging model using transgenic *Aox* Knock-In fruit fly: higher fecundity and fertility duration via slow sperm and eggs aging down [90]	←
3	*ASCL3* (56676)	ASCL3 deficiency was detected in l lymphoma, bladder, cervical, kidney and epithelial cancers [91], the risk of which increases with age [92, 133, 94, 124]	→	*ASCL3* overexpresses only in breast cancer [91], risks of which rises with post-reproductive aging [188]	→
4	*BANP* (54971)	within human disease model using mice: Banp-deficit as biomarker polycystic kidney disease [93], which is aging-related [94]	→	BANP-excess as biomarker for malnutrition [95], which causes aging-related diseases [96].	→
5	*BDKRB2* (624)	within human disease models using mice: Bdkrb2 deficiency slows atherogenesis down [97] that slows aging down too [98]	←	BDKRB2 excess could serve as an independent prognosticator in glioma [99], which is an aging-related disease [100]	→
6	*CARTPT* (9607)	within cellular models of human diseases: reduced insulin secretion [101], which is associated with aging per se [102].	→	within human disease models using transgenic mice with CARTPT excess: higher risks of both insulin resistance and diabetes [103], which are aging-related pathologies [104]	→
7	*CYP2J2* (1573)	according to 78% homology between the rat DEG Cyp2j10 in question and this human protein gene [105], deficit of which in cardiac tissue is a biomarker for patients with cardiomyopathy [106] as an age-related cardiovascular diseases [107] as the most common cause of death for the captive chimpanzee, who is the nearest wild congeners that diverged from their common ancestor with humans [195]	→	according to 78% homology between the rat DEG Cyp2j10 in question and this human protein gene [105], within human disease models using rats: Cyp2j2-excess suppressed endothelial senescence of retina [108]	←
8	*DEFB117* (245931)	according to orthology between the rat DEG Defb17 in question and this human *DEFB117* pseudogene [109], according to a retrospective clinical review: higher risks of atopic dermatitis [110] as skin immunological aging [111]	→	according to orthology between the rat DEG Defb17 in question and this human *DEFB117* pseudogene [109],within human disease models using monkeys: prevented hypertension [112], which causes vascular aging [25]	←
9	*EMX2* (2018)	within cohort clinical trials using RT-qPCR, colorectal tumor samples had decreased EMX2 expression levels [113], which ranks number one cause of cancer morbidity in women aged more than 65 years old [114]	←	overexpression of EMX2 led to an inhibition of tumor cell migration in colorectal cancer [113], which ranks number one cause of cancer morbidity in women aged more than 65 years old [114]	→
10	*FAT2* (2196)	mutational FAT2-defects contribute to better overall survival in lung cancer patients [115] that increases with immunosenescence [116]	←	within a retrospective clinical cohort review: high FAT2 expression strongly correlated with high immunosuppression in lung cancer patients [115] aggravating with immunosenescence [116]	→
11	*FCGR2B* (2213)	in South Asia and Africa, the human FCGR2B - deficient alleles are of the most frequent occurrence as protectors against infection [117], susceptibility to which increases with age as immunosenescence [118]	←	FCGR2B has been explored using the "C-type lectin-like molecule-1"/"Fc-domain" fusion protein as a target antigen for chemo-therapy against acute myeloid leukemia [119] as cellular senescence-related immunogenic disease [120]	→

Table S3 (continued).

No	Gene (NCBI Entrez)	Effect of changes in human gene expression on aging rates [Reference]			
		Deficient (↓)	ARD	Excessive (↑)	ARD
12	*FOSB* (2354)	deficit of any AP1-subunits (e.g., FOSB) reduces taste maintenance that is a symptom of aging [121]	→	may indicate haemangioendotheliomas [122], risks of which increases with immunosenescence [116]	→
13	*GPD1* (2819)	within cellular models of human cancerogenesis: GPD1 deficit is a biomarker for bladder cancer [123], risks of which is highest in 80-year-olds in both men and women [124]	→	within cellular models of human cancerogenesis: the GPD1 allosteric activator wedelolactone has significantly promoted apoptosis in bladder cancer [123], risks of which is highest in 80-year-olds in both men and women [124]	←
14	*HBD* (3045)	according to orthology between the rat DEG Hbb-b1 in question and this human gene [125], minor thalassemia (heterozygous hemoglobin deficit) protects against Alzheimer's disease [126], which occurs in elderly people [87]	←	according to orthology between the rat DEG Hbb-b1 in question and this human gene [125], HBD is significantly increased in patients with multiple sclerosis who have not received treatment [127] that occurs after 50 years in 5% of cases in line with the epidemiologic studies [128].	→
15	*HSPA1B* (3304)	within human age-related Parkinson's disease models using Hspa1b-knockout mice: increased vulnerable to the neurotoxic effects through dopaminergic neural degeneration [129]	→	within human longevity models using transgenic fruit fly carrying extra copies Hsp70 genes: higher longevity [130]as well as within the "haplotype vs human cell longevity" correlation review [131]	←
16	*ITM2A* (9452)	within the retrospective bioinformatics meta-analysis of the deferentially expressed genes (DEGs) related to chemotherapeutic relapse cervical carcinoma: ITM2A deficit was associated with poor survival in cervical adenocarcinoma [132], which is an age-related neoplasm [133]	→	within disease models using human breast cancer cells transfected with plasmid carrying extra ITM2A copy: the significant inhibition of the breast cancer cell proliferation, when breast cancer is the leading cause of mortality among women [134]	←
17	*KRT2* (3849)	within human disease models using rats: higher risk of Parkinson's disease [135], which is usually diagnosed in people over 65 years of age [136]	→	KRT2 expression elevates in intestinal adenomas as compared to normal epithelial cells [137], which usually developed in middle-aged and elderly patients, with a mean age of 62 years [138]	→
18	*MORN1* (79906)	within the retrospective bioinformatics meta-analysis of RNA-Seq data related to oral squamous cell carcinoma patients: MORN1 deficit can elevate survival in elderly patients with oral squamous cell carcinoma [139], which is an age-related disease [140]	←	MORN1 excess was found among DEGs within the microarrays study of patients with coronary artery disease [41], risk of which grows with the gender-dependent epigenetic clock-derived DNA methylation age acceleration (DNAmAA) [141] and the degree grows of premature hair graying [142]	→
19	*MYOM2* (9172)	within human disease models using rats administered with thyroid hormone: MYOM2 deficiency marks cardiac hypertrophy [143] as the most common cause of death for the captive chimpanzee, who is the nearest wild congeners that diverged from their common ancestor with humans [195]	→	MYOM2 excess was found among DEGs within the microarray of rats, who's hearts were subjected to ischemia [144], the risk of which increases with age [120]	→
20	*NFXL1* (152518)	SNP rs144169475 (4.1%, South America) in the NFXL1 gene is a nonsynonymous change N150K (at highly conserved residue that might be disfunction) associated with language impairment [145], which may be indicators for early automated diagnosis of the aging-related cognitive disorders [146]	→	a high level of NFXL1 expression in cerebellar structures might be relevant in light of an association between NFXL1 excess and cognitive disorder resistanse [145], which may indicate early automated diagnosis of the aging-related cognitive disorders [146]	←
21	*NMB (4828)*	NMB receptor antagonist, PD168368, prevents vascular calcification [147], which prevails in aging [148]	←	both NMB and its receptor upregulated in calcification of vascular smooth muscle cells [147], which is prevalent in aging [148]	→
22	*NMNAT1* (64802)	within human disease models using Nmnat1-knockout mice: severe degeneration of photoreceptors and select inner retinal neurons [149], which is an aging-related disease [150]	→	within human disease model using mice, recombinant human NMNAT1 injection reduced both infarct volume and blood-brain barrier permeability that improved functional outcome after ischemic stroke [151], which relates to aging through disruption and degeneration of cerebral microvasculatur [152]	←

Table S3 (continued).

No	Gene (NCBI Entrez)	Effect of changes in human gene expression on aging rates [Reference]			
		**Deficient (↓)**	**ARD**	**Excessive (↑)**	**ARD**
23	*PCP2* (126006)	within human disease models using transgenic mice: Pcp2-deficit is a marker of the polyglutamine (PolyQ) associated neurodegeneration such as Huntington's diseaseand spinocerebellar ataxia type 7 [153], which is aging-related [154]	→	within human disease model using transgene Pcp2 Knock-In mice, enhanced Purkinje cell survival, findings of which seems to be applicable in future regarding somewhat therapy against neuronal death and degeneration during aging [155]	←
24	*PDIA4* (9601)	within human disease models using Pdia4-knockout mice: reduced patient survival in lung cancer [156] risks of which irises with aging as lung aging [185]	→	PDIA4 excess correlates with poor prognosis in gliomas [157] which is aging-related dissease [100]	→
25	*PDYN* (5173)	within human aging models using Pdyn knockout mice: enhanced long-term depression as a form of synaptic plasticity contributing aging-related neurodegeneration [158]	←	within human aging models using Pdyn knockout mice: normal increasing Pdyn levels with aging impair cognitive function and increase anxiety [158]	→
26	*PLA2G2C* (391013)	within human disease model using Pla2g2d-null mice: higher risks of skin diseases (e.g., psoriasis and contact dermatitis) [159], older patients have a higher risk of greater concomitant pathology in psoriasis [160]	→	within human disease model using Pla2g2d-overexpressing transgenic mice: ameliorated skin diseases (e.g., psoriasis and contact dermatitis) [159] older patients have a higher risk of greater concomitant pathology in psoriasis [160]	←
27	*PLOD1* (5351)	within the human aging models using rats: adipose-derived stem cells subjected to Plod1-knockdown have therapeutic potential in aging-associated facial nerve regeneration [161]	←	according to clinical RNA-Seq meta- analysis, biomarker for the aging-related Alzheimer's disease [162]	→
28	*PTER* (9317)	within human renal disease model using mice with and cell lines of patients with these diseases as controls: Pter-silencing due to siRNA-interference reduces albuminuria-induced inflammatory and pro-fibrotic cytokines production [163], the absolute rate of albuminuria is higher in older adults [165]	←	within human renal disease model using mice with and cell lines of patients with these diseases as controls: PTER expression is significantly increasing in membranous nephropathy that may lead to albuminuria [163], which is the most important cause of the nephrotic syndrome in elderly patients over 65 years of age [164]	→
29	*PYGL* (5836)	within human liver disease model using conditional knockout mice: PYGL-deficiency results in glycogen storage disease along with liver enlargement and tumorigenesis [166], while in human aging models using nematode fed a high sugar diet: glycogen excess shortens lifespan [167]	→	PYGL excess as an independent factor for poor prognosis of gliomas [168], which is an ARD [100]	→
30	*RBM3* (5935)	within the cohort-based study: higher risks of relapse in urothelial bladder carcinoma [169], resistance to which increases with age in women [170]	→	within the cohort-based urothelial bladder carcinoma study: higher survival due to lesser cancer grade with absence of both distant metastasis and lymph node metastasis [169], whereas in women, resistance to this disease increases with age [170]	←
31	*RETSAT* (54884)	within human cancer models using mice: Retsat-knockdown increases proliferation, migration, and metastasis of cancer cells [173] its deficit is a biomarker of obesity [171] accelerating aging [172]	→	within human cancer models using mice: Retsat-excess reduces proliferation, migration, and metastasis of cancer cells [173], while the cancer risk increases with age [92, 133, 94]	←
32	*RHOBTB3* (22836)	within cellular models of human cancer: RHOBTB3-knockdown may exert inhibit the proliferation, migration, and metastasis of breast cancer cells [174], risks of which rises with post-reproductive aging [188]	←	within the cohort-based RNA-Seq data meta-analysis: improved post-chemotherapy survival in acute myeloid leukemia [175], which is an aggressive hematological disorder mainly affecting people of older age [176]	←

Table S3 (continued).

No	Gene (NCBI Entrez)	Effect of changes in human gene expression on aging rates [Reference]			
		**Deficient (↓)**	**ARD**	**Excessive (↑)**	**ARD**
33	*RLN3* (117579)	relaxin deficiency (by 40%) is one of the human fibrotic cardiomyopathy models using laboratory animals [177] that is a age-related cardiovascular disease [107] as the most common cause of death for the captive chimpanzee, who is the nearest wild congeners that diverged from their common ancestor with humans [195]	→	according to exhaustive review[178], via positive control for DNA damage repair and anti-apoptotic mechanisms, RLN3 excess prevents many aging-related diseases, e.g., anxiety, depression, memory dysfunction, and appetite loss	←
34	*SCEL* (8796)	within cellular models of human cancer, SCEL knockdown caused colorectal cancer cells to form invasive structures and to increase migration, when hepatic metastasis is the major cause of mortality in colorectal cancer [179]	→	high expressions associated with high-budding colorectal cancers [180], risks of which increases with aging increase [181], i.e. its ranks #1 cause of cancer morbidity in women over 65 years old [114]	→
35	*SLFN13* (146857)	within cellular models of human stress-dependent diseases: low SLFN13 activity may reduce stress-responsiveness [182], whereas cellular-senescence is a complex stress response [183]	→	within clinical RNA-Seq data, SLFN13-excess is a biomarker of the post-surgery early recurrence of lung cancer [184], risks of which increases with aging as a form of lung aging [185]	→
36	*SPINT1* (6692)	within the clinical RNA-Seq meta-analysis, the SPINT1-loss patients have very poor prognosis in non-small cell lung cancer as the leading cause of cancer-related death worldwide [186]	→	in clinical RNA-Seq data, SPINT1-excess biomarks for poor prognosis of breast cancer [187], risks of which rises with post-reproductive aging [188]	→
37	*TNNT1* (7138)	within human disease models using *Tnnt1*-knockout mice: decreased tolerance to muscle fatigue [189], , which is a common symptom in old age [190]	→	within clinical RNA-Seq data meta-analysis, TNNT1-excess is a marker of pancreatic cancer[191], peak age of which onset is 85-89 years in China [192]	→
38	*UCN* (7349)	within human disease models using rats: exogeneous urocortin in addition to its endogeneous deficient heals both musculoskeletal disorders and osteoporosis, which lead tofall-related injuries of bone and muscle in aging population [193]	→	within the exhaustive review: UCN excess is cardio protector in cardiac ischaemia, however, with the side effect of long-term use of hypertrophic cardiomyopathy [194], which is the most common cause of death for the captive chimpanzee, who is the nearest wild congeners that diverged from their common ancestor with humans [195]	←
39	*WSB1* (26118)	a new molecular WSB1-degrader found empirically can disturb the migration capacity of cancer cells as a promising anti-metastasis drug [196], whereas aging rises the metastatic ability of tumor cells [197].	←	within clinical RNA-Seq data, WSB1-excess contributes to premalignant cell states [198], risks of which rises with post-reproductive aging [188]	→

Note*:** Changes in gene expression: underexpression (↓) or overexpression (↑); ***ARD, effects on age-related diseases: aggravating (→) or alleviating (←). Human genes*: AMY1A*, amylase α1A; *AOX1*, aldehyde oxidase 1; *ASCL3*, achaete-scute family bhlh transcription factor 3; *BANP*, BTG3 associated nuclear protein (synonym: scaffold/matrix-associated region-1-binding protein); *BDKRB2*, bradykinin receptor b2; *: CARTPT*, cocaine and amphetamine regulated transcript (CART) prepropeptide; *CYP2J2*, cytochrome P450 family 2 subfamily j member 2; *DEFB117*, defensin β117 (pseudogene); *EMX2*, empty spiracles homeobox protein 2; *FAT2*, FAT atypical protocadherin 2; *FCGR2B*, Fc-γ receptor IIb. *FOSB*, activator protein 1 (AP-1) transcription factor subunit FosB proto-oncogene; *GPD1*, glycerol-3-phosphate dehydrogenase 1; *HBD*, hemoglobin subunit δ; *HSPA1B*, heat shock protein family A (Hsp70) member 1b; *ITM2A*, integral membrane protein 2A; *KRT2*, keratin 2; *MORN1*, morn repeat containing protein 1; *MYOM2*, myomesin 2; *NFXL1*, X-box binding nuclear transcription factor like 1; *NMB*, neuromedin-β; *NMNAT1*, nicotinamide nucleotide adenylyltransferase 1; *PCP2*, *P*urkinje cell protein 2; *PDIA4*, protein disulfide isomerase family A member 4; *PDYN*, prodynorphin; *PLA2G2C*, phospholipase A2, group IIC; *PLOD1*, procollagen-lysine, 2-oxoglutarate 5-dioxygenase 1;. *PTER*, phosphotriesterase-related protein; *PYGL*, glycogen phosphorylase l; *RBM3*, RNA binding motif protein 3; *RETSAT*, retinol saturase; *RHOBTB3*, Rho-related BTB domain-containing protein 3; *RLN3*, relaxin 3; *SCEL*, sciellin; *SLFN13*, schlafen-13; *SPINT1*, serine peptidase inhibitor, kunitz type 1; *TNNT1*, slow skeletal muscle troponin T1; *UCN*, urocortin; *WSB1*, WD repeat and SOCS-box-containing protein 1.

Section S1. Supplementary methods for DNA sequence analysis

Two DNA sequences S_{WT}={$s_{WT;-90}$…$s_{WT;i}$…$s_{WT;-1}$} and S_{MIN}={$s_{MIN;-90}$…$s_{MIN;i}$…$s_{MIN;-1}$}, each 70 bp in length, which textually represent two variants of an arbitrary promoter located upstream of the transcription start site (TSS, $s_{WT;0}$=$s_{MUT;0}$; $s_i \in$ {a, c, g, t}) were used as input data to our previously developed web service SNP_TATA_Comparator (http://beehive.bionet.nsc.ru/cgi-bin/mgs/tatascan_fox/start.pl) [219] in two textboxes, "Basic sequence" and "Editable sequence", respectively (Figure S1c). First, for each of these sequences S∈{S_{WT}, S_{MIN}}, an estimate "-ln(K_D(S))" of the TATA-binding protein (TBP) binding affinity for the corresponding variant of this promoter was obtained:

$$-\ln(K_D) = 10.9 - 0.2\ [\ln(K_{SLIDE}\ K_{STOP}\ K_{BEND}), \quad \mathbf{(1)}$$

where 10.9 (ln units) corresponds to the estimates of nonspecific TBP-DNA affinity (10 mM [250]; 0.2 is the stoichiometric coefficient [251]; -ln(K_{STOP}) is the empirical estimate of the affinity of the TBP for the most probable TBP-binding site by means of Bucher's criterion [252] among all possible 15-bp fragments of both DNA chains of the promoter analyzed, such as:

$$\boldsymbol{ln(K_{STOP})} = MAX\left\{\sum_{j=-1}^{13} \boldsymbol{w_{j;s_{i+j}}}\right\}; \quad (2)$$

where w_{js} as an element of the Bucher's matrix [252], which corresponds to the case of the nucleotide s located at the j-th position of the DNA sequence in question; MAX(ζ) is the highest ζ-value found.

In Eq. (1), -ln(K_{SLIDE}) is the empirical estimate of TBP affinity for this promoter during TBP sliding along DNA in the ±5-bp local environment around the most probable TBP-binding site, which is heuristically estimated as:

$$-\ln(K_{SLIDE}) = \mathbf{MEAN}\ \{0.8[TA] + 3.4\mu + 35.1\}, \quad \mathbf{(3)}$$

where [TA] is the weighted number of dinucleotide TA; μ is the arithmetic mean of the minor groove width of the DNA helix [253] of the TBP-binding site in question; 0.8, 3.4, and 35.1 are the linear regression coefficients optimized elsewhere [254]; MEAN(ζ) is the arithmetic mean of all observed ζ-values.

In Eq. (1), -ln(K_{BEND}) is the empirical estimate of TBP affinity for the most probable TBP-binding site during allosteric rearrangement of the B-helical DNA of this site by bending its axis at right angles to stabilize the TBP-promoter complex:

$$-\ln(K_{BEND}) = \mathbf{MEAN}\{0.9[TA, AA, TG, AG] + 2.5[TA, TC, TG] + 14.4\}, \quad \mathbf{(4)}$$

where 0.9, 2.5, and 14.4 are the linear regression coefficients as described elsewhere [254].

After that, the "-ln[K_D] values (Eqs. 1-4) go together with their standard error of mean (SEM) calculated according to all possible nucleotide substitutions, $s_{\bullet;j} \rightarrow \xi$, at each *j*-th position within the same ±5-bp local environment around the most probable TBP-binding site, namely:

$$SEM(S_\bullet)=[\ (\Sigma_{1\le i\le 26}\Sigma_{\xi\ \{a,c,g,t\}}[\ln(K_D(\{s_{\bullet;i-13}\ldots\xi\ldots s_{\bullet;i+12}\})/K_D(\{s_{\bullet;i-13}\ldots s_{\bullet;i+j}\ldots s_{\bullet;i+12}\})^2)]/(3*26)^{1/2} \quad \mathbf{(5)}$$

As an intermediate result, there are two paired estimates, "–ln(K_D(S_{WT})) ± SEM(S_{WT})" and "-ln(K_D(S_{MIN}))±SEM(S_{MIN})", calculated with the input sequences S_{WT} and S_{MIN} using Eqs. (1-5), which are statistically comparable with one another by means of Fisher's Z-test:

$$Z = \mathbf{abs}\ [\ln(K_{WT;D} / K_{MIN;D})] / [SEM(S_{WT})^2 + SEM(S_{MIN})^2]^{1/2}. \quad (6)$$

where Z is Fisher's Z-score as input for the corresponding procedure within the statistical package R [255], the output is the *p*-value of the probability rate of acceptance of the H_0-hypothesis "H_0: $K_D(S_{WT}) \neq K_D(S_{MIN})$".

Finally, the desired decision is made at its statistically significant level $\alpha < 0.05$ (where $\alpha = 1 - p$), as:

IF {*INEQUALITY* "–ln($K_{WT;D}$) > –ln($K_{MIN;D}$)" is statistically significant},

THEN {*DECISION* is "S_{MIN} provides an underexpression of a given gene in comparison with S_{WT} as a norm"};

ELSE [**IF** {*INEQUALITY* "–ln($K_{WT;D}$) < –ln($K_{MIN;D}$)" is statistically significant},

THEN {*DECISION* is "S_{MIN} provides an overexpression of a given gene in comparison with S_{WT} as a norm"},

OTHERWISE {*DECISION* is "change in the expression of this gene is insignificant"}.

Figure S1c shows this DECISION, such as: the text box "Result", the line "DECISION".

The web service SNP_TATA_Comparator [219] was independently used in a clinical study of pulmonary tuberculosis [256]

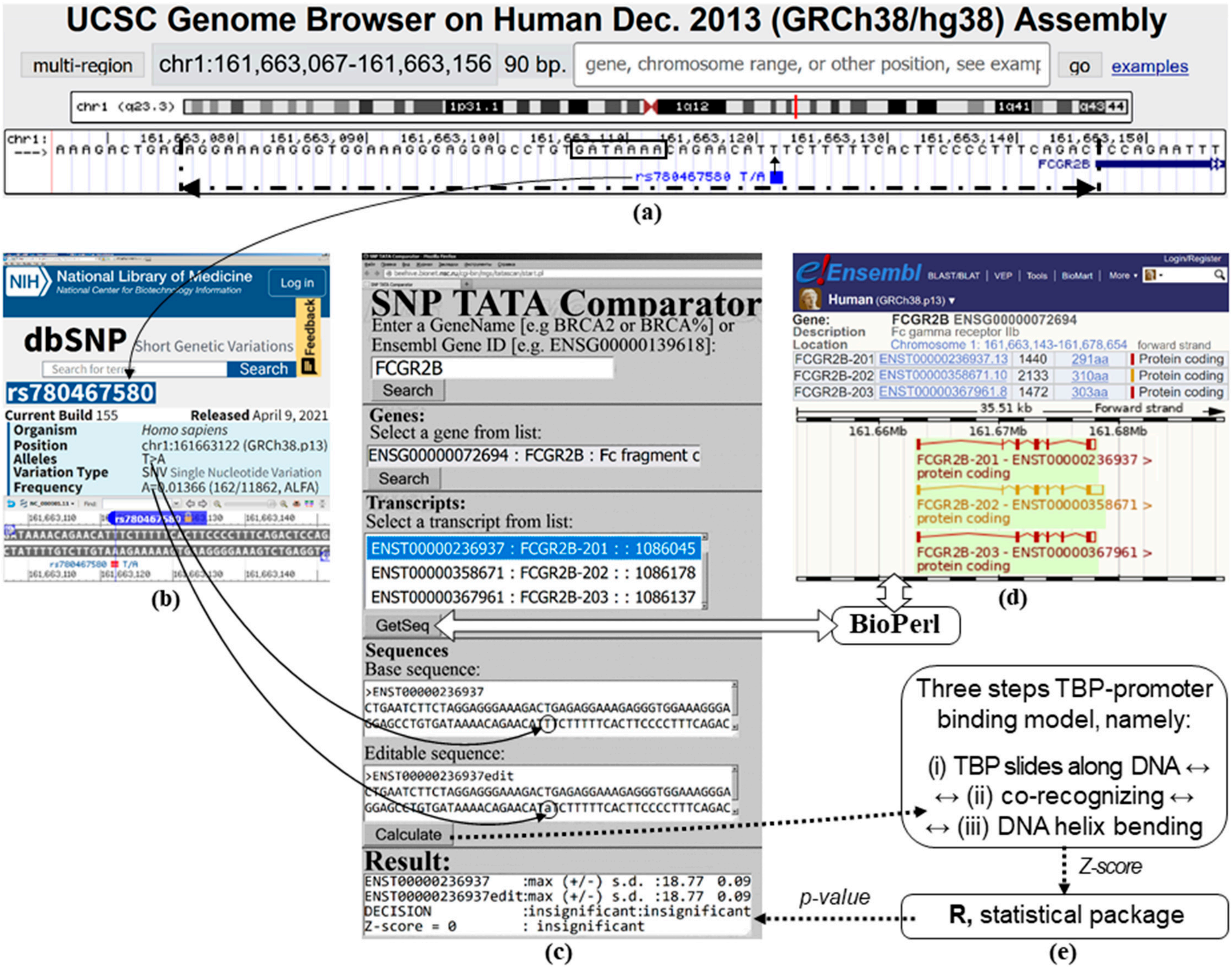

Figure S1. The result obtained using our web service SNP_TATA_Comparator [219] on only one SNP rs780467580 in the 70-bp proximal promoter of the human FCGR2B gene as the only common differentially expressed gene (DEG) related to susceptibility versus resistance to age-related diseases (ARDs) in subjects (humans and animal-based human ARD models). This SNP is in the current build (No. 155) of the dbSNP database [218] and seems to be neutral according to calculations with the use of this web service. Legend: (a) The UCSC Genome Browser [248] visualizes a 70-bp proximal promoter (double-headed dash-and-dot arrow) of the human *FCGR2B* gene in question, which contains only one TBP-binding site (framed) and only one SNP rs780467580, pointed to by the arrow. (b) The current build (No. 155) of the dbSNP database [218] describes the only SNP rs34166473 within the human *FCGR2B* gene promoters, each 70-bp in length. (c) Our prediction of an insignificant change in *FCGR2B* expression (textbox "Result", row 3) caused by SNP rs34166473 (circled) due its both alleles (ancestral and minor, as input data to two textboxes "Basic sequence" and "Editable sequence," respectively). (d) The Ensembl database [247] describes three protein-coding transcripts from the human *FCGR2B* gene located on the q arm (cytogenetic band 1g23.3) of human chromosome 1 according to the current assembly GRCh38/hg38.p13 of the human reference genome. Two double-headed open arrows depict how our web service SNP_TATA_Comparator [187] used the Bioperl toolkit [249] and retrieved the ancestral DNA sequence of the human *FCGR2B* promoter from the Ensembl database [247]. (e) With the use of the standard R toolbox [255], our web service implemented our empirical model of three-step binding between the human TBP and an arbitrary 70-bp proximal promoter before the transcription start site of the human gene being considered [251], namely: (i) TBP slides along promoter DNA [257] ↔ (ii) TBP stops at a potential TBP-binding site (TBP-site) [252, 258] ↔ the B-helical form DNA of this TBP-site bends at 90 degrees fixing the TBP-promoter complex [259-261], as observed experimentally [262]. We have previously used our publicly available development, SNP_TATA_Comparator [219], and manually analyzed 15,080 SNPs within 1585 proximal promoters, each 70 bp in length, located upstream of the transcription start sites of protein-coding transcripts from 534 human genes (for review, see [220]). The number of SNPs within these promoters varied from one to 64 (9.51 ± 0.21; mean ± SEM), with the 95% confidence interval being between 9 and 10; therefore, the existence of only one SNP rs34166473 within the examined region of the human FCGR2B gene promoter seems to suggest a statistically significant loss of SNPs ($p < 0.01$, binomial distribution). This is consistent with the commonly accepted criterion stating that natural selection fixes the vital optimum of the expression of this gene in humans. Hence, our prediction made using our web service SNP_TATA_Comparator [219] for the neutrality of this SNP (rs34166473) is consistent with the above criteria.

Table S4. BLAST-based [232] phylostratigraphic age indices (PAIs) of 39 human genes homologous to the behavior-related PAG-associated rat DEGs (Table 2) and 48 human FCGR2B-associated genes (Figure 5).

Human gene		The most recent common ancestor		Human gene		The most recent common ancestor		Human gene		The most recent common ancestor	
#	*Symbol*	Taxon	PAI	#	*Symbol*	Taxon	PAI	#	*Symbol*	Taxon	PAI
i	ii	iii	iv	i	ii	iii	iv	i	ii	iii	iv
(a) 39 human genes homologous to the novel behavior-related PAG-associated rat DEGs (Table 2)											
1	*FCGR2B*	Amniota	16	14	*BANP*	Bilateria	5	27	*RHOBTB3*	Eukaryota	1
2	*KRT2*	Amniota	16	15	*PYGL*	Bilateria	5	28	*GPD1*	Eukaryota	1
3	*RLN3*	Euteleostomi	12	16	*MYOM2*	Bilateria	5	29	*AOX1*	Eukaryota	1
4	*CYP2J2*	Euteleostomi	12	17	*ITM2A*	Bilateria	5	30	*PLOD1*	Eukaryota	1
5	*HBD*	Euteleostomi	12	18	*EMX2*	Eumetazoa	4	31	*RBM3*	Eukaryota	1
6	*NMB*	Euteleostomi	12	19	*FAT2*	Eumetazoa	4	32	*NMNAT1*	Eukaryota	1
7	*PDYN*	Euteleostomi	12	20	*PLA2G2C*	Eumetazoa	4	33	*PDIA4*	Eukaryota	1
8	*BDKRB2*	Euteleostomi	12	21	*PCP2*	Metazoa	3	34	*WSB1*	Cellular Organisms	1
9	*TNNT1*	Euteleostomi	12	22	*ASCL3*	Metazoa	3	35	*AMY1A*	Cellular Organisms	0
10	*UCN*	Euteleostomi	12	23	*SPINT1*	Metazoa	3	36	*RETSAT*	Cellular Organisms	0
11	*CARTPT*	Euteleostomi	12	24	*SCEL*	Opisthokonta	2	37	*MORN1*	Cellular Organisms	0
12	*FOSB*	Bilateria	5	25	*SLFN13*	Eukaryota	2	38	*PTER*	Cellular Organisms	0
13	*HSPA1B*	Bilateria	5	26	*NFXL1*	Eukaryota	1	39	*DEFB117*	ND	-
arithmetic mean estimate (MEAN) ± standard error of the mean (SEM): 5.32 ± 0.83											
(b) 48 human genes associated with *FCGR2B* as the most common ARD-linked DEG (Table 6) according to ANDSystem [227]											
1	*IL3*	Eutheria	19	17	*STAT3*	Euteleostomi	12	33	*RELA*	Eumetazoa	4
2	*IL13*	Eutheria	19	18	*MS4A1*	Euteleostomi	12	34	*TGFBR2*	Eumetazoa	4
3	*FCGR2B*	Amniota	16	19	*HDAC9*	Euteleostomi	12	35	*JUND*	Metazoa	3
4	*FCGR2A*	Amniota	16	20	*CD79A*	Gnathostomata	10	36	*VWF*	Metazoa	3
5	*FCGR3A*	Amniota	16	21	*TLR9*	Chordata	7	37	*CD36*	Metazoa	3
6	*FCGR3B*	Amniota	16	22	*CD40*	Chordata	7	38	*FAS*	Opisthokonta	2
7	*IL4*	Amniota	16	23	*INS*	Deuterostomia	6	39	*JUN*	Opisthokonta	2
8	*FCGR2C*	Amniota	16	24	*FOS*	Bilateria	5	40	*JUNB*	Opisthokonta	2
9	*FCGR1A*	Tetrapoda	15	25	*FOSB*	Bilateria	5	41	*SH2D1A*	Opisthokonta	2
10	*IFNG*	Tetrapoda	15	26	*GATA4*	Bilateria	5	42	*PTPN11*	Opisthokonta	2
11	*CD19*	Tetrapoda	15	27	*LGALS3*	Bilateria	5	43	*SYK*	Opisthokonta	2
12	*IL10*	Euteleostomi	12	28	*SYT1*	Bilateria	5	44	*INPP5D*	Eukaryota	1
13	*ITGAM*	Euteleostomi	12	29	*TGFB1*	Bilateria	5	45	*KARS1*	Eukaryota	1
14	*LYN*	Euteleostomi	12	30	*CRP*	Eumetazoa	4	46	*GRASP65*	Eukaryota	1
15	*KRT20*	Euteleostomi	12	31	*APCS*	Eumetazoa	4	47	*CDK11B*	Eukaryota	1
16	*WNK1*	Euteleostomi	12	32	*SMAD3*	Eumetazoa	4	48	*FASN*	Cellular Organisms	0
arithmetic mean estimate (MEAN) ± standard error of the mean (SEM): 7.92 ± 0.84											

Notes. ND: not detected; underlined, the human gene *FCGR2B* as the statistically significantly most common DEG of the old compared to young subjects (humans and animals). PAI, a gene's phylostratigraphic age index evaluated against the BLAST-based scale [232] using the freely available web service Orthoscape [229]; BLAST-based PAI scale: 0, Cellular organisms; 1, Eukaryota; 2, Opisthokonta; 3, Metazoa; 4, Eumetazoa; 5, Bilateria; 6, Deuterostomia; 7, Chordata; 8, Craniata; 9, Vertebrata; 10, Gnathostomata; 11, Teleostomi; 12, Euteleostomi; 13, Sarcopterygii; 14, Dipnotetrapodomorpha; 15, Tetrapoda; 16, Amniota; 17, Mammalia; 18, Theria; 19, Eutheria; 20, Euarchontoglires; 21, Primates; 22, Haplorrhini; 23, Simiiformes; 24, Catarrhini; 25, Hominoidea; 26, Hominidae; 27, Homininae; 28, Homo.

© 2023 by the authors. Submitted for possible open access publication under the terms and conditions of the Creative Commons Attribution (CC BY) license (http://creativecommons.org/licenses/by/4.0/).
